# Montmorillonite K-10 catalyzed synthesis of Hantzsch dihydropyridine derivatives from methyl arenes *via in situ* generated ammonia under microwave irradiation in neat conditions†

**DOI:** 10.1039/d4ra04990j

**Published:** 2024-08-27

**Authors:** Vishal Singh, Khushbu Rajput, Sundaram Singh, Vandana Srivastava

**Affiliations:** a Department of Chemistry, Indian Institute of Technology (BHU) Varanasi 221005 UP India vsrivastava.apc@iitbhu.ac.in +91-9453365168

## Abstract

An expeditious, efficient, and environmentally friendly approach has been established for the synthesis of diverse Hantzsch 1,4-dihydropyridine derivatives utilizing montmorillonite K-10 as a catalyst in solvent-free conditions. The procedure entails the reaction of methyl arynes as a sustainable surrogate of aryl aldehydes, active methylene compounds, and urea hydrogen peroxide (UHP) as an oxidising agent as well as a source of ammonia under microwave irradiation, facilitated by montmorillonite K-10.

## Abstract 2

Solvent-free synthesis of Hantzsch 1,4-dihydropyridine catalyzed by montmorillonite K-10 involves oxidation of methyl arenes and *in situ*-generated ammonia from urea hydrogen peroxide under microwave irradiation.

## Graphical abstract

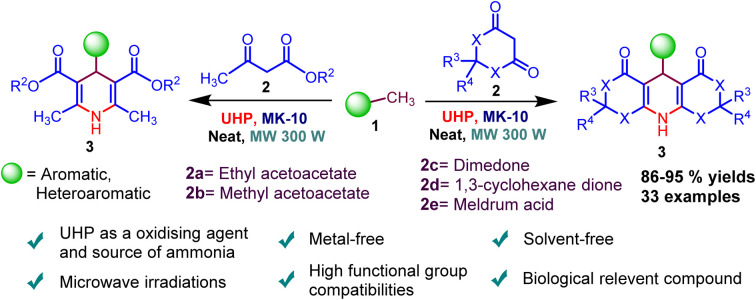

## Introduction

**Fig. 1:**
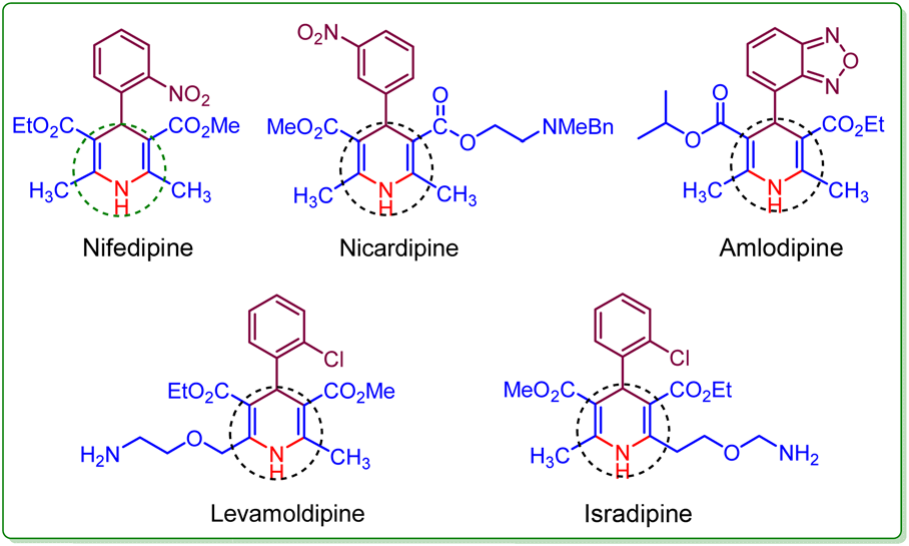
Biologically active 1,4-dihydropyridines as a key functional moiety.

Heterocyclic compounds with six members are integral in organic chemistry.^1^ Within *N*-heterocycles, the 1,4-dihydropyridine (1,4-DHP) framework and its derivatives have additional significance because of their diverse pharmacological properties.^2,3^ The 1,4-dihydropyridine core structure finds extensive use in various biological and therapeutic realms.^4^ The 1,4-DHP nucleus is a constituent of various drugs like Nifedipine, Nicardipine, Levamoldipine, Isradipine, and Amlodipine (Fig. 1).^5,6^ Amlodipine, known as a calcium channel blocker, is employed in managing hypertension. Additionally, it serves as an active intermediate in significant organic transformations.^7^

Currently, green chemistry principles present significant opportunities for enhancing the synthesis of biologically and pharmacologically active heterocyclic frameworks through multicomponent reactions (MCRs).^8,9^ Multi-component reactions are particularly advantageous as they generally afford good yields and offer rapid access to a wide range of heterocyclic frameworks for their diverse applications.^10^ In this context, the advancement of solvent-free conditions for one-pot multicomponent coupling reactions has garnered significant attention. The solvent-free multi-component reaction technique is an environmentally conscious approach, which opens several possibilities for conducting quick organic synthesis, functional group conversions, and also the elimination of solvents thus mitigating pollution in organic synthesis.^11–13^

Traditionally, the synthesis of 1,4-dihydropyridine (1,4-DHP) and its derivatives typically involved the conventional Hantzsch reaction.^14^ This process encompasses a one-pot multicomponent synthesis, where two molecules of β- ketoester, an aldehyde, and an *N*-donor ligand (such as ammonia, ammonium salts, or derivatives of ammonia) undergo cyclo-condensation in EtOH under refluxing conditions.^15^ Due to the significant importance of the 1,4-dihydropyridine motif, a wide array of catalysts including homogeneous, heterogeneous, and diverse nanomaterials are utilized in its synthesis.^16–30^

Despite several advantages of these reported methods, there are some limitations such as harsh reaction conditions, non-ecofriendly solvents, high reaction temperature, longer reaction times and low to moderate yields. Therefore, the development of simple, efficient, eco-compatible and environmentally friendly methods is still in demand for the synthesis of 1,4 DHPs containing molecules. The current research focused on employing eco-friendly and environmentally conscious chemical methods for the green synthesis of modified Hantzsch 1,4-dihydropyridines, utilizing methyl arenes as a sustainable alternative to acyl precursors.^31,32^ Urea hydrogen peroxide (UHP) is a solid oxidizing agent, and it offers a significant benefit in oxidation processes by generating non-toxic commercial urea as a byproduct.^33,34^ Montmorillonite K-10 (MK-10) is an efficient, versatile, solid, inexpensive, non-toxic, and odourless organo-heterogeneous catalyst.^35^ MK-10 has been documented as a catalyst for conducting a variety of chemical conversions.^36–39^ The use of MK-10 as heterogeneous catalysts is preferred over homogeneous catalysts due to the easy isolation and disposal off from the reaction mixture. In green chemistry, using environmentally benign reaction conditions and safe chemicals is essential for synthesizing important organic compounds.

The employment of microwave (MW) technology primarily aligns with environmentally friendly green methods^40,41^ and MW in solvent-free reactions has emerged as a vital aspect of synthetic organic chemistry.^42–44^ Moreover, microwave technique has gained significant attention in organic synthesis due to their ability to accelerate chemical reactions, enhance selectivity, improve product yields, and promote atom economy, thereby minimizing by-product generation compared to conventional heating methods.^45,46^

**Scheme 1:**
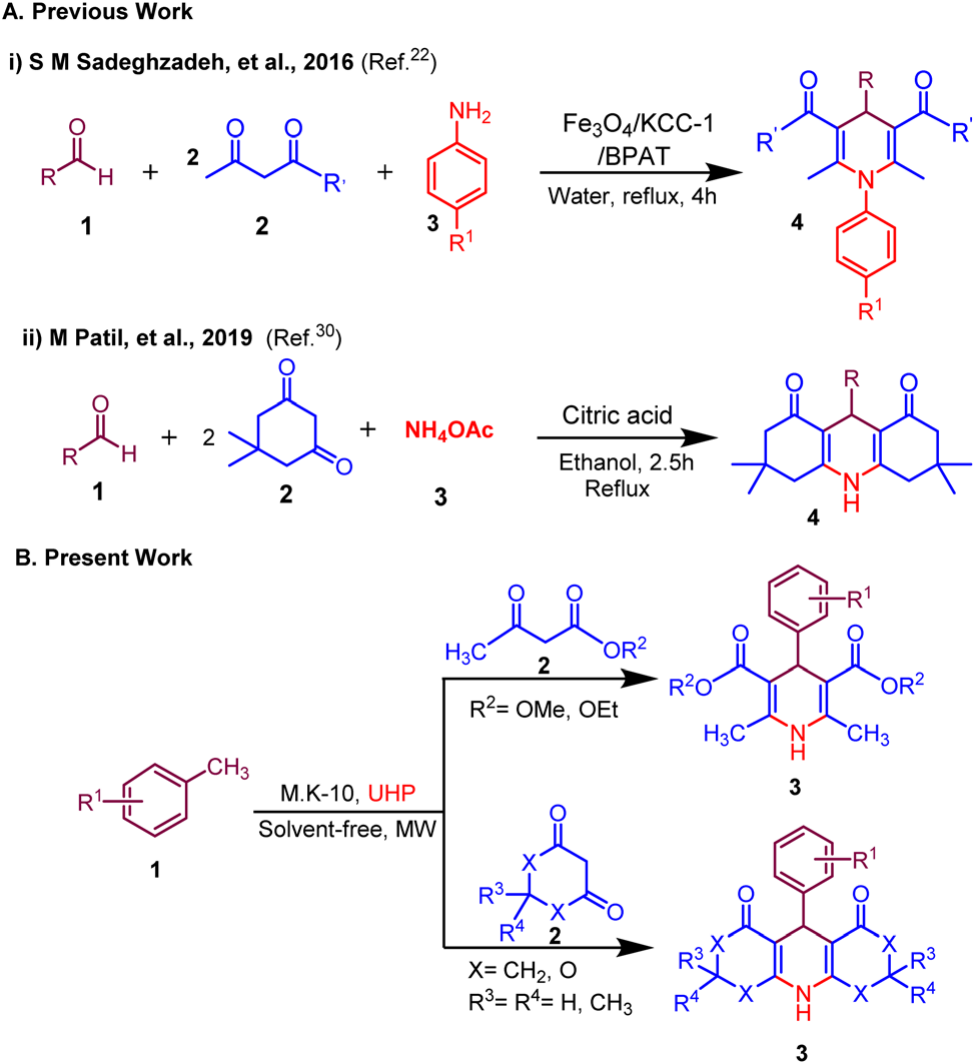
Synthesis of 1,4-dihydropyridines; (A) previous work and (B) present work.

Building on our efforts to develop greener approaches for the synthesis of biologically relevant molecules herein, we disclose a novel and efficient MK-10 catalyzed synthesis of 1,4-dihydropyridine derivatives from methyl arenes *via in situ* generated ammonia in neat conditions under microwave irradiation (Scheme 1). This method provides a straightforward experimental setup with swift reaction times, excellent yields and distinctive selectivity, the use of a reusable heterogeneous acid catalyst and a filtration-only work-up. To, the best of our knowledge, there is no report available for the synthesis of 1,4-dihydropyridines using methyl arenes as a sustainable surrogate of aldehyde precursor and UHP as an oxidant as well as a source of ammonia.

## Results and discussion

In our initial trial, for the optimization of reaction conditions, toluene 1a (1.0 mmol), ethyl acetoacetate 2a (3.0 mmol) and UHP (2.0 mmol) were selected as starting materials and using MK-10 (10 mg) as catalyst under microwave irradiation to synthesize specifically the of model compound 3a. The model reaction was subjected to various parameters at a randomly fixed temperature of 50 °C. The outcomes of the screening studies are presented in (Table 1). The polar protic solvents namely water, ethanol, and methanol gave 40–45% yield of the desired product 3a at 200 W in 60 min (Table 1, entries 1–3), while the polar aprotic solvents such as DMF, 1,4 dioxane gave 38–50% yields in 60 min (Table 1, entries 4 and 5). To improve the greener context of the synthesis, the reaction was performed under solvent-free conditions surprisingly, it gave a 60% yield in 30 min (Table 1, entry 6). Next, we have optimised the microwave power by increasing the microwave power from 200 MW to 350 MW. The maximum yield 75% of the product 3a was obtained at 300 MW power in 20 min. Further increase in microwave power no significant change in the yield was observed. Subsequently, the influence of temperature on the desired product 3a was examined at different temperature ranges of 60–80 °C (Table 1, entries 10 and 11). The temperature rise had a direct effect on the product yield, with 80% yield achieved at 60 °C within 20 minutes. However, further elevation of the temperature did not lead to an enhancement in the yield. The variation in catalyst loading, ranging from 10 to 30 mg, resulted in a notable increase in yield, indicating that 20 mg of catalyst was adequate to achieve the maximum 88% yield (Table 1, entry 13). Subsequently, the quantity of UHP was investigated in the ongoing optimization of solvent, temperature, and catalyst loading. By increasing the amount of UHP from 2 to 5 mmol, 95% yield of the desired product 3a was obtained with 4 mmol of UHP within 15 minutes (Table 1, entry 16). To check the importance of a catalyst the reaction was carried out in the absence of a catalyst, and it gave only 10% yield in 15 min. Thereafter the reaction was performed at room temperature, only 20% yield was obtained even in 60 min. On the other hand, when the reaction was carried out conventionally (heating only) less than 5% yield was observed (Table 1, entries 17–20). This suggests that toluene 1a, (1.0 mmol), ethyl acetoacetate 2a, (3 mmol) and UHP (4 mmol) with MK-10 (20 mg) as a catalyst at 60 °C in MW, 300 W under solvent-free is the optimal condition for 1,4-dihydropyridine 3a and the structure of was identified by ^1^H, ^13^C and HRMS spectra.

**Table 1:** Screening of parameters for the synthesis of dihydropyridine 3a^a^

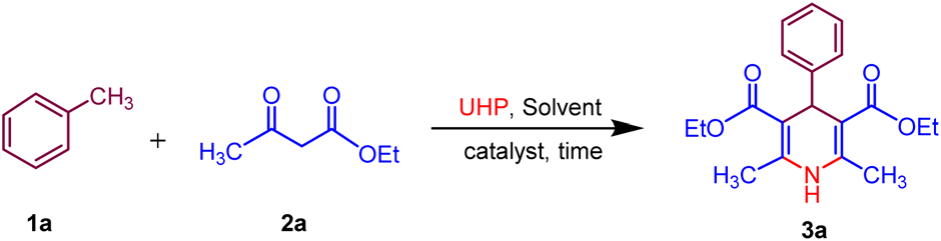							
Entry	Solvent	UHP (mmol)	MK-10 (mg)	MW power (W)	Temp. (°C)	Time (min)	Yield^b^ (%)
1	Water	2	10	200	50	60	40
2	EtOH	2	10	200	50	60	45
3	MeOH	2	10	200	50	60	40
4	DMF	2	10	200	50	60	50
5	Dioxane	2	10	200	50	60	38
6	—	2	10	200	50	30	60
7	—	2	10	250	50	30	68
8	—	2	10	300	50	30	75
9	—	2	10	350	50	30	75
10	—	2	10	300	60	20	80
11	—	2	10	300	80	20	80
12	—	2	15	300	60	20	83
13	—	2	20	300	60	20	88
14	—	2	30	300	60	20	88
15	—	3	20	300	60	15	91
16	—	4	20	300	60	15	95
17	—	5	20	300	60	15	95
18	—	4	—	300	60	15	10
19	—	4	20	300	r.t	60	20
20	—	4	20	—	60	60	<5

a Reaction conditions: toluene 1a (1.0 mmol), ethyl acetoacetate 2a (3.0 mmol) and UHP with MK-10 as a catalyst, in solvent (2 ml) under microwave-irradiation.

b Isolated yield.

**Scheme 2:**
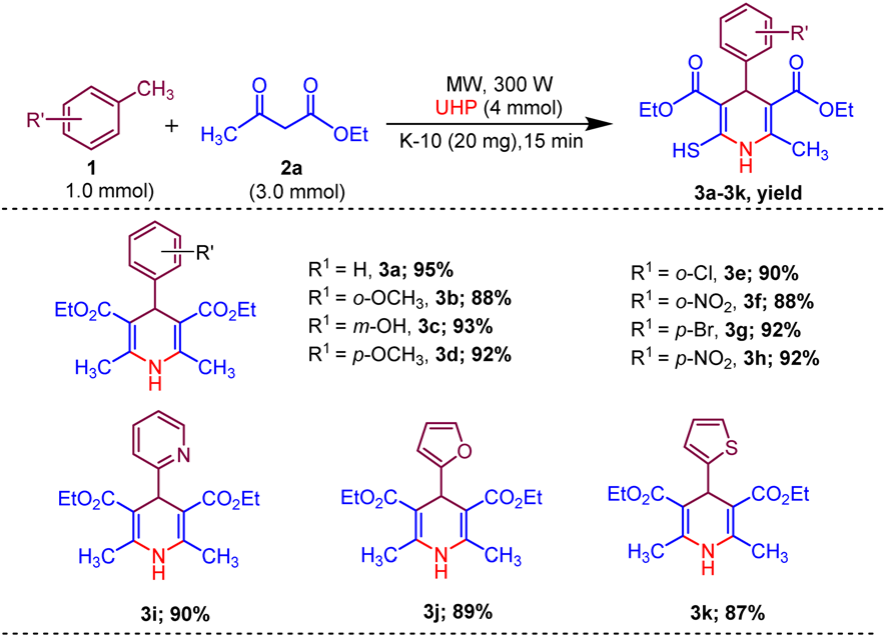
Substrate scope of MK-10 catalysed synthesis of 1,4-dihydropyridines from ethyl acetoacetate.

**Scheme 3:**
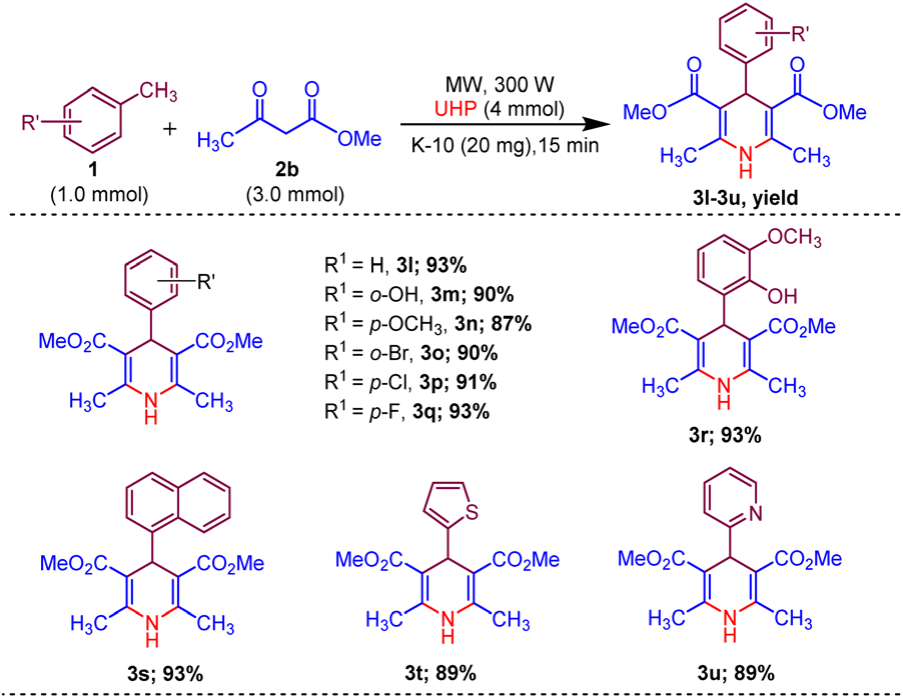
Substrate scope of MK-10 catalysed synthesis of 1,4-dihydropyridines from methyl acetoacetate.

**Scheme 4:**
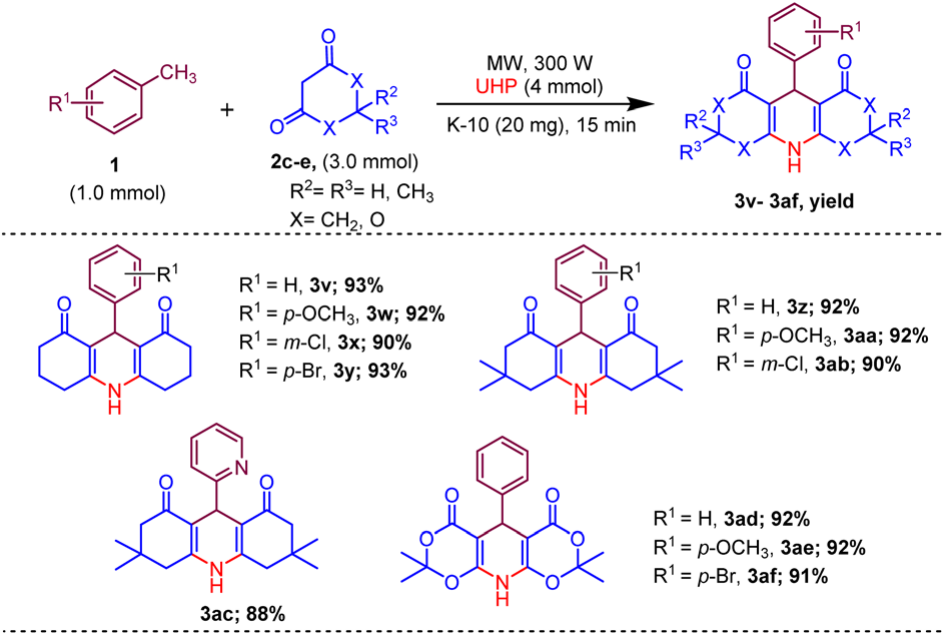
Substrate scope of MK-10 catalysed synthesis of 1,4-dihydropyridines from cyclic active methylene compounds.

Afterwards, to broaden the range of substrates, a variety of methyl arenes were treated with acyclic active methylene compounds (*e.g.* ethyl acetoacetate, methyl acetoacetate) and cyclic active methylene compounds (*e.g.* 1,3 cyclohexanedione, dimedone and meldrum acid) with UHP and catalyst MK-10 for the synthesis of dihydropyridines under the optimal condition. Toluene and various substituted methyl arenes, incorporating electron-donating and electron-withdrawing groups at *ortho*, *para* and *meta*, positions, yielded the desired product in high to excellent yields under optimized reaction conditions. Methyl heteroarenes have also adopted this transformation, which proceeded well and the cyclocondensation products were obtained with good yields (Schemes 2–4).

**Scheme 5:**
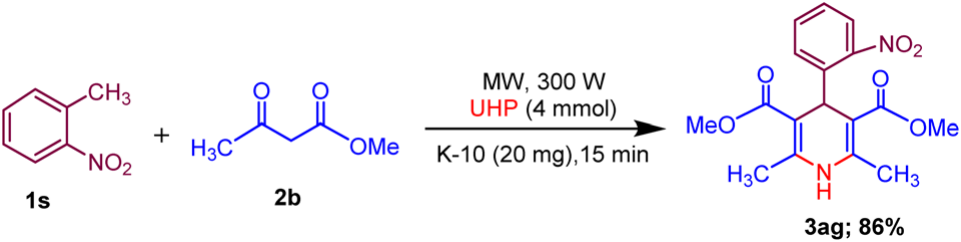
Synthesis of Nifedipine drug (3ag).

**Fig. 2:**
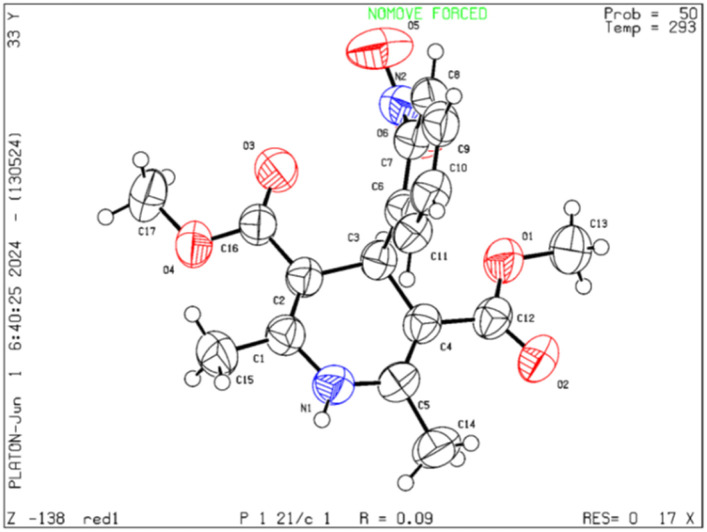
ORTEP representation crystal structure of product 3ag.

Moreover, the strategy was biocompatible and was employed to create a Nifedipine drug with 75% yield (3ag), used to treat high blood pressure and control angina (chest pain) (Scheme 5), which is confirmed by ^1^H NMR, ^13^C NMR, mass spectra and was also exposed to X-ray diffraction analysis in order to completely reveal its structural composition (Fig. 2) (see details Fig. S1 in the ESI†).

**Scheme 6:**
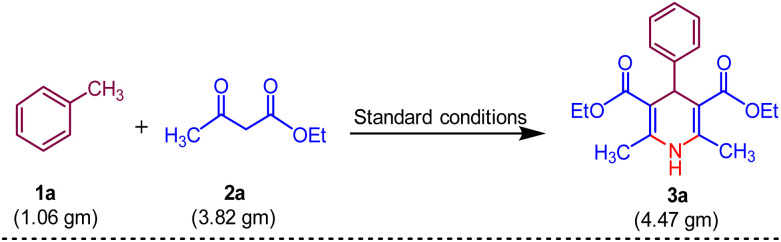
Gram-scale procedure for the synthesis of 3a.

To verify the potential synthetic use of the proven methodology for 1,4-dihydropyridine 3a, we experimented at the gramme level (Scheme 6). Toluene 1a (10.0 mmol, 1.06 gm), ethyl acetoacetate 2a (30.0 mmol, 3.82 g), UHP (40 mmol, 4.47 gm) and MK-10 (200 mg) were utilised in the process, yielding the pure product 3a with 88% yield.

### Controlled experiments

**Scheme 7:**
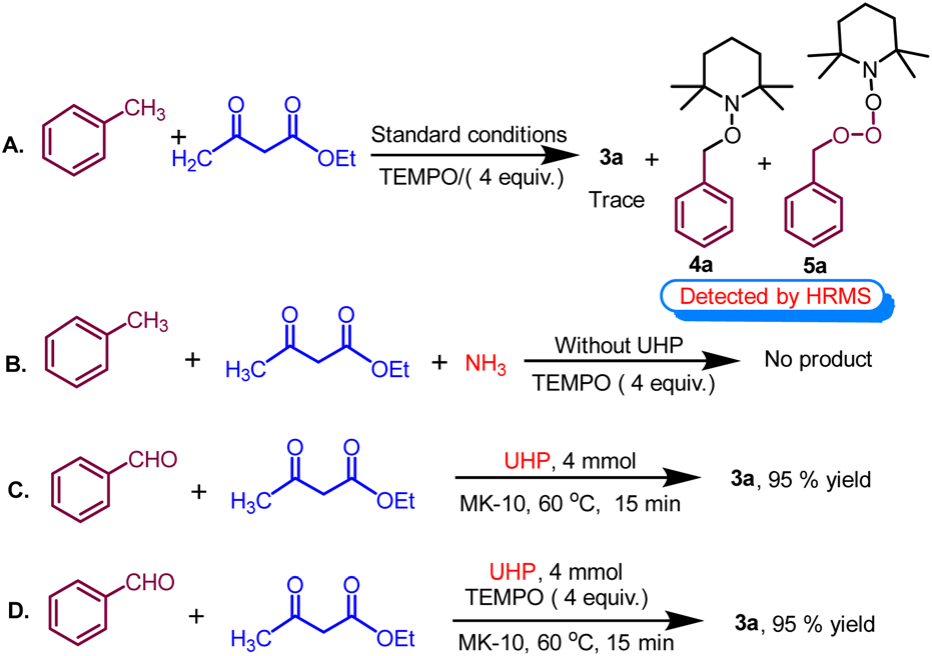
Control experiments in support of the mechanism.

To elucidate the likely reaction mechanism, few controlled experiments were performed under optimized reaction conditions, as shown in (Scheme 7). In this study, conducting the reaction in the presence of radical scavenger TEMPO (2,2,6,6-tetramethylpiperidin-1-yl)oxy and (Scheme 7A) resulted in less than 5% yield of the product 3a and TEMPO adduct 4a and 5a are formed which are detected by HRMS data (see Fig. S2 and S3 in the ESI†). This suggests that the formation of 1,4-dihydropyridine likely proceeds through a radical mechanistic pathway. Subsequently, the control reaction proceeded with toluene, ethyl acetoacetate, and ammonia in the absence of UHP, failing to yield the desired product. This outcome suggests that toluene cannot undergo oxidation to benzaldehyde without the presence of UHP, highlighting the indispensability of UHP for the initial step (Scheme 7B). Following this, the control reaction was conducted using benzaldehyde, ethyl acetoacetate, and UHP under optimized conditions, resulting in a highly successful reaction with a yield of 95% (Scheme 7C).

This suggests that the breakdown of UHP produced hydrogen peroxide (H_2_O_2_) and urea. Afterwards, H_2_O_2_ serves as an oxidant that converts toluene into benzaldehyde while the remaining urea served as a supplier of ammonia in the cyclocondensation reaction. Interestingly, when the experiment was repeated with radical scavengers such as BHT and TEMPO (Scheme 7D), no notable alteration in the yield was observed. This suggests the formation of 1,4-tetrahydropyridines through a non-radical mechanistic pathway after the oxidation of methyl arenes.

### Reaction mechanism

**Fig. 3:**
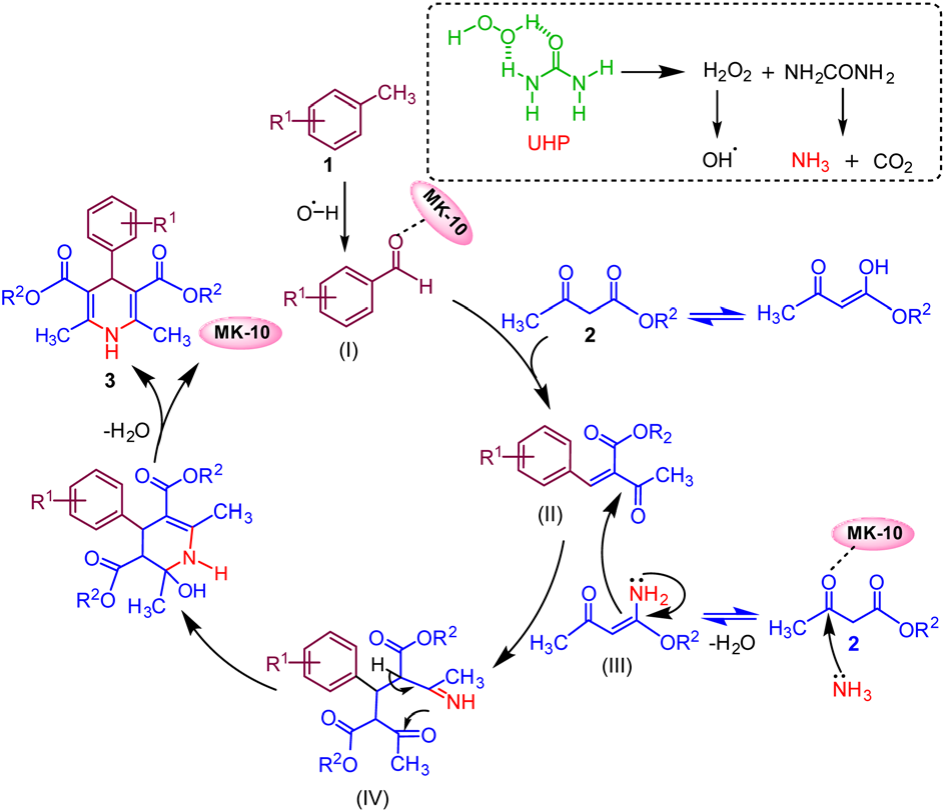
Proposed reaction mechanism.

On the basis of control experiments along with previously reported literature,^5,47^ a plausible mechanism for the reaction is anticipated, as depicted in (Fig. 3). The reaction is initiated by the oxidation of methyl arenes (1) with UHP that selectively produces aldehyde derivative intermediate (I) (confirmed by ^1^H NMR data, (see Fig. S4, Page S6 in the ESI†).^48^ Montmorillonite K-10 serves as a potent solid acid catalyst, featuring both Lewis and Brønsted acid sites.^35,49^

The catalyst MK-10 polarised the carbonyl group of the aldehyde, which subsequently condenses with one molecule of active methylene compound (2) *via* Knoevenagel condensation reaction and forms an intermediate (II). The ammonia which is generated during oxidation of toluene from UHP combines with the second molecule of active methylene compound and gives intermediate ester enamine (III). In the subsequent step, intermediate (II) and intermediate (III) combine through Michael's addition to generate intermediate (IV), which then undergoes intramolecular cyclization. This is followed by the elimination of a water molecule to yield the target molecule (3).

### Catalyst recycling

**Fig. 4:**
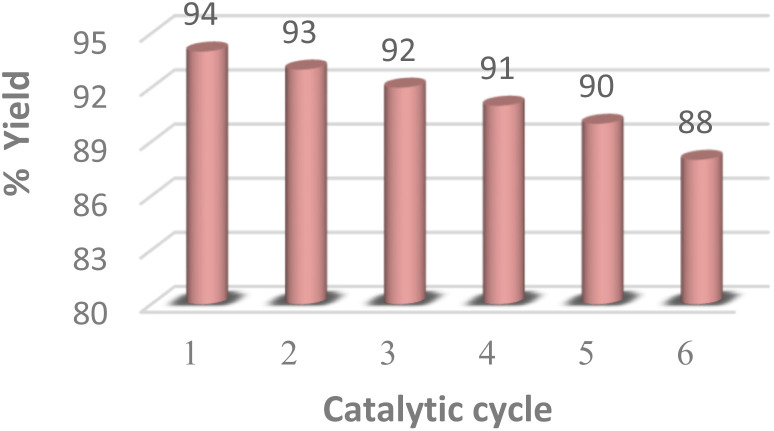
Reusability of MK-10 catalyst for the synthesis of 1,4-DHP derivative 3a.

The reusability of the MK-10 catalyst was also investigated using the optimized reaction conditions for up to five cycles (Fig. 4). After the completion of the reaction, the catalyst was separated by filtration and washed with ethyl acetate (3 × 5 mL) then it was dried at 100 °C for 10 h, shows that the clay can be recycled several times without any appreciable loss in activity and selectivity.

## Conclusions

An efficient, straightforward, and environmentally friendly approach has been developed to synthesize Hantzsch dihydropyridine derivatives. The utilization of microwave assistance proved indispensable in achieving the desired products in only fifteen minutes. The use of the heterogeneous catalyst MK-10 along with microwave irradiation insignificantly cuts down on reaction times and energy expenses thereby encouraging its industrial utilization. MK-10 was recycled and reused for six consecutive cycles with no notable decline in catalytic efficiency. Furthermore, the suggested approach generates minimal amounts of reaction waste. Simple set-up and work-up, good yields and elimination of solvent considerably reduce reaction times and energy expenses and minimizing the operational cost of the methodology.

## Data availability

The data supporting this article have been included as part of the ESI.† Crystallographic data for [3ag] has been deposited at the [CCDC] under [2360373].

## Author contributions

Vishal Singh: methodology, investigation, data curation, formal analysis and writing draft; K. Rajput: investigation, data curation and analysis; S. Singh: analysis and review; Vandana Srivastava: supervision, conceptualization, resources, review, and final editing.

## Conflicts of interest

There are no conflicts to declare.

## Supplementary Material

RA-014-D4RA04990J-s001

RA-014-D4RA04990J-s002
