# Supplementary material for: Montmorillonite K-10 catalyzed synthesis of Hantzsch dihydropyridine derivatives from methyl arenes via in situ generated ammonia under microwave irradiation in neat conditions
Source: RSC Adv. 2024 Aug 27;14(37):27086–91. doi: 10.1039/d4ra04990j (PMC11348851; doi:10.1039/d4ra04990j)
Supplement: RA-014-D4RA04990J-s001 [file RA-014-D4RA04990J-s001.pdf]

Supplementary Information (SI) for RSC Advances.
This journal is © The Royal Society of Chemistry 2024

Supporting information

Montmorillonite K-10 Catalyzed Synthesis of Hantzsch Dihydropyridine Derivatives from Methyl Arenes via in situ Generated Ammonia under Microwave Irradiation in Neat Cnditions

Vishal Singh, Khushbu Rajput, Sundaram Singh, Vandana Srivastava*

*Department of Chemistry, Indian Institute of Technology (BHU)
Varanasi, 221005 (India)

Email Id: vsrivastava.apc@itbhu.ac.in

LIST OF CONTENTS: page no.

1. General Information S2

2. Experimental procedure for the synthesis of 1,4-dihydropyridines S2

3. Structure determination via X-ray crystallographic analysis of (**3 ag**) S3

4. Controle experiments S6

5. Analytical data of products S8

6. ^{1}H, ^{13}C, ^{19}F NMR & Mass spectra of products S17

7. References S51

1. General Information

All chemicals and solvents were purchased from Sigma Aldrich and used without purification. Melting points were measured on the Stewart melting point apparatus in one side open capillary and are uncorrected. The progress of the reaction was monitored by thin-layer chromatography on a glass plate coated with silica gel G-234 and florosent silica gel. UV lamp and iodine chamber was used for the visualization of the reaction spot. High-Resolution Mass Spectrometry (HRMS) was performed using a SCIEX X500R QTOF (TOF-MS) system. ^{1}H and ^{13}C NMR spectra were recorded on Bruker Avance 500 MHz spectrometer in DMSO d_6 and $CdCl_3$ using TMS as internal standard 500 MHz (^{1}H) and 126 MHz (^{13}C). All chemical shifts were reported in ppm with reference to the DMSO peak (2.50 for ^{1}H and 39.50 for ^{13}C NMR). All coupling constants are reported in hertz (Hz). Abbreviations are, s: singlet, d: doublet, t: triplet, q: quartet, bs: broad singlet, dd: double doublet. All products synthesized were confirmed by using melting point, ^{1}H and ^{13}C NMR and comparison with the literature reports. The structure of the products was determined by X-ray crystallography (Rigaku XtaLAB Synergy-I). The names of all products were generated using the PerkinElmer ChemBioDraw Ultra v.16.0.1.4 software package.

2. Experimental procedures for the synthesis of 1,4-dihydropyridines

A mixture of appropriate methyl arene (1.0 mmol) active methylene compound (3.0 mmol) UHP (4 mmol) and MK-10 (20 mg) were placed in a 20-mL pressurised vials with ‘‘snap-on’’ cap and irradiated in the microwave using 300 W power at 60 °C for 15 min. The completion of the reaction was monitored with TLC, after completion, the reaction mixture was cooled to room temperature and diluted with ethyl acetate to dissolve the product and the catalyst was removed by filtration. The organic layer underwent washing with water, dried over Na_2SO_4 and subsequent concentrated under vacuum. The resulting crude product underwent further purification via recrystallization using ethanol to obtain the pure product **(3)**.

3. Structure determination via X-ray crystallographic analysis of dimethyl 2,6-dimethyl-4-(2-nitrophenyl)-1,4-dihydropyridine-3,5-dicarboxylate (3ag)

The yellow single crystals of the compound **3ag** suitable for X-ray crystallography were obtained when the saturated solution of **3ag** in ethanol was prepared and the resulting solution was slowly evaporated.

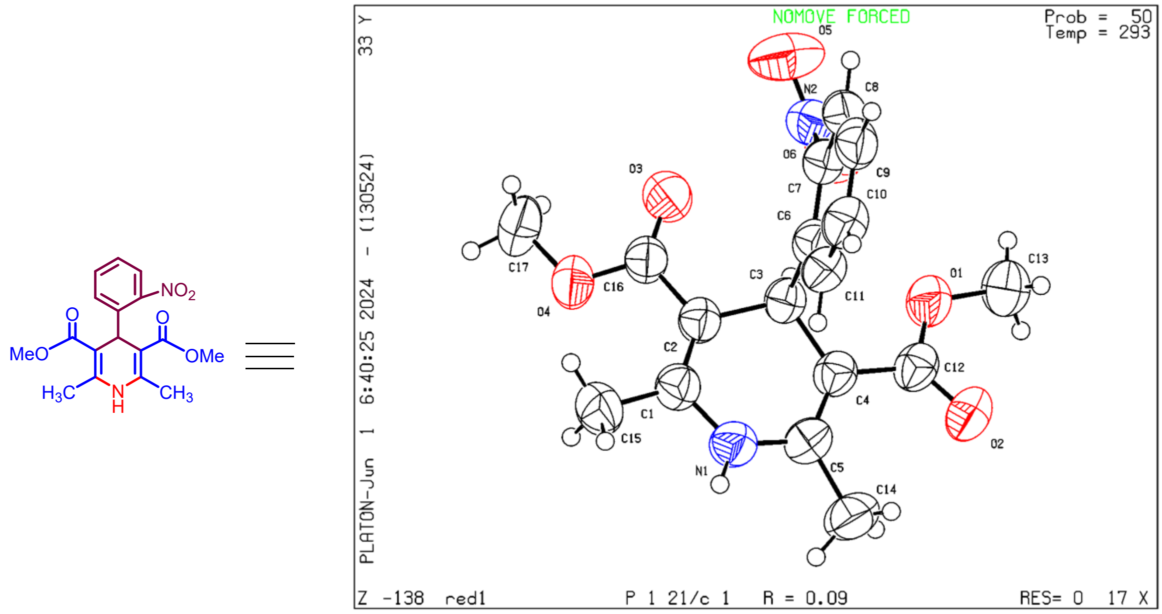

Fig. S1 ORTEP representation crystal structure of product **3ag** (ellipsoid contours of probability levels are 50%)

Table S1 Crystal data and structure refinement of product **3ag** (**CCDC: 2360373**)

Identification code	red 1
Empirical formula	$C_{17}H_{18}N_2O_6$
Formula weight	346.33
Temperature/K	293(2)
Crystal system	monoclinic
Space group	$P2_1/c$
a/Å	10.9599(3)
b/Å	10.3802(4)
c/Å	14.7603(4)
α/°	90
β/°	92.621(3)
γ/°	90
Volume/Å^3	1677.46(9)
Z	4
ρ_{calc}g/cm^3	1.371
μ/mm^{-1}	0.885
F(000)	728.0
Crystal size/mm^3	? × ? × ?
Radiation	Cu Kα (λ = 1.54184)
2Θ range for data collection/°	8.076 to 144.378
Index ranges	-13 ≤ h ≤ 13, -12 ≤ k ≤ 12, -12 ≤ l ≤ 18
Reflections collected	17221
Independent reflections	3296 [R_{int} = 0.0939, R_{sigma} = 0.0702]
Data/restraints/parameters	3296/0/230
Goodness-of-fit on F^2	1.106
Final R indexes [I>=2σ (I)]	R_1 = 0.0873, wR_2 = 0.2781
Final R indexes [all data]	R_1 = 0.1041, wR_2 = 0.2991
Largest diff. peak/hole / e Å^{-3}	1.04/-0.31

Bond Lengths for red 1.

Atom	Atom	Length/Å	Atom	Atom	Length/Å
C1	N1	1.390(4)	C3	C6	1.545(4)
C1	C2	1.344(4)	O3	C16	1.194(4)
C1	C15	1.497(5)	C4	C5	1.348(5)
N1	C5	1.374(5)	C4	C12	1.463(5)
O1	C12	1.327(4)	O4	C16	1.325(4)
O1	C13	1.422(5)	O4	C17	1.441(5)
C2	C3	1.528(4)	C5	C14	1.503(5)
C2	C16	1.467(4)	C6	C7	1.401(4)
N2	O5	1.214(4)	C6	C11	1.386(4)
N2	O6	1.212(4)	C7	C8	1.391(5)
N2	C7	1.454(4)	C8	C9	1.359(5)
O2	C12	1.225(4)	C9	C10	1.386(5)
C3	C4	1.529(4)	C10	C11	1.374(5)

Bond Angles for red 1.

Atom	Atom	Atom	Angle/˚	Atom	Atom	Atom	Angle/˚
N1	C1	C15	112.4(3)	C4	C5	N1	119.8(3)
C2	C1	N1	119.0(3)	C4	C5	C14	126.7(3)
C2	C1	C15	128.5(3)	C7	C6	C3	126.8(3)
C5	N1	C1	123.7(3)	C11	C6	C3	118.0(3)
C12	O1	C13	116.7(3)	C11	C6	C7	115.2(3)
C1	C2	C3	121.9(3)	C6	C7	N2	122.5(3)
C1	C2	C16	124.8(3)	C8	C7	N2	115.4(3)
C16	C2	C3	113.2(3)	C8	C7	C6	122.0(3)
O5	N2	C7	117.5(3)	C9	C8	C7	120.4(3)
O6	N2	O5	122.7(3)	C8	C9	C10	119.3(3)
O6	N2	C7	119.5(3)	C11	C10	C9	119.6(3)
C2	C3	C4	110.7(2)	C10	C11	C6	123.3(3)
C2	C3	C6	108.7(2)	O1	C12	C4	111.9(3)
C4	C3	C6	109.6(2)	O2	C12	O1	121.0(3)
C5	C4	C3	121.6(3)	O2	C12	C4	127.0(3)
C5	C4	C12	121.0(3)	O3	C16	C2	123.0(3)
C12	C4	C3	117.3(3)	O3	C16	O4	121.9(3)
C16	O4	C17	116.3(3)	O4	C16	C2	114.9(3)
N1	C5	C14	113.5(3)				

4. Controle Experioments

In this study, conducting the reaction in the presence of radical scavenger TEMPO (2,2,6,6-tetramethylpiperidin-1-yl)oxy and (Scheme S1, A) resulted in less than 5% yield of the product **3a** and TEMPO adduct **4a** and **5a** are formed which are detected by HRMS data **(Fig. S2, S3**). This suggests that the formation of 1,4-dihydropyridine likely proceeds through a radical mechanistic pathway. Subsequently, the control reaction proceeded with toluene, ethyl acetoacetate, and ammonia in the absence of UHP, failing to yield the desired product. This outcome suggests that toluene cannot undergo oxidation to benzaldehyde without the presence of UHP, highlighting the indispensability of UHP for the initial step (Scheme S1, B). Following this, the control reaction was conducted using benzaldehyde, ethyl acetoacetate, and UHP under optimized conditions, resulting in a highly successful reaction with a yield of 95% (Scheme S1, C). This suggests that the breakdown of UHP produced hydrogen peroxide (H_2O_2) and urea. Afterwards, H_2O_2 serves as an oxidant that converts toluene into benzaldehyde. while the remaining urea served as a supplier of ammonia in the cyclocondensation reaction. The aforementioned findings elucidated the dual function of UHP in both the oxidation of methyl arene to aldehyde and as a source of ammonia. Interestingly, when the experiment was repeated with radical scavenger TEMPO (Scheme S1, D), no notable alteration in the yield was observed. This suggests the formation of 1,4-tetrahydropyridines through a non-radical mechanistic pathway subsequent to the oxidation of methyl arenes.

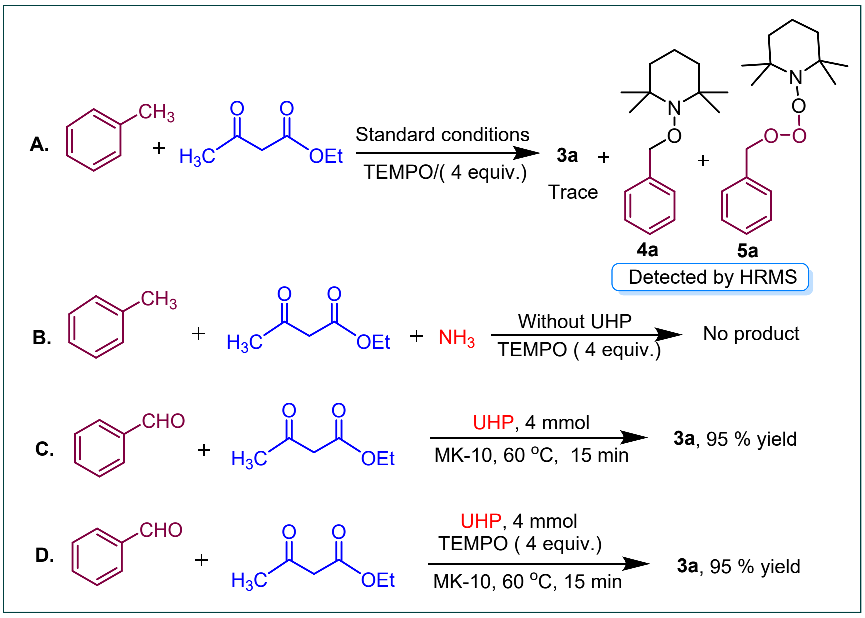

Scheme S1 Control experiments in support of the mechanism

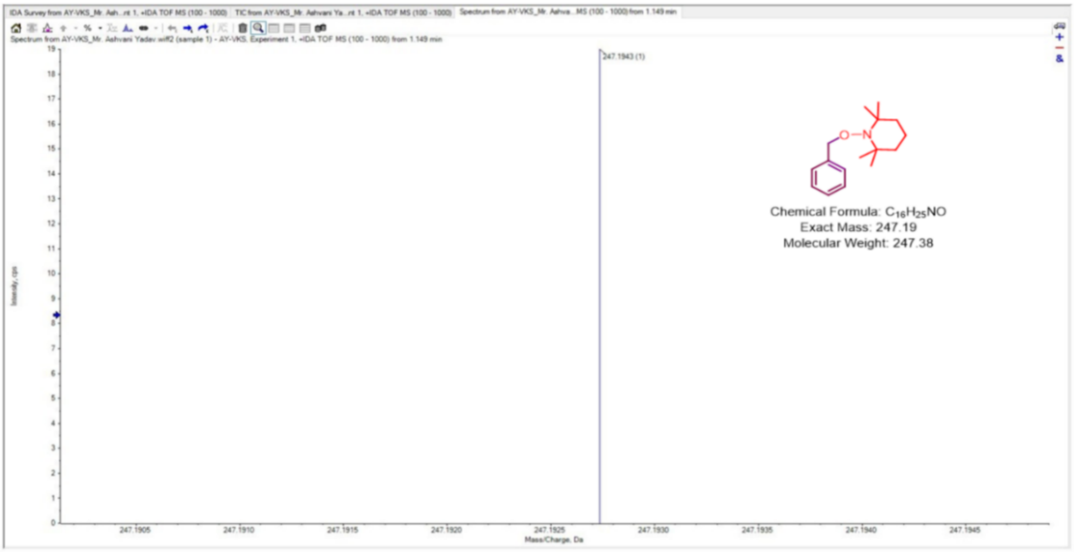

Fig. S2 HRMS of adduct **4a**

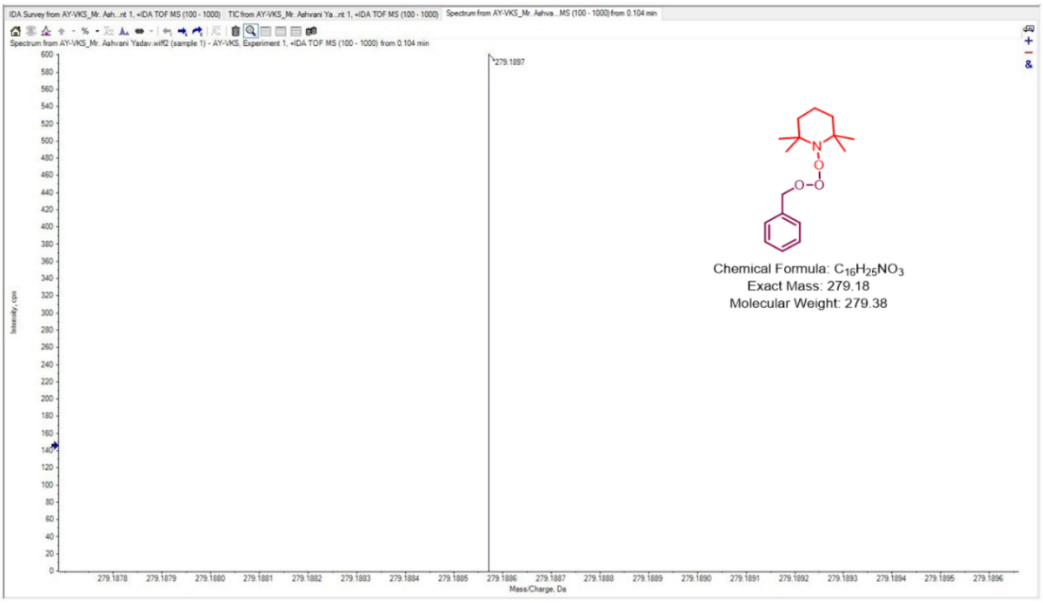

Fig. S3 HRMS of adduct **5a**

Benzaldeyde: (1) ^{1}H NMR (500 MHz, $CDCl_3$): δ 9.85 (s, 1H), 7.73 (d, 2H), 7.46-7.33 (t, 3H).

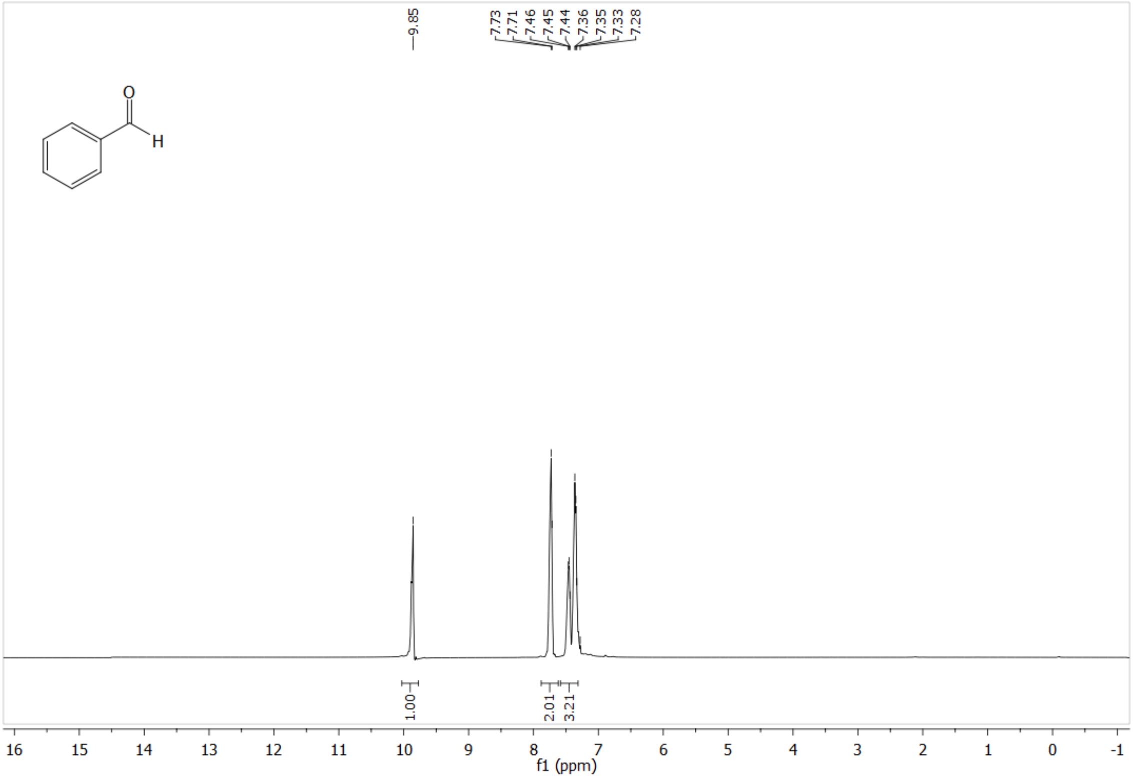

Fig. S4 ^{1}H NMR of intermediate 1 (Aldehyde)

5. Analytical data for [3a-3ag]

Diethyl 2,6-dimethyl-4-phenyl-1,4-dihydropyridine-3,5-dicarboxylate (3a) Light yellow solid; yield 94%; m.p. 155−157 °C; **^{1}H NMR** (500 MHz, DMSO-d_6): δ 8.96 (s, 1H), 7.34 (d, 2H), 7.28 (t, 3H), 4.45 (s, 1H), 4.23 (q, 4H), 2.20 (s, 6H), 1.34 (t, 6H) **^{13}C NMR** (126 MHz, DMSO-d_6): δ 170.0, 144.3, 140.6, 128.8, 127.0 126.9, 89.6, 62.3, 34.7, 17.8, 15,5; **HR-MS** (ESI) for $C_{19}H_{23}NO_4$ (m/z) $[M + H]^+$ calcd: 330.1627, found: 330.1645.

Diethyl 4-(2-methoxyphenyl)-2,6-dimethyl-1,4-dihydropyridine-3,5-dicarboxylate (3b) Yellow solid; yield 88%; m.p. 150-153 °C; **^{1}H NMR** (500 MHz, DMSO-d_6): δ 9.11 (s, 1H, NH), 7.62 (d, 1H), 7.36 (t, 1H), 7.19 (t, 1H), 7.15 (d, 1H), 5.09 (s, 1H), 4.39 (q, 4H), 3.96 (s, 3H, OCH_3), 2.26 (s, 6H, CH_3), 1.54 (t, 6H); **^{13}C NMR** (126 MHz, DMSO-d_6): δ 170.9, 154.7, 146.5, 132.5, 130.8, 129.3, 120.6, 111.5, 87.1, 61.3, 55.6, 28.9, 18.0, 14.2; **HR-MS** (ESI) for $C_{20}H_{25}NO_5$ (m/z) $[M + H]^+$ calcd: 360.1733, found: 360.1738.

Diethyl 4-(3-hydroxyphenyl)-2,6-dimethyl-1,4-dihydropyridine-3,5-dicarboxylate (3c) Yellow solid; yield 93%; m.p. 135-137 °C; **^{1}H NMR** (500 MHz, DMSO-d_6) δ 9.68 (s, 1H, NH), 8.67 (t, 1H), 8.37 (d, 1H), 7.33 (s, 1H), 5.12 (s, 1H), 4.39 (q, 4H), 3.39 (s, 1H), 2.28 (s, 6H, CH_3), 1.58 (t, 6H, CH_3); **^{13}C NMR** (126 MHz, DMSO-d_6): δ 170.3, 157.9, 147.3, 140.9, 129.9, 119.9, 117.5, 115.0, 89.5, 60.0, 35.6, 17.6, 14.5; **HR-MS** (ESI) for $C_{19}H_{23}NO_5$ (m/z) $[M + H]^+$ calcd : 346.1576, found: 346.1567.

Diethyl 4-(4-methoxyphenyl)-2,6-dimethyl-1,4-dihydropyridine-3,5-dicarboxylate (3d) Yellow solid; yield 92 %; m.p. 165 °C; **^{1}H NMR** (500 MHz, DMSO- d_6): δ 9.23 (s, 1H, NH), 7.45 (d, 2H), 6.98 (d, 2H), 5.10 (s, 1H), 4.40 (q, 4H), 3.97 (s, 3H), 2.27 (s, 6H, CH_3), 1.55 (t, 6H, CH_3); **^{13}C NMR** (126 MHz, DMSO-d_6): δ 170.1, 159.2, 144.9, 130.2, 129.5, 113.1, 117.6, 89.4,

61.5, 56.0, 34.7, 16.3, 14.4; **HR-MS** (ESI) for $C_{20}H_{25}NO_5$ (m/z) $[M + H]^+$ calcd: 360.1733, found: 360.1719

Diethyl 4-(2-chlorophenyl)-2,6-dimethyl-1,4-dihydropyridine-3,5-dicarboxylate (3e) Yellow solid; yield 90%; m.p. 120-122 °C; **^{1}H NMR** (500 MHz, DMSO-*d₆*): δ 9.32 (s, 1H, NH), 7.47 (d, 1H), 7.39-7.34 (m, 2H), 7.32 (d, 1H), 5.12 (s, 1H), 4.40 (q, 4H), 2.26 (s, 6H, CH_3), 1.54 (t, 6H); **^{13}C NMR** (126 MHz, DMSO-d_6): δ 173.4, 145.1, 137.7, 131.3, 129.0, 127.4, 126.1, 86.9, 59.0, 29.8, 16.6, 13.4; **HR-MS** (ESI) for $C_{19}H_{22}ClNO_4$ (m/z) $[M + H]^+$ calcd: 364.1237, found: 364.1223.

Diethyl 2,6-dimethyl-4-(2-nitrophenyl)-1,4-dihydropyridine-3,5-dicarboxylate (3f) Brown solid; yield 88%; m.p. 170 ^{o}C; **^{1}H NMR** (500 MHz, DMSO-d_6): 9.52 (s, 1H, NH), 8.16 (d, 1H), 7.81 (t, 1H), 7.65 (d, 1H), 7.59 (t, 1H), 5.23 (s, 1H), 4.25 (q, 4H), 2.39 (s, 6H, CH_3), 1.53 (t, 6H); **^{13}C NMR** (126 MHz, DMSO-d_6): δ 172.4, 149.7, 144.6, 134.5, 131.2, 131.0, 130.7, 123.2, 88.3, 61.8, 30.7, 14.7; **HR-MS** (ESI) for $C_{19}H_{22}N_2O_6$ (m/z) $[M + H]^+$ calcd: 375.1478, found: 375.1491

Diethyl 4-(4-bromophenyl)-2,6-dimethyl-1,4-dihydropyridine-3,5-dicarboxylate (3g) Yellow solid; yield 92%; m.p. 164 -166°C; **^{1}H NMR** (500 MHz, DMSO-d_6): *δ* 9.33 (s, 1H, NH), 7.65 (d, 2H), 7.35 (d, 2H), 5.18 (s, 1H), 4.29 (q, 4H), 2.21 (s, 6H), 1.55 (t, 6H); **^{13}C NMR** (126 MHz, DMSO-d_6): δ 170.2, 144.4, 138.3, 131.6, 128.8, 124.4, 90.4, 59.5, 35.0, 18.0, 14.3 **HR-MS** (ESI) for $C_{19}H_{22}BrNO_4$ (m/z) $[M + H]^+$ calcd: 408.0732, found: 408.0735

Diethyl 2,6-dimethyl-4-(4-nitrophenyl)-1,4-dihydropyridine-3,5-dicarboxylate (3h) Brown solid; yield 92%; m.p. 116-118 ^{o}C; **^{1}H NMR** (500 MHz, DMSO-d_6): *δ* 9.54 (s, 1H, NH), 8.00 (d, 2H), 7.66 (d, 2H), 5.22 (s, 1H), 4.41 (q, 4H), 2.31 (s, 6H), 1.55 (t, 6H); **^{13}C NMR** (126 MHz,

DMSO-d_6): δ 171.8, 149.7, 145.6, 144.3, 128.9, 123.4, 89.7, 61.6, 34.8, 16.3, 14.4; **HR-MS** (ESI) for $C_{19}H_{22}N_2O_6$ (m/z) $[M + H]^+$ calcd: 375.1478, found: 375.1469.

Diethyl 2',6'-dimethyl-1',4'-dihydro-[2,4'-bipyridine]-3',5'-dicarboxylate (3i) Brown solid; yield 90%; m.p.194 °C; **^{1}H NMR** (500 MHz, DMSO-d_6): δ 9.54 (s, 1H, NH), 8.41 (d, 2H,), 7.73 (t, 1H), 7.45 (d, 1H), 7.30 (t, 1H), 5.19 (s, 1H, CH), 4.34 (q, 4H), 2.27 (s, 6H, CH_3), 1.48 (t, 6H, CH_3); **^{13}C NMR** (126 MHz, DMSO-d_6): δ 170.3, 157.3, 146.1, 144.9, 144.8 140.5, 129.9, 119.9, 121,1, 91.7, 59.3, 35.9, 16.4, 14.3; **HR-MS** (ESI) for $C_{18}H_{22}N_2O_4$ (m/z) $[M + H]^+$ calcd: 331.1579, found: 331.1587

Diethyl 4-(furan-2-yl)-2,6-dimethyl-1,4-dihydropyridine-3,5-dicarboxylate (3j) Yellow solid; yield 89%; m.p. 236-238 °C; ^{1}H NMR (500 MHz, DMSO-d_6): δ 9.32 (s, 1H, NH), 7.27 (d, 1H), 6.39 (t, 1H), 6.20 (d, 1H), 5.12 (s, 1H), 4.38 (q, 4H), 2.26 (s, 6H), 1.54 (t, 6H); **^{13}C NMR** (126 MHz, DMSO-d_6): δ 170.8, 156.0, 146.4, 140.3, 114.5, 110.5, 87.1, 61.2, 36.5, 17.9, 14.2; HR-MS (ESI) for $C_{17}H_{21}NO_5$ (m/z) $[M + H]^+$ calcd: 320.1420, found: 320.1425.

Diethyl 2,6-dimethyl-4-(thiophen-2-yl)-1,4-dihydropyridine-3,5-dicarboxylate (3k) Light yellow; Yield 87%; m.p. 170-172 ^{o}C; **^{1}H NMR** (500 MHz, DMSO-d_6): δ 9.32 (s, 1H, NH), 7.26 (d, 1H), 6.40 (t, 1H), 6.19 (d, , 1H), 5.16 (s, 1H), 4.38 (q, 4H), 2.23 (s, 6H), 1.53 (t, 6H); **^{13}C NMR** (126 MHz, DMSO-d_6): δ 170.4, 153.3, 142.3, 135.8, 127.0, 125.3, 91.7, 59.4, 37.6, 16.4, 14.4; **HR-MS** (ESI) for $C_{17}H_{21}NO_4S$ (m/z) $[M + H]^+$ calcd: 336.1191, found: 336.1178.

Dimethyl 2,6-dimethyl-4-phenyl-1,4-dihydropyridine-3,5-dicarboxylate (3l) yellow solid; yield 93%; m.p. 188-190 ^{o}C; **^{1}H NMR** (500 MHz, DMSO-d_6): δ 9.12 (s, 1H, NH), 7.43 (d, 2H), 7.20-7.13 (m, 3H), 5.09 (s, 1H), 3.85 (s, 6H), 2.26 (s, 6H) **^{13}C NMR** (126 MHz, DMSO-d_6): δ

169.3, 143.7, 140.3, 128.7, 127.6 126.5, 89.8, 54.3, 34.4, 18.0; **HR-MS** (ESI) for $C_{19}H_{23}NO_4$; HR-MS (ESI) for $C_{17}H_{19}NO_4$ (m/z) $[M + H]^+$ calcd: 302.1314, found: 302.1325.

Dimethyl 4-(2-hydroxyphenyl)-2,6-dimethyl-1,4-dihydropyridine-3,5 dicarboxylate (3m) Yellow solid; yield 90%; m.p. 198-200 °C; **^{1}H NMR** (500 MHz, DMSO-d_6): δ 9.12 (s, 1H), 7.28 (2, 1H), 7.15 (d, 1H), 6.86 (d, 1H), 6.38 (s, 1H), 5.10 (s, 1H), 3.71 (s, 6H), 2.23 (s, 6H) **^{13}C NMR** (126 MHz, DMSO-d_6): δ 170.4, 151.1, 143.5, 130.9 130.7, 129.9, 119.5, 116.3, 86.2, 52.3, 28.1, 16.4; **HR-MS** (ESI) for $C_{19}H_{23}NO_4$ **HR-MS** (ESI) for $C_{17}H_{19}NO_5$ (m/z) $[M + H]^+$ calcd: 318.1263, found: 318.1254.

Dimethyl 4-(4-methoxyphenyl)-2,6-dimethyl-1,4-dihydropyridine-3,5-dicarboxylate (3n) Yellow solid; yield 87%; m.p. 181-183 °C; **^{1}H NMR** (500 MHz, DMSO-d_6): δ 9.11 (s, 1H, NH), 7.15 (d, 2H), .86 (d, 2H), 5.09 (s, 1H), 3.71 (s, 3H), 3.60 (s, 6H) 2.26 (s, 6H); **^{13}C NMR** (126 MHz, DMSO-d_6): δ 170.3, 159.3, 143.5, 130.7, 129.9, 113.2, 86.8, 56.7, 52.3, 33.2, 16.4; **HR-MS** (ESI) for $C_{18}H_{21}NO_5$ (m/z) $[M + H]^+$ calcd: 332.1420, found: 332.1417.

Dimethyl 4-(2-bromophenyl)-2,6-dimethyl-1,4-dihydropyridine-3,5-dicarboxylate (3o) Yellow solid; yield 90%; m.p. 205-207 °C; **^{1}H NMR** (500 MHz, DMSO-d_6): δ 9.53 (s, 1H), 7.72 (d, 1H), 7.59 (d, 1H), 7.28 (t, 1H), 7.16 (t, 1H), 5.18 (s, 1H), 3.72 (s, 6H), 2.22 (s, 6H) **^{13}C NMR** (126 MHz, DMSO-d_6): δ 171.4 143.5, 138.4, 133.2, 131.3, 128.9, 127.5, 126.6, 89.2, 51.3, 34.9, 16.2; **HR-MS** (ESI) for $C_{17}H_{18}BrNO_4$ (m/z) $[M + H]^+$ calcd: 380.0419, found: 380.0431.

Dimethyl 4-(4-chlorophenyl)-2,6-dimethyl-1,4-dihydropyridine-3,5-dicarboxylate (3p) Yellow solid; yield 91%; m.p. 198 °C; **^{1}H NMR** (500 MHz, DMSO-d_6): δ 9.34 (s, 1H), 8.22 (d, 2H), 8.22 (d, 2H), 7.50 (s, 1H), 5.27 (s, 1H), 4.00 (s, 6H), 2.31 (s, 6H) **^{13}C NMR** (126 MHz,

DMSO-d_6): δ 169.9 144.3, 139.8, 132.8, 131.8, 128.9, 88.4, 51.2, 34.4, 16.9; HR-MS (ESI) for $C_{17}H_{18}ClNO_4$ (m/z) $[M + H]^+$ calcd: 336.0924, found: 336.0915.

Dimethyl 4-(4-fluorophenyl)-2,6-dimethyl-1,4-dihydropyridine-3,5-dicarboxylate (3q) White solid; yield 93%; m.p. 173 °C; **^{1}H NMR** (500 MHz, DMSO-d_6): δ 9.61 (s, 1H), 8.24 (d, 2H), 8.06 (d, 2H), 5.43 (s, 1H), 4.02 (s, 6H), 2.32 (s, 6H) **^{13}C NMR** (126 MHz, DMSO-d_6): δ 172.2, 162.5 (d, JCF = 244.42 Hz, 1C), 143.3 (d, JCF = 2.52 Hz), 135.7, 128.4 (d, JCF = 8.7O Hz), 115.4 (d, JCF = 21.42 Hz), 88.5, 51.0, 35.1, 17.9; **^{19}F NMR** (471 MHz, $CDCl_3$): δ - 115.8; **HR-MS** (ESI) for $C_{17}H_{18}FNO_4$ (m/z) $[M + H]^+$ calcd: 320.1220, found: 320.1228.

Dimethyl 4-(2-hydroxy-3-methoxyphenyl)-2,6-dimethyl-1,4-dihydropyridine-3,5-dicarboxylate (3r) Yellowish solid; yield 93%; m.p. 195-197 °C; **^{1}H NMR** (500 MHz, DMSO-d_6): *δ* 9.28 (s, 1H), 7.41 (d, 1H), 7.32- 7.27 (m, 2H), 5.61 (s, 1H), 4.33 (s, 1H), 3.83 (s, 6H), 3.65 (s, 3H), 2.30 (s, 6H) **^{13}C NMR** (126 MHz, DMSO-d_6): δ 170.3 147.6, 146.1, 142.2, 140.5, 130.7, 124.0, 119.5, 116.3, 86.8, 56.2, 53.2, 28.4, 16.9; **HR-MS** (ESI) for $C_{18}H_{21}NO_6$ (m/z) $[M + H]^+$ calcd: 348.1369, found: 348.1354.

Dimethyl 2,6-dimethyl-4-(naphthalen-1-yl)-1,4-dihydropyridine-3,5-dicarboxylate (3s) Yellow solid; yield 93%; m.p. 204-206 °C; **^{1}H-NMR** (500 MHz, DMSO-d_6): δ 9.20 (s, 1H, NH), 8.07 (d, 1H), 7.86 (d, 1H,), 7.74, (d, 1H), 7.50 (t, 1H), 7.45 (t, 1H), 7.31 (t, 1H), 7.13 (d, 1H), 4.92 (s, 1H), 3.79 (s, 6H), 2.24 (s, 6H); **^{13}C-NMR** (126 MHz, DMSO-d_6): δ 170.2, 140.4, 135.1, 132.8, 131.2, 130.6, 129.9, 129.3, 128.4, 127.6, 126.7, 124.0, 86.8, 56.2, 34.0, 16.9; **HR-MS** (ESI) for $C_{21}H_{21}NO_4$ (m/z) $[M + H]^+$ calcd: 352.1470, found: 352.1475.

Dimethyl 2,6-dimethyl-4-(thiophen-2-yl)-1,4-dihydropyridine-3,5-dicarboxylate (3t) Brown solid; yield 89%; m.p. 212-214; °C; **^{1}H NMR** (500 MHz, DMSO-d_6): δ 9.34 (s, 1H, NH), 7.16 (d,

1H), 6.91 (t, 1H), 6.82 (d, 1H), 4.60 (s, 1H), 4.84 (s, 6H), 2.37 (s, 6H); **^{13}C NMR** (126 MHz, DMSO-d_6): δ 170.0, 153.0, 141.9, 135.5, 126.6, 125.0, 89.2, 52.0, 37.4, 16.1; **HR-MS** (ESI) for $C_{15}H_{17}NO_4S$ (m/z) [M + H]$^+$ calcd: 308.0878, found: 308.0875.

Dimethyl 2',6'-dimethyl-1',4'-dihydro-[2,4'-bipyridine]-3',5'-dicarboxylate (3u) Brown solid; yield 89%; m.p. 248-250 °C; **^{1}H NMR** (500 MHz, DMSO-d_6): δ 8.57 (d, 2H), 9.20 (s, 1H, NH), 8.62 (d, 1H), 7.63 (t, 1H), 7.28 (t, 1H), 7.16 (d, 1H) 4.72 (s, 1H), 3.79 (s, 6H), 2.25 (s, 6H); **^{13}C NMR** (126 MHz, DMSO-d_6): δ 170.2, 157.3, 146.2, 142.2, 140.5, 129.9, 119.5, 91.7, 53.2, 35.9, 16.9; **HR-MS** (ESI) for $C_{16}H_{18}N_2O_4$ (m/z) [M + H]$^+$ calcd:303.1266, found: 303.1254.

9-phenyl-3,4,6,7,9,10-hexahydroacridine-1,8(2H,5H)-dione (3v) Yellowish solid; yield 93%; m.p. 281 °C; **^{1}H NMR** (500 MHz, DMSO-d_6): δ 9.88 (s, 1H, NH), 7.47 (d, 2H), 7.34 (t, 3H), 4.96 (s, 1H), 2.45 (t, 4H), 2.33 (t, 4H), 1.63-1.59 (m, 4H); **^{13}C NMR** (126 MHz, DMSO-d_6): δ 194.1, 149.0, 140.4, 129.2, 128.9, 127.7, 126.6, 106.2, 37.3, 32.6, 29.0, 20.8; **HR-MS** (ESI) for $C_{19}H_{19}NO_2$ (m/z) [M + H]$^+$ calcd: 294.1416, found: 294.1424.

9-(4-methoxyphenyl)-3,4,6,7,9,10-hexahydroacridine-1,8(2H,5H)-dione (3w) Yellow solid; yield 92%; m.p. 190-192 °C; **^{1}H NMR** (500 MHz, DMSO-d_6): 9.88 (s, 1H, NH), 7.50 (d, 2H), 7.15 (d, 2H), 4.96 (s, 1H), 3.76 (s, 3H), 2.63 (t, 4H), 2.32 (t, 4H), 1.64-1.60 (m, 4H); **^{13}C NMR** (126 MHz, DMSO-d_6): δ 193.4, 161.5, 149.1, 140.4, 130.8, 129.9, 113.0, 106.2, 57.2, 37.3, 32.6, 29.0, 20.9; **HR-MS** (ESI) for $C_{20}H_{21}NO_3$ (m/z) [M + H]$^+$ calcd: 324.1521, found: 324.1518.

9-(3-chlorophenyl)-3,4,6,7,9,10-hexahydroacridine-1,8(2H,5H)-dione (3x) Light yellow solid; yield 90%; m.p. 202-204 °C; **^{1}H NMR** (500 MHz, DMSO-d_6): *δ* 9.83 (s, 1H, NH), 7.71 (s, 1H), 7.42 (d, 1H), 7.35 (d, 1H), 7.21 (t, 1H), 4.87 (s, 1H), 2.31 (t, 4H), 2.19 (t, 4H), 1.62-1.58 (m, 4H); **^{13}C NMR** (126 MHz, DMSO-d_6): δ 195.7, 150.5, 142.2, 139.9, 129.4, 128.5, 127.7,

126.5, 105.2, 38.2, 33.3, 28.4, 21.0; **HR-MS** (ESI) for $C_{19}H_{18}ClNO_2$ (m/z) $[M + H]^+$ calcd: 328.1026, found: 328.1022.

9-(4-bromophenyl)-3,4,6,7,9,10-hexahydroacridine-1,8(2H,5H)-dione (3y) Yellow solid; yield 93%; m.p. 295 °C; **^{1}H NMR** (500 MHz, DMSO-*d$_6$*): *δ* 9.66 (s, 1H, NH), 7.51 (d, 2H), 7.37 (d, 2H), 4.97 (s, 1H), 2.33 (t, 4H) 2.25 (t, 4H), 1.60-1.60 (m, 4H); **^{13}C NMR** (126 MHz, DMSO-*d$_6$*): δ 195.7, 150.5, 138.9, 131.4, 129.5, 124.2, 105.4, 38.1, 33.4, 28.4, 21.9; **HR-MS** (ESI) for $C_{19}H_{18}BrNO_2$ (m/z) $[M + H]^+$ calcd: 372.0521, found: 372.0511.

3,3,6,6-tetramethyl-9-phenyl-3,4,6,7,9,10-hexahydroacridine-1,8(2H,5H)-dione(3z) Yellow solid; yield 92%; m.p. 277 °C; **^{1}H NMR** (500 MHz, DMSO-*d$_6$*): δ 9.23 (s, 1H, NH), 7.49 (d, 2H), 7.36 (t, 1H), 7.25 (d, 2H), 4.78 (s, 1H), 2.61 (s, 4H), 2.24 (s, 4H), 1.19 (s, 12H); **^{13}C NMR** (126 MHz, DMSO-*d$_6$*): *δ* 194.5, 159.9, 140.7, 128.9, 128.5, 127.5, 112.6, 51.9, 32.8, 32.3, 28.3; **HR-MS** (ESI) for $C_{23}H_{27}NO_2$ (m/z) $[M + H]^+$ calcd: 350.2042, found: 350.2063.

9-(4-methoxyphenyl)-3,3,6,6-tetramethyl-3,4,6,7,9,10-hexahydroacridine-1,8(2H,5H)-dione (3aa) Brown solid; yield 92%; m.p. 268-270 °C; **^{1}H NMR** (500 MHz, DMSO-*d$_6$*): *δ* 9.25 (s, 1H, NH), 7.52 (d, 2H), 7.03 (d, 2H), 4.68 (s, 1H), 3.85 (s, 3H), 2.42 (s, 4H), 2.08 (s, 4H), 1.18 (s, 12H); **^{13}C NMR** (126 MHz, DMSO-*d$_6$*): δ 194.6, 160.2, 159.8, 130.5, 129.4, 113.3, 112.2, 55.2, 51.2, 36.4, 32.8, 32.4; **HR-MS** (ESI) for $C_{24}H_{29}NO_3$ (m/z) $[M + H]^+$ calcd: 380.2147 found: 380.2141.

9-(3-chlorophenyl)-3,3,6,6-tetramethyl-3,4,6,7,9,10-hexahydroacridine-1,8(2H,5H)-dione (3ab) Yellow solid; yield 90%; m.p. 295-297 °C; **^{1}H NMR** (500 MHz, DMSO-*d$_6$*): *δ* 9.33 (s, 1H, NH), 7.63 (s, 1H), 7.51 (dt, 2H), 7.19 (d, 1H), 4.80 (s, 1H), 2.42 (s, 4H), 2.08 (s, 4H), 1.18 (s, 12H); **^{13}C NMR** (126 MHz, DMSO-*d$_6$*): δ 194.5, 159.8, 143.9, 134.0, 128.9, 128.6, 127.5,

126.6, 112.7, 51.9, 36.4, 32.8, 32.3, 28.4; **HR-MS** (ESI) for $C_{23}H_{26}ClNO_2$ (m/z) $[M + H]^+$ calcd: 384.1652, found: 384.1643.

3,3,6,6-tetramethyl-9-(pyridin-2-yl)-3,4,6,7,9,10-hexahydroacridine-1,8(2H,5H)-dione (3ac) Light orange solid; yield 88%; m.p. 298 °C; **^{1}H NMR** (500 MHz, DMSO-d_6): δ 9.25 (s, 1H, NH), 8.68 (t, 1H), 7.70 (t, 1H), 7.26 (t, 1H), 7.19 (d, 1H), 4.73 (s, 1H), 2.63 (s, 4H), 2.08 (s, 4H), 1.09 (s, 12H); **^{13}C NMR** (126 MHz, DMSO-d_6): δ 193.4, 157.4, 156.3, 145.7, 141.0, 129.5, 119.0, 112.2, 51.7, 34.2, 33.0, 28.8; **HR-MS** (ESI) for $C_{22}H_{26}N_2O_2$ (m/z) $[M + H]^+$ calcd: 351.1994, found: 351.1998.

2,2,8,8-tetramethyl-5-phenyl-5,10-dihydro-4H,6H-bis([1,3]dioxino)[4,5-b:5',4' e]pyridine-4,6-dione (3ad) White solid; yield 92%; m.p. 172-174 °C; **^{1}H NMR** (500 MHz, DMSO-d_6): δ 9.43 (s, 1H, NH), 7.52 (d, 2H), 7.38 (t, 3H), 4.80 (s, 1H), 1.87 (s, 12H); **^{13}C NMR** (126 MHz, DMSO-d_6): δ 165.3, 149.4, 141.0, 128.6, 128.9, 128.6, 127.5, 100.2, 85.2, 38.4, 28.7; **HR-MS** (ESI) for $C_{19}H_{19}NO_6$ (m/z) $[M + H]^+$ calcd: 358.1212, found: 3581225.

5-(4-methoxyphenyl)-2,2,8,8-tetramethyl-5,10-dihydro-4H,6H-bis([1,3]dioxino) [4,5-b:5',4' e] pyridine-4,6-dione (3ae) Yellow solid; yield 92%; m.p. 171 °C; **^{1}H NMR** (500 MHz, DMSO-d_6): δ 9.15 (s, 1H, NH), 7.42 (d, 2H), 6.97 (d, 2H), 4.64 (s, 1H), 3.84 (s, 3H), 1.86 (s, 12H); **^{13}C NMR** (126 MHz, DMSO-d_6): δ 165.0, 159.9, 150.0, 130.5, 129.4, 113.8, 100.2, 85.2, 56.3, 38.4, 26.3; **HR-MS** (ESI) for $C_{20}H_{21}NO_7$ (m/z) $[M + H]^+$ calcd: 388.1318, found: 388.1328.

5-(4-bromophenyl)-2,2,8,8-tetramethyl-5,10-dihydro-4H,6H-bis([1,3]dioxino)[4,5-b:5',4'-e]pyridine-4,6-dione (3af) Yellow solid; yield 91%; m.p. 187 ^{o}C; **^{1}H NMR** (500 MHz, DMSO-d_6): δ 9.34 (s, 1H, NH), 7.43 (d, 2H), 7.28 (d, 2H), 4.72 (s, 1H), 1.76 (s, 12H); **^{13}C NMR** (126

MHz, DMSO-d_6): δ 165.1, 149.9, 138.0, 130.6, 129.4, 124.5, 100.2, 85.2, 38.4, 26.4; **HR-MS** (ESI) for $C_{19}H_{18}BrNO_6$ (m/z) $[M + H]^+$ calcd: 436.0317, found: 436.0325.

dimethyl 2,6-dimethyl-4-(2-nitrophenyl)-1,4-dihydropyridine-3,5-dicarboxylate (3ag)

Yellow solid; yield 86%; m.p. 172 ^oC; ^{1}**H NMR** (500 MHz, DMSO-d_6): δ 9.02 (s, 1H, NH), 7.71 (d, 1H), 7.60 (t, 1H), 7.46 (d, 1H), 7.35 (t, 1H), 5.50 (s, 1H), 3.46 (s, 6H), 2.26 (s, 6H); ^{13}**C NMR** (126 MHz, DMSO-d_6): δ 167.4, 147.7, 146.9, 142.4, 133.8, 131.1, 127.9, 124.2, 101.8, 51.0, 34.3, 18.7; **HR-MS** (ESI) for $C_{17}H_{18}N2O_6$ (m/z) $[M + H]^+$ calcd: 347.1165, found: 347.1268.

6. ^{1}H, ^{13}C, ^{19}F NMR & Mass spectra of products [3a-3ag]

^{1}H and ^{13}C NMR Spectra of Diethyl 2,6-dimethyl-4-phenyl-1,4-dihydropyridine-3,5-dicarboxylate (3a)

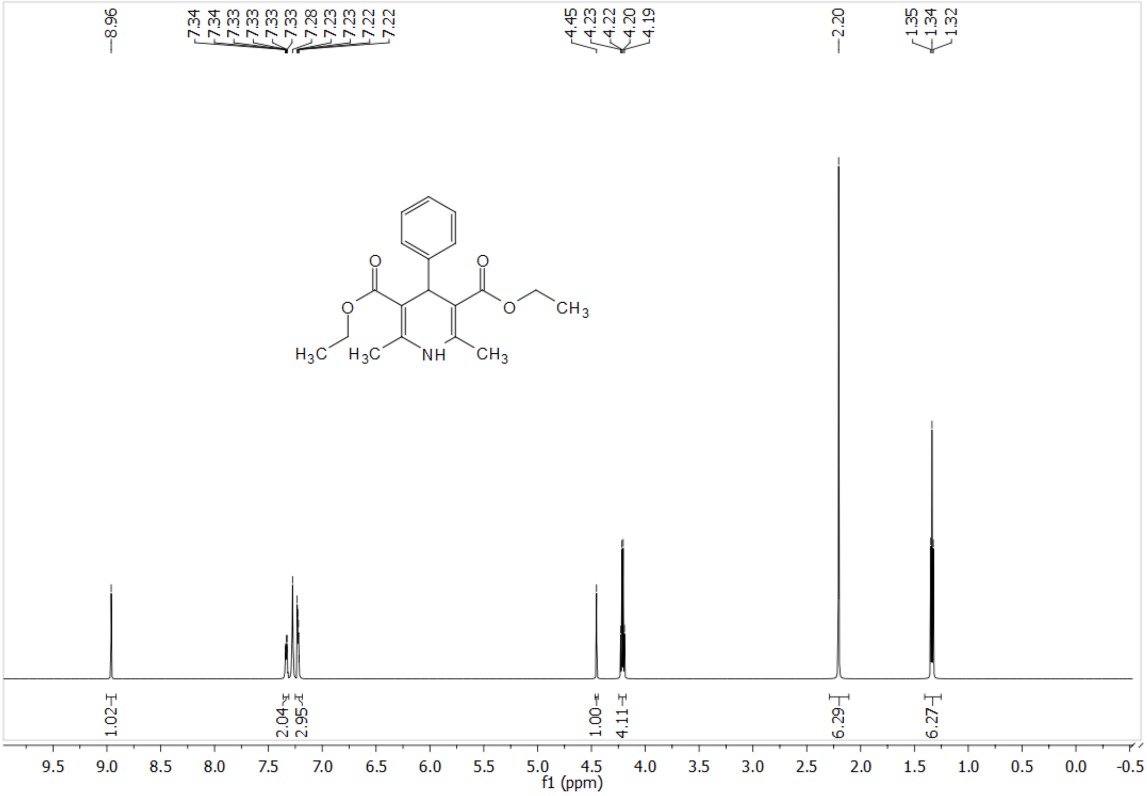

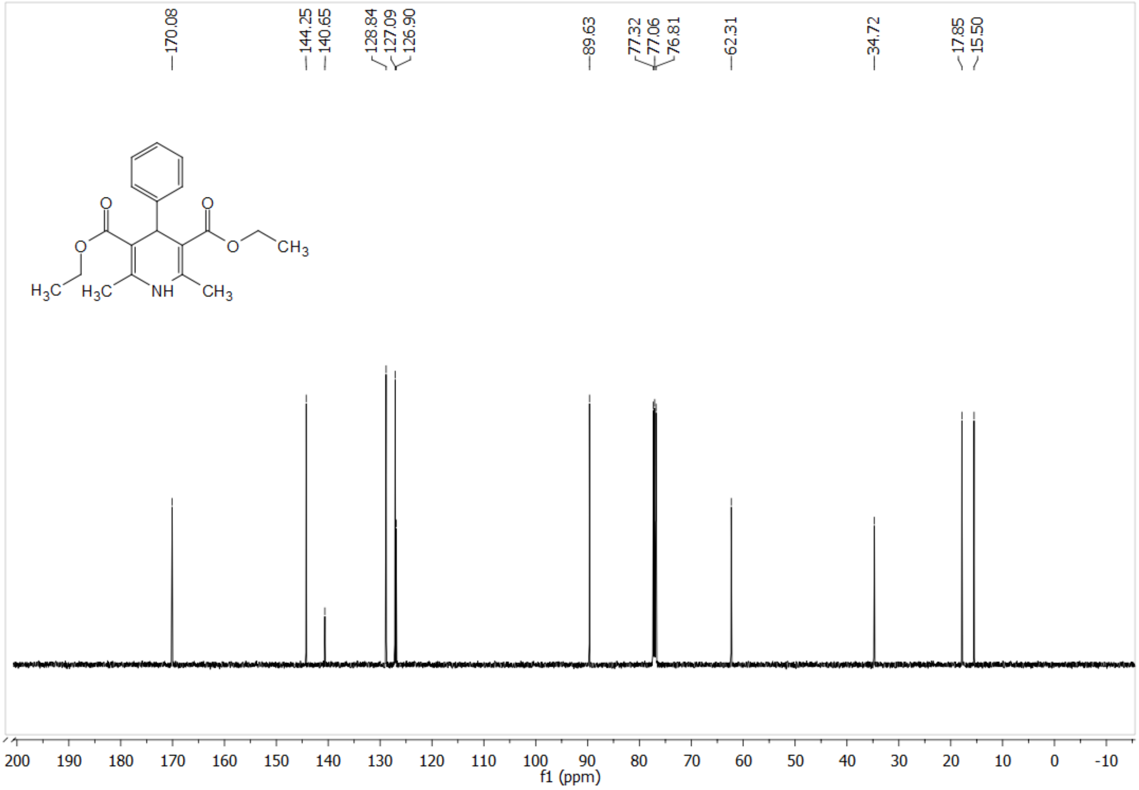

^{1}H and ^{13}C NMR Spectra of Diethyl 4-(2-methoxyphenyl)-2,6-dimethyl-1,4-dihydropyridine-3,5-dicarboxylate (3b)

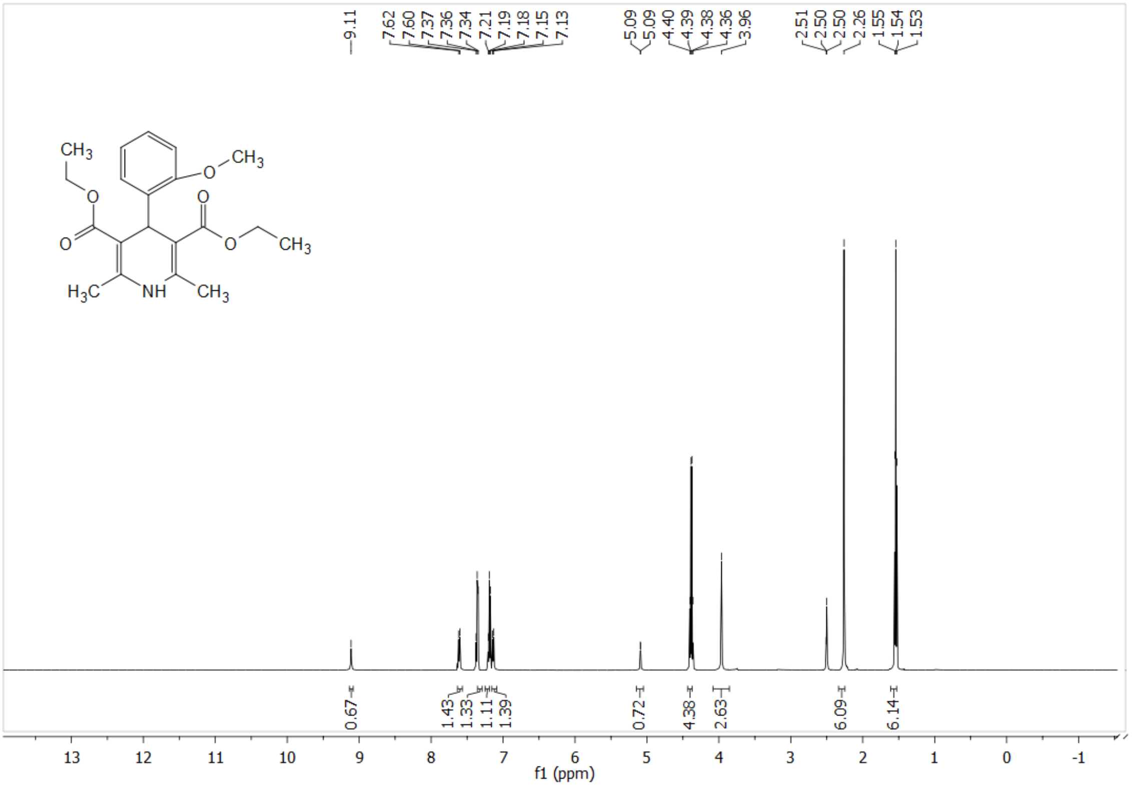

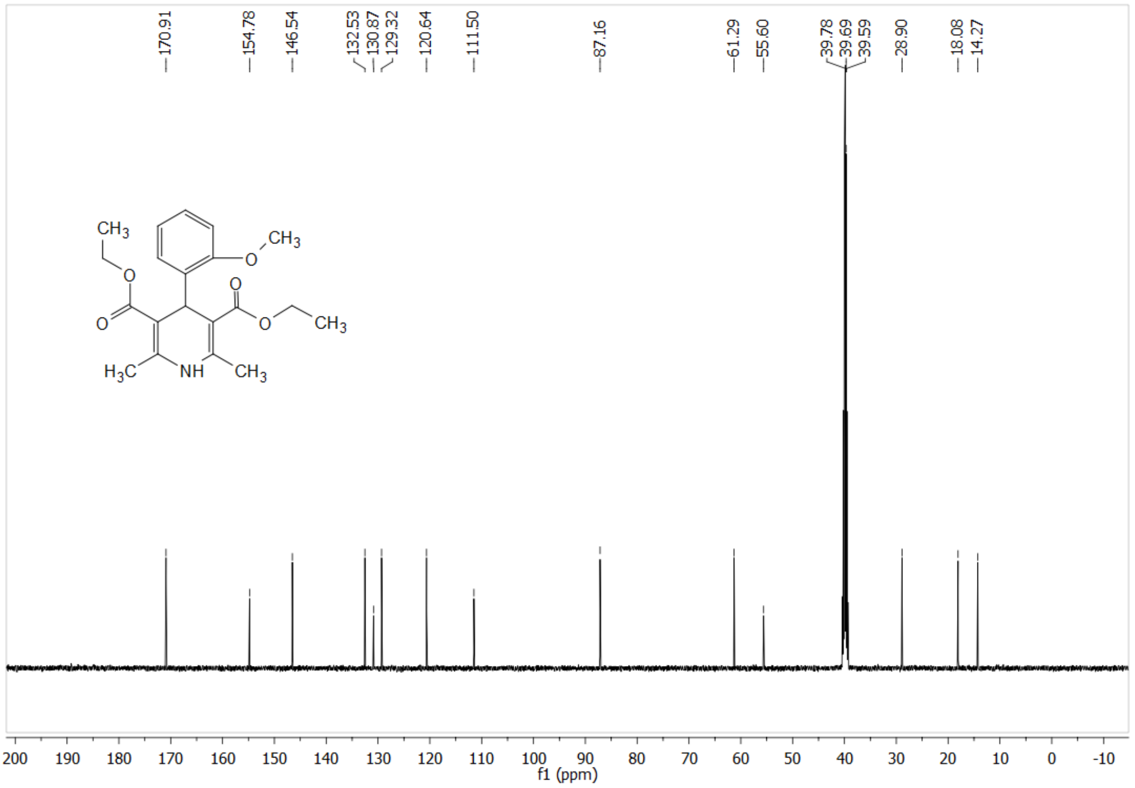

^{1}H and ^{13}C NMR Spectra of Diethyl 4-(3-hydroxyphenyl)-2,6-dimethyl-1,4-dihydropyridine-3,5-dicarboxylate (3c)

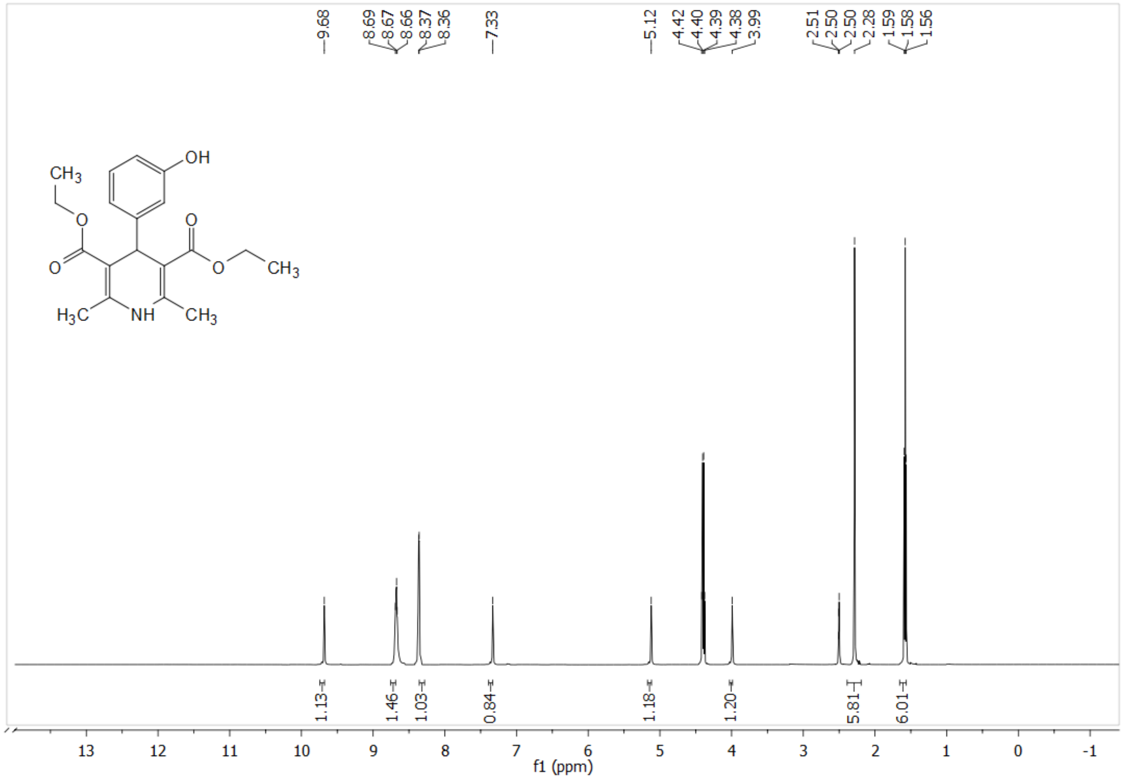

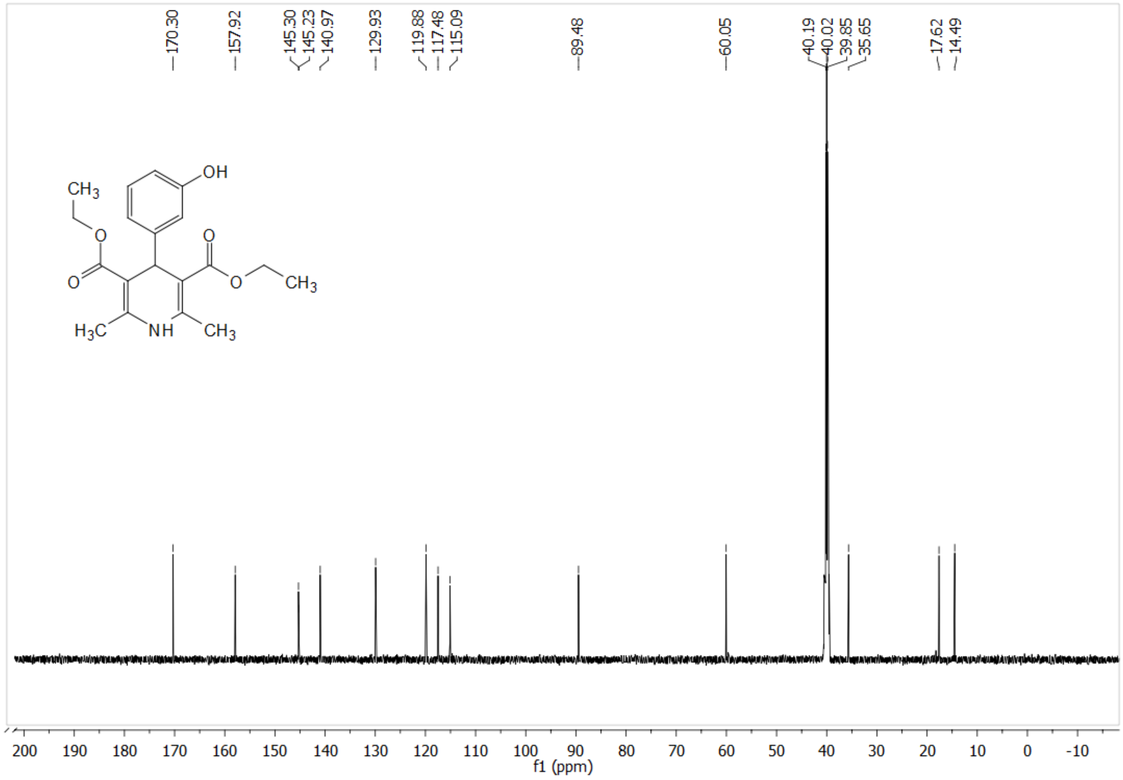

^{1}H and ^{13}C NMR Spectra of Diethyl 4-(4-methoxyphenyl)-2,6-dimethyl-1,4-dihydropyridine-3,5-dicarboxylate (3d)

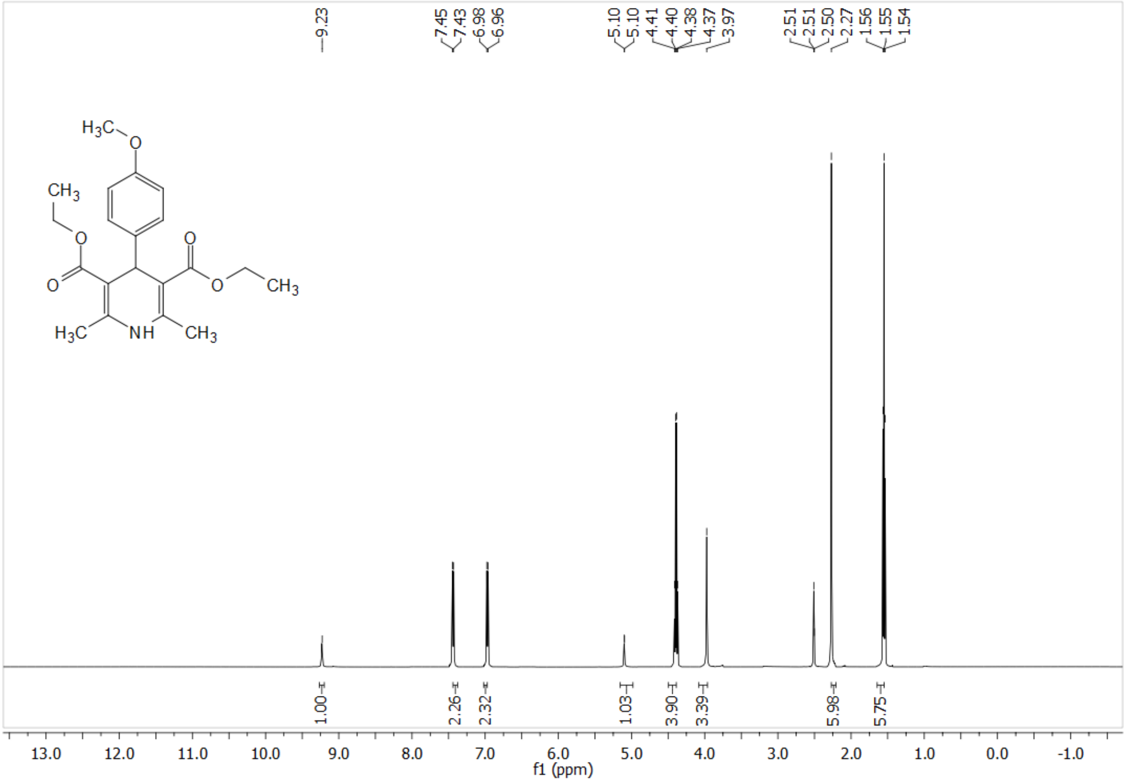

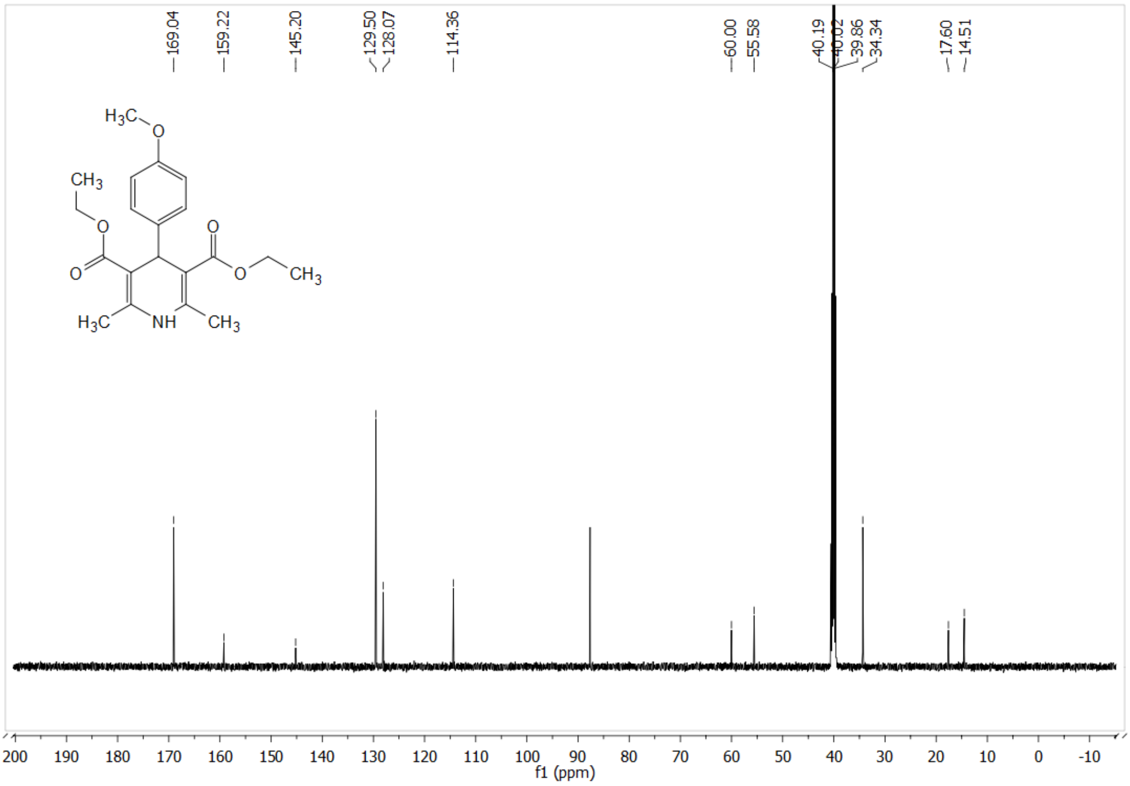

^{1}H and ^{13}C NMR Spectra of Diethyl 4-(2-chlorophenyl)-2,6-dimethyl-1,4-dihydropyridine-3,5-dicarboxylate (3e)

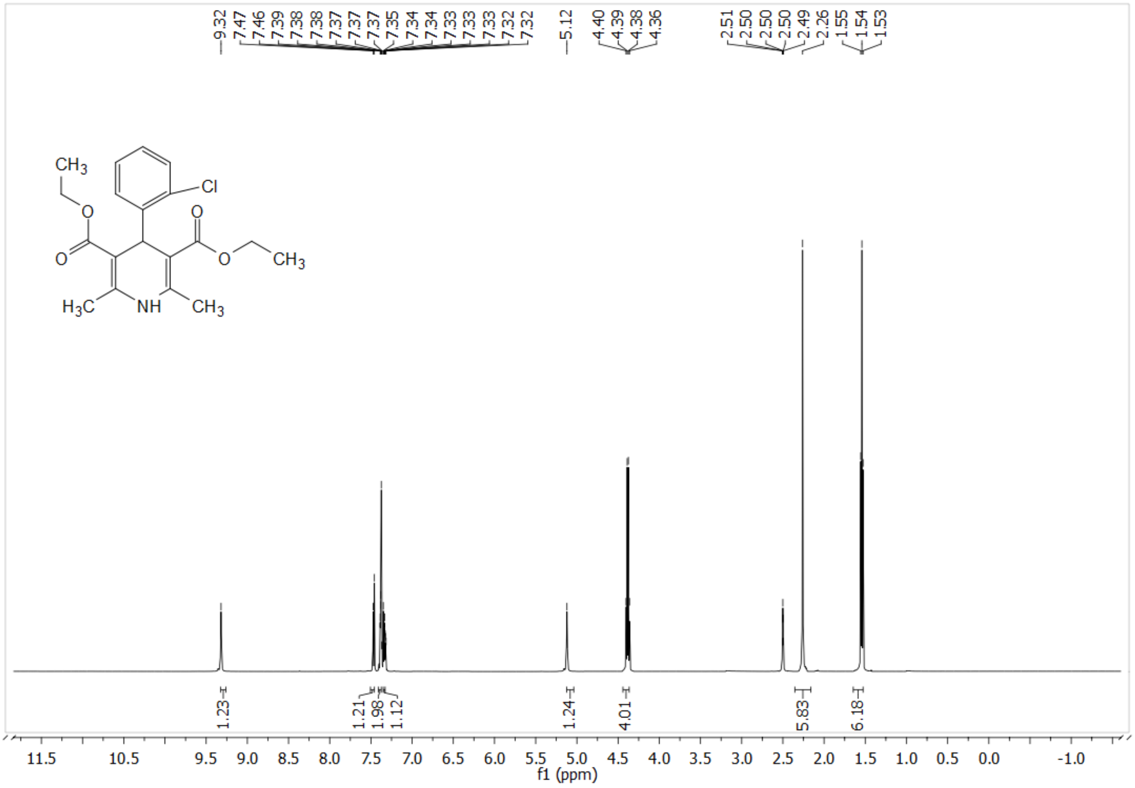

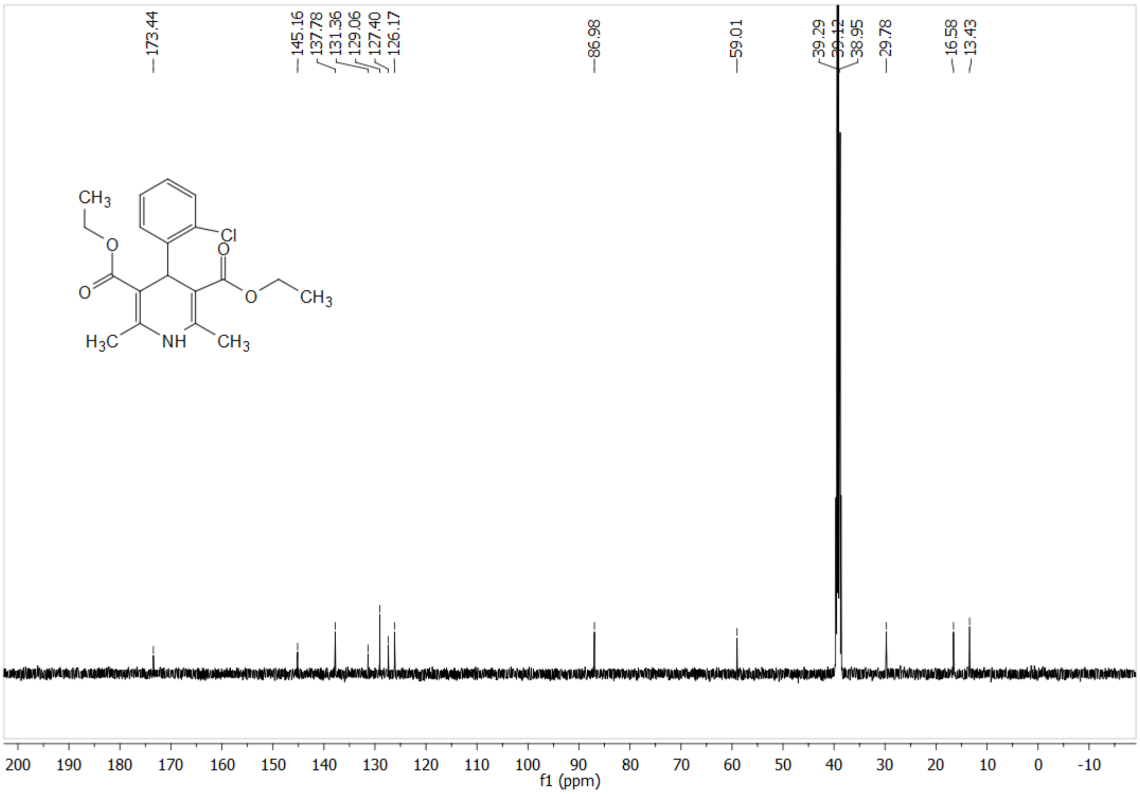

^{1}H and ^{13}C NMR Spectra of Diethyl 2,6-dimethyl-4-(2-nitrophenyl)-1,4-dihydropyridine-3,5-dicarboxylate (3f)

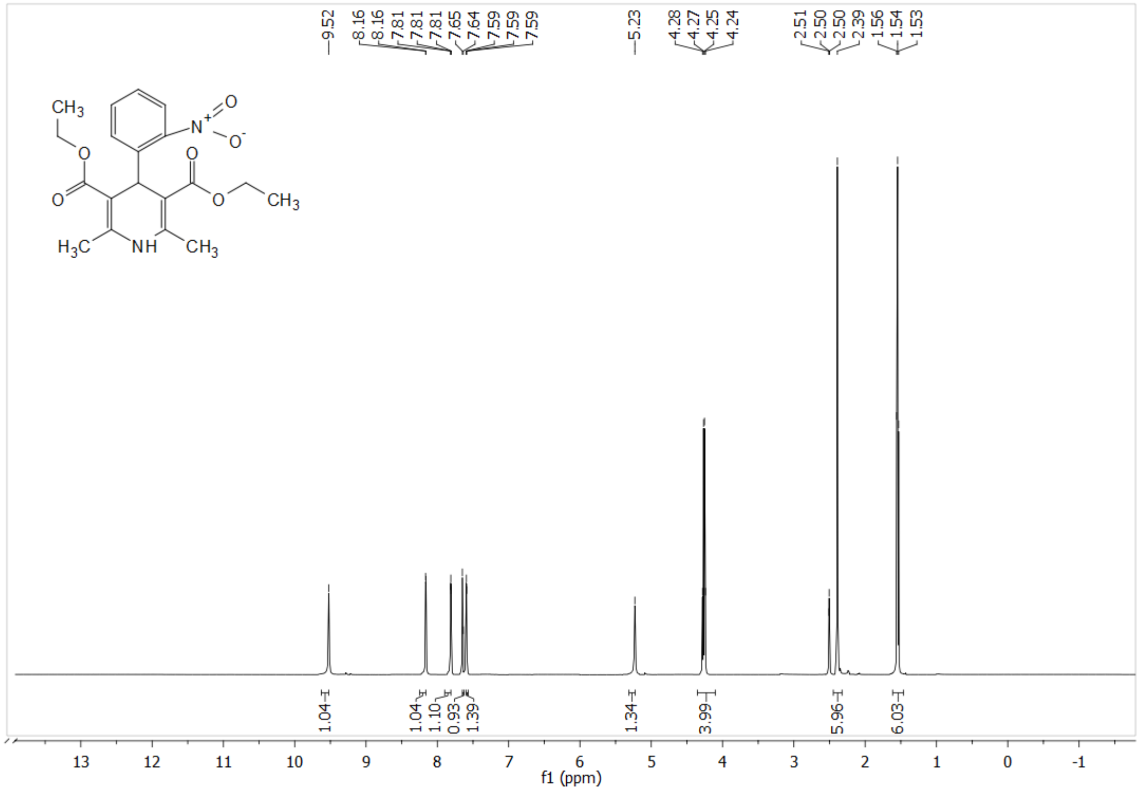

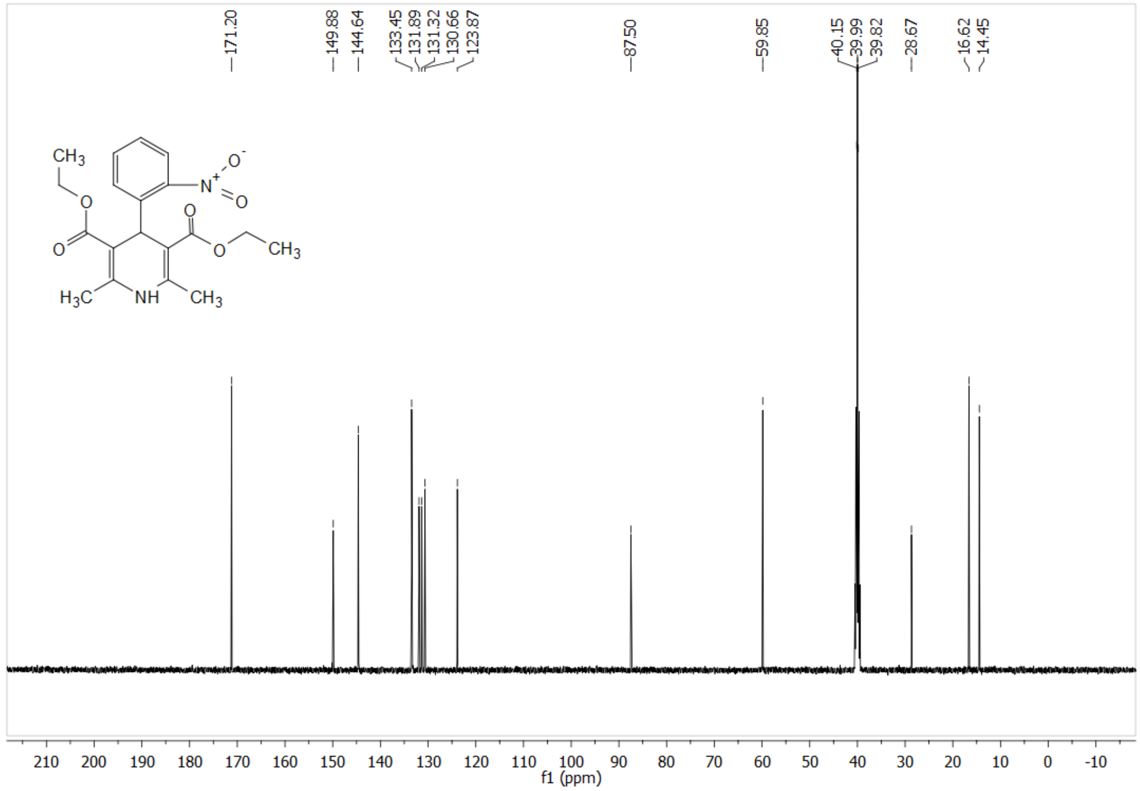

^{1}H and ^{13}C NMR Spectra of Diethyl 4-(4-bromophenyl)-2,6-dimethyl-1,4-dihydropyridine-3,5-dicarboxylate (3g)

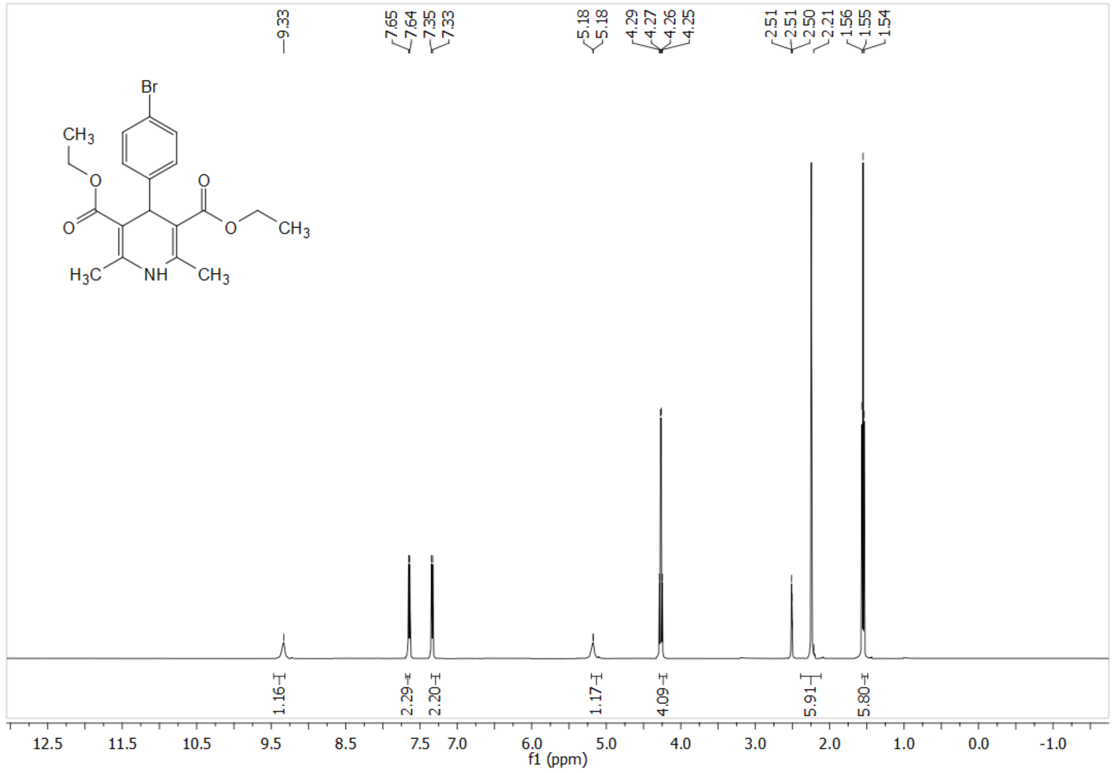

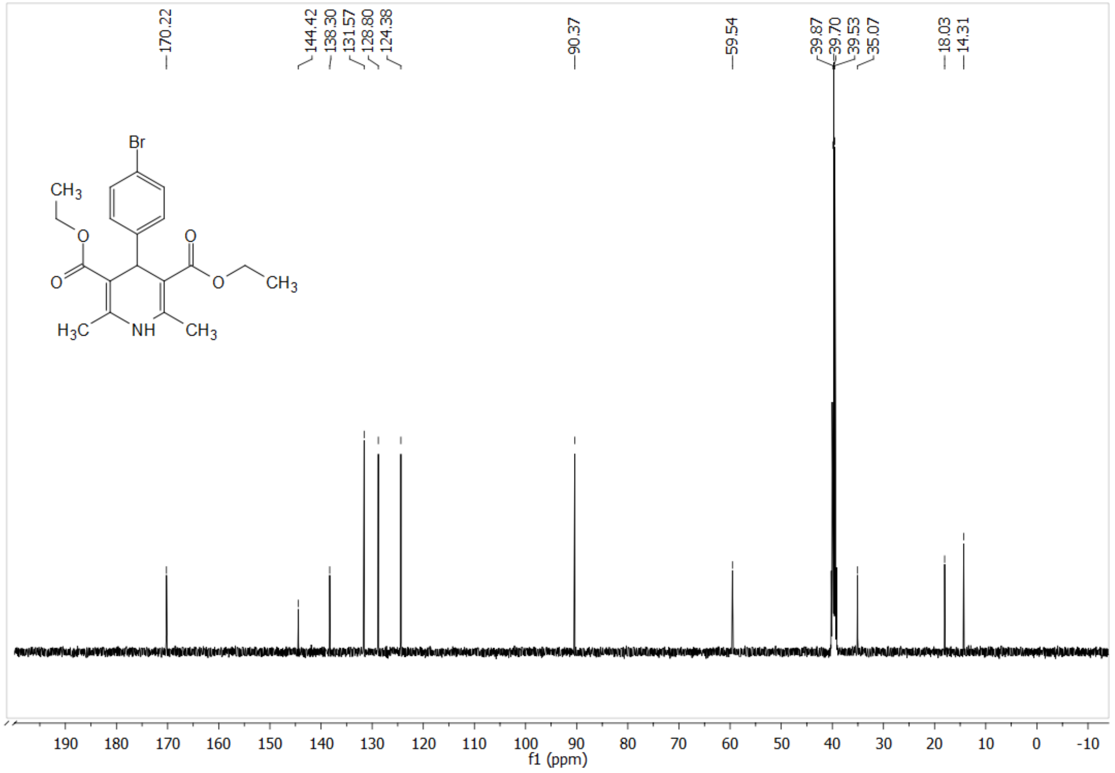

^{1}H and ^{13}C NMR Spectra of Diethyl 2,6-dimethyl-4-(4-nitrophenyl)-1,4-dihydropyridine-3,5-dicarboxylate (3h)

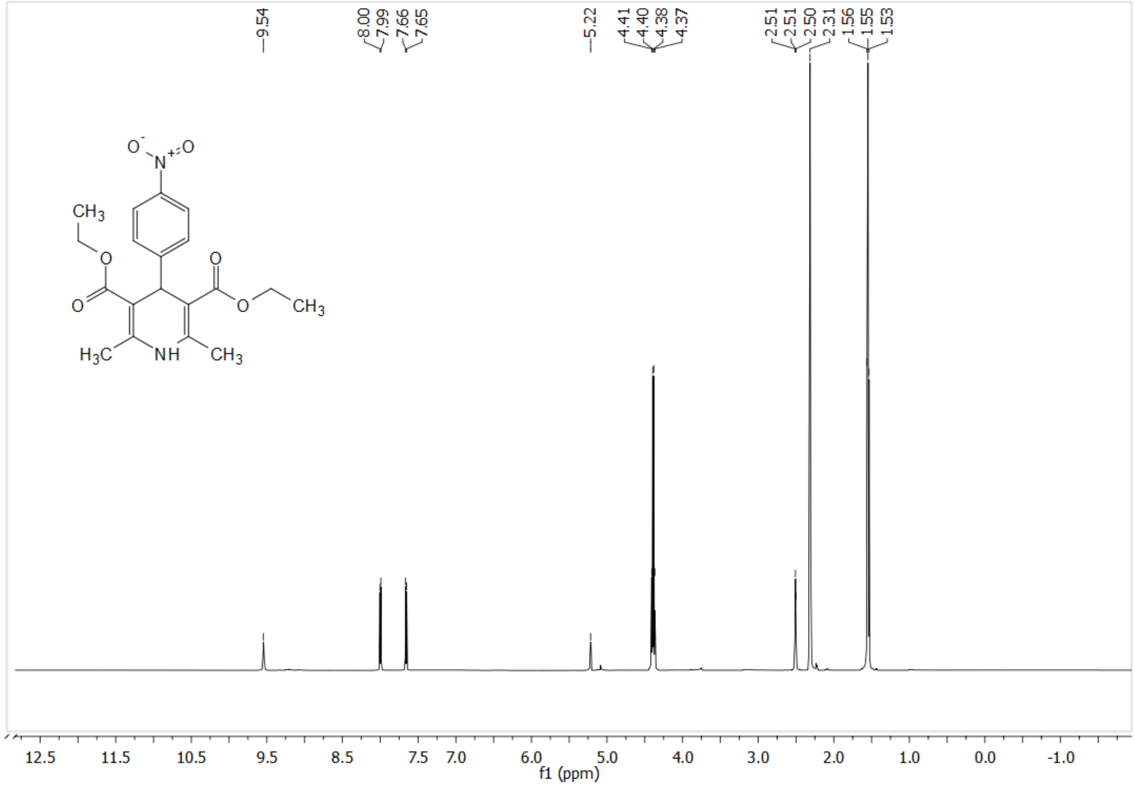

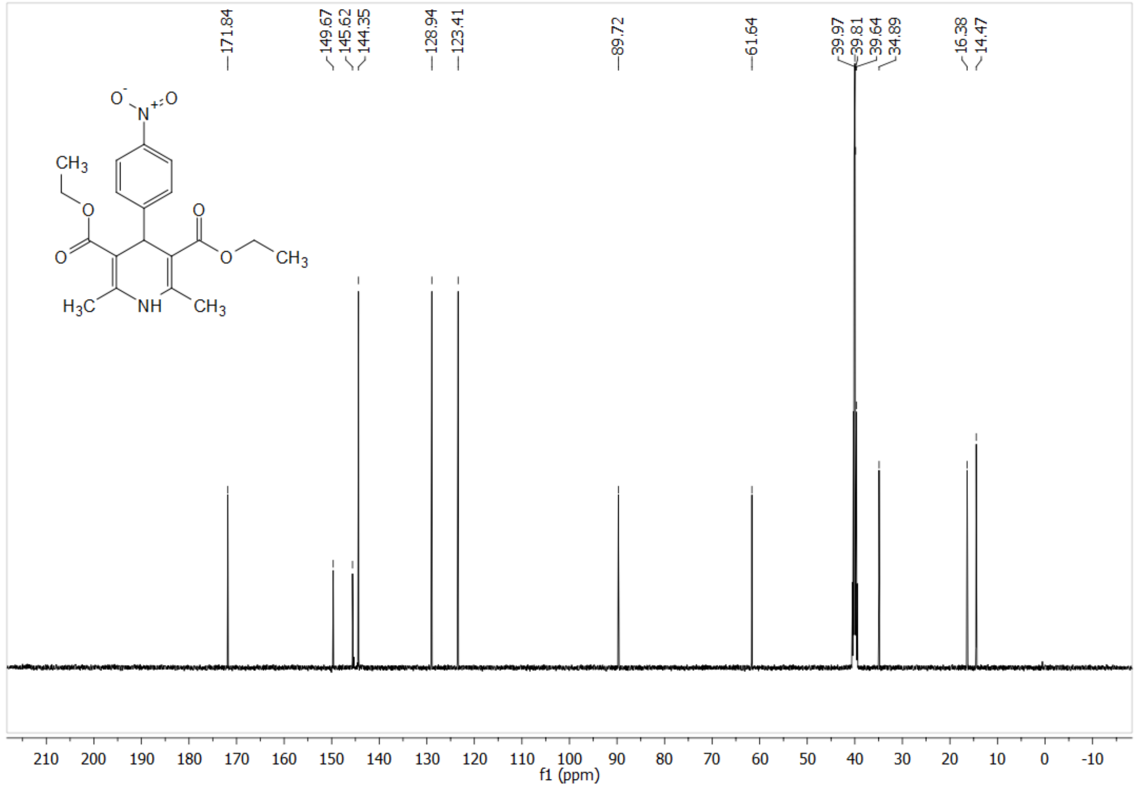

^{1}H and ^{13}C NMR Spectra of Diethyl 2',6'-dimethyl-1',4'-dihydro-[2,4'-bipyridine]-3',5'-dicarboxylate (3i)

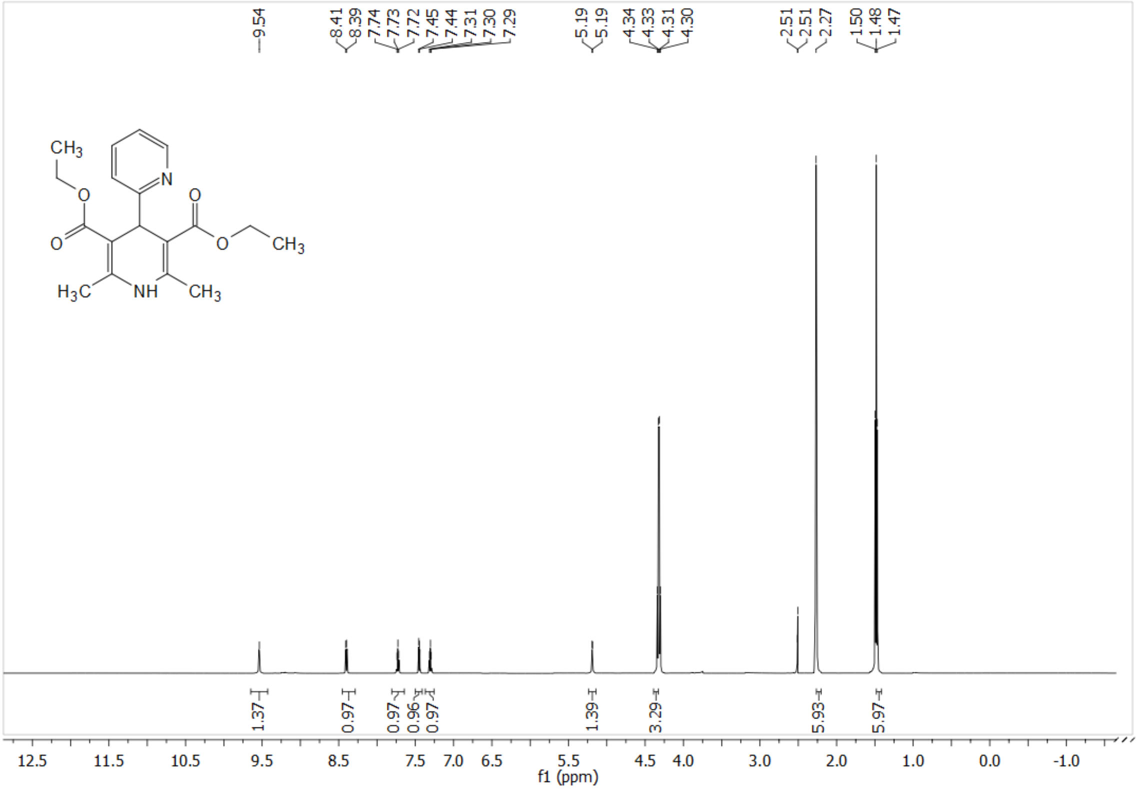

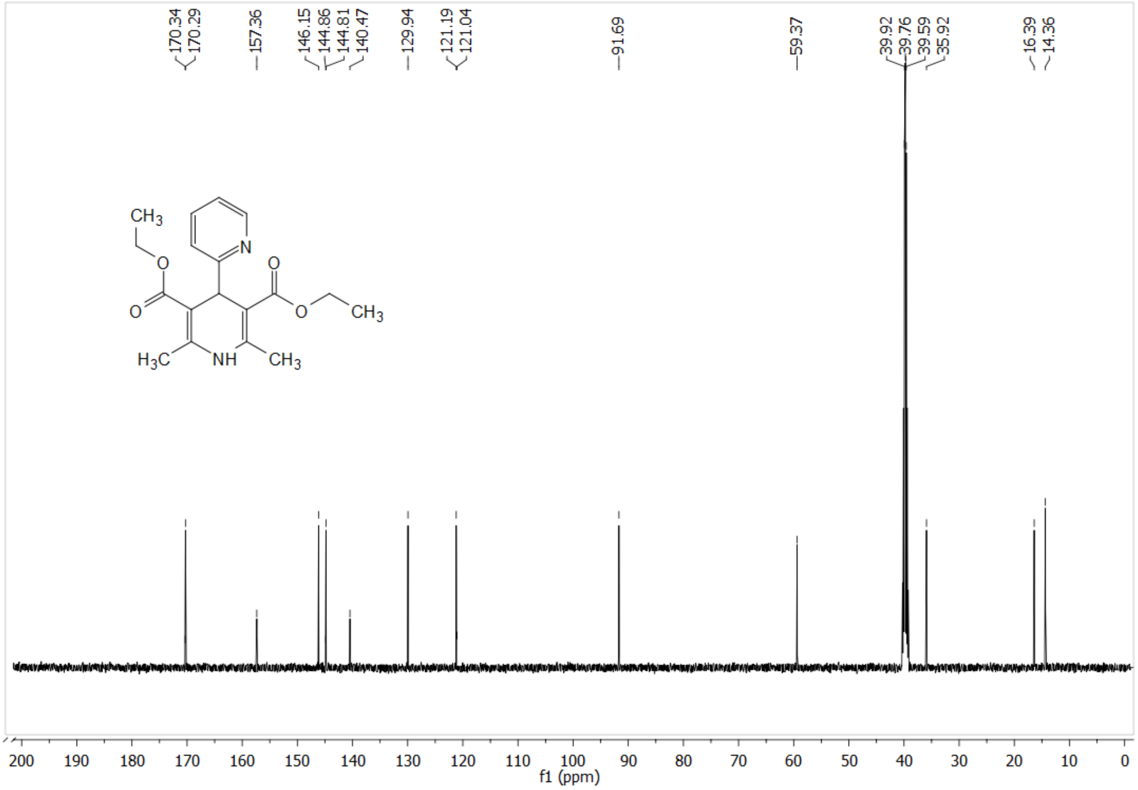

^{1}H and ^{13}C NMR Spectra of Diethyl 4-(furan-2-yl)-2,6-dimethyl-1,4-dihydropyridine-3,5-dicarboxylate (3j)

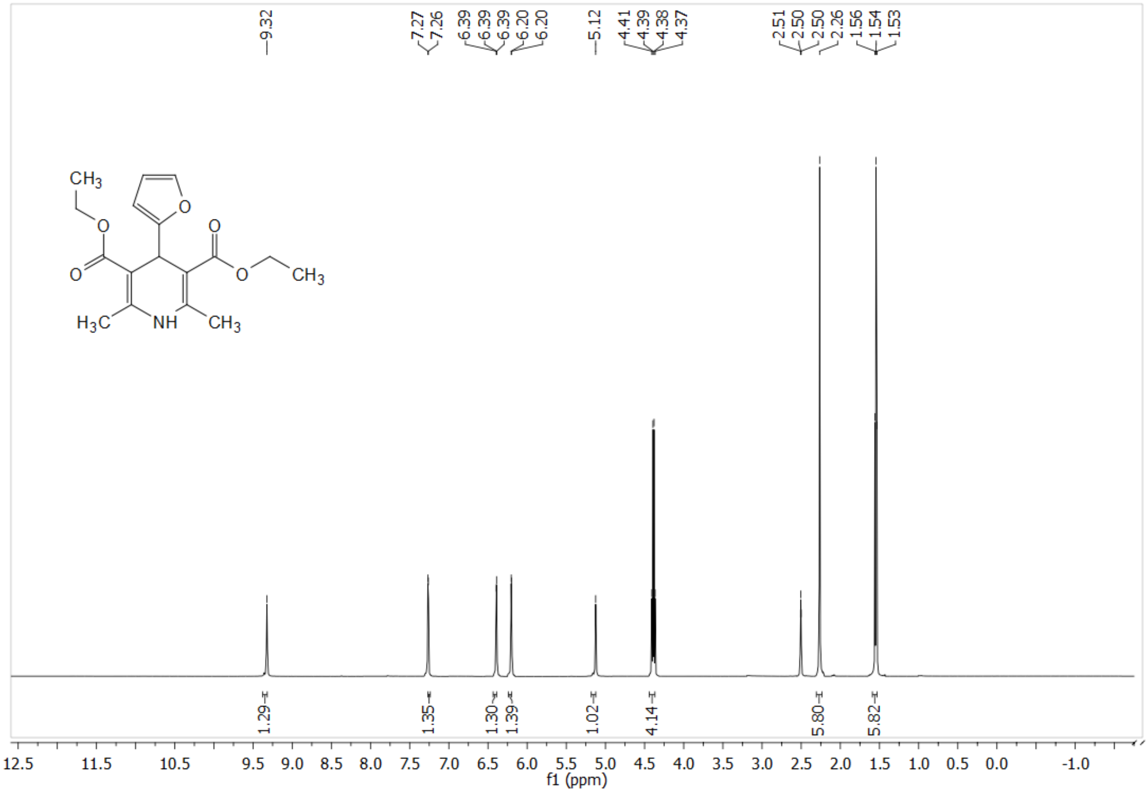

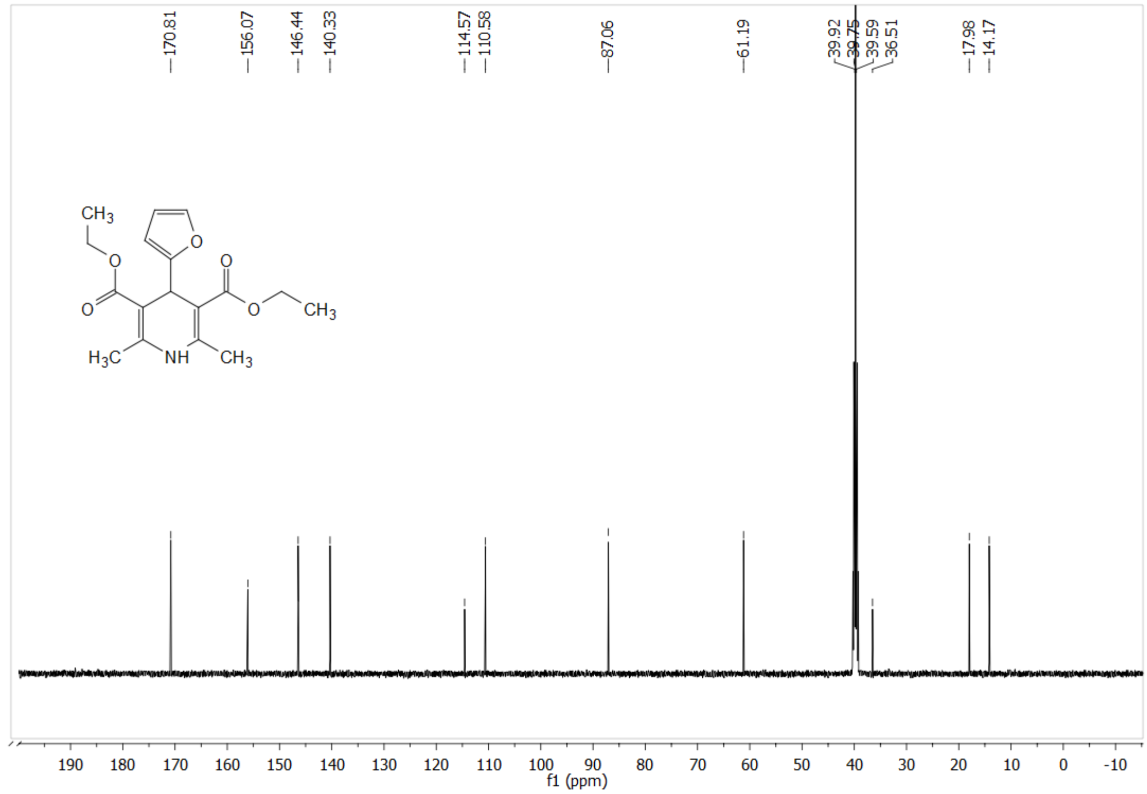

^{1}H and ^{13}C NMR Spectra of Diethyl 2,6-dimethyl-4-(thiophen-2-yl)-1,4-dihydropyridine-3,5-dicarboxylate (3k)

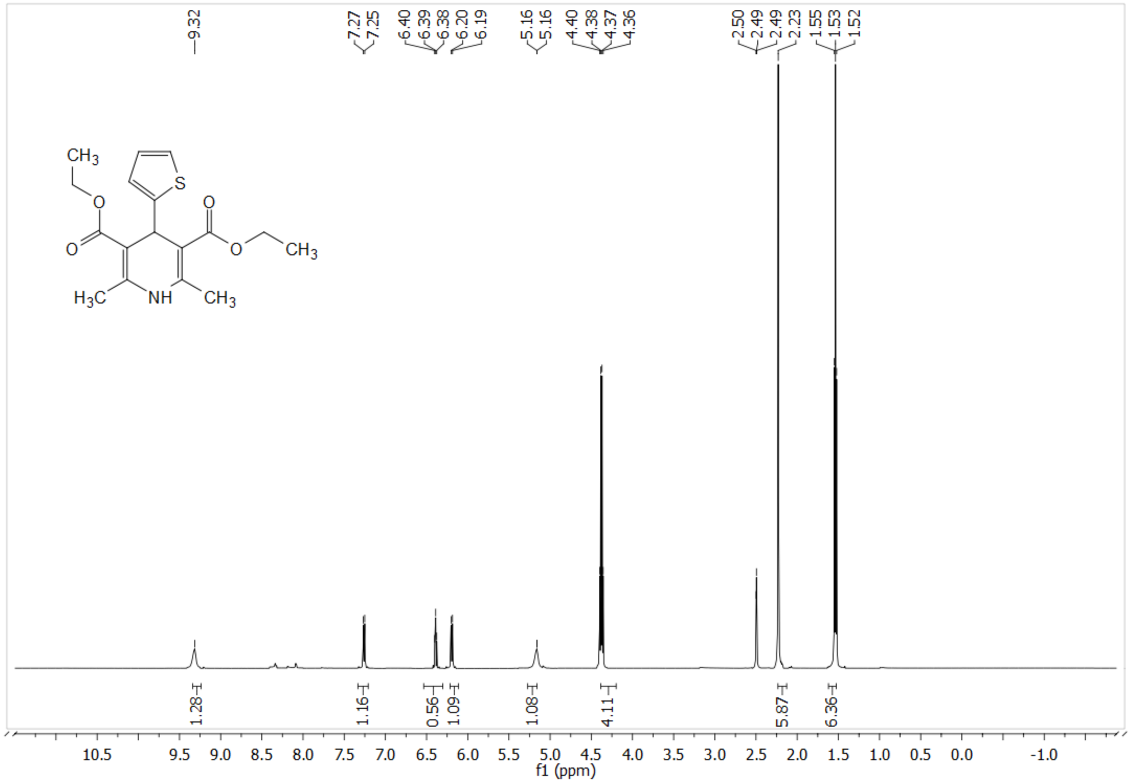

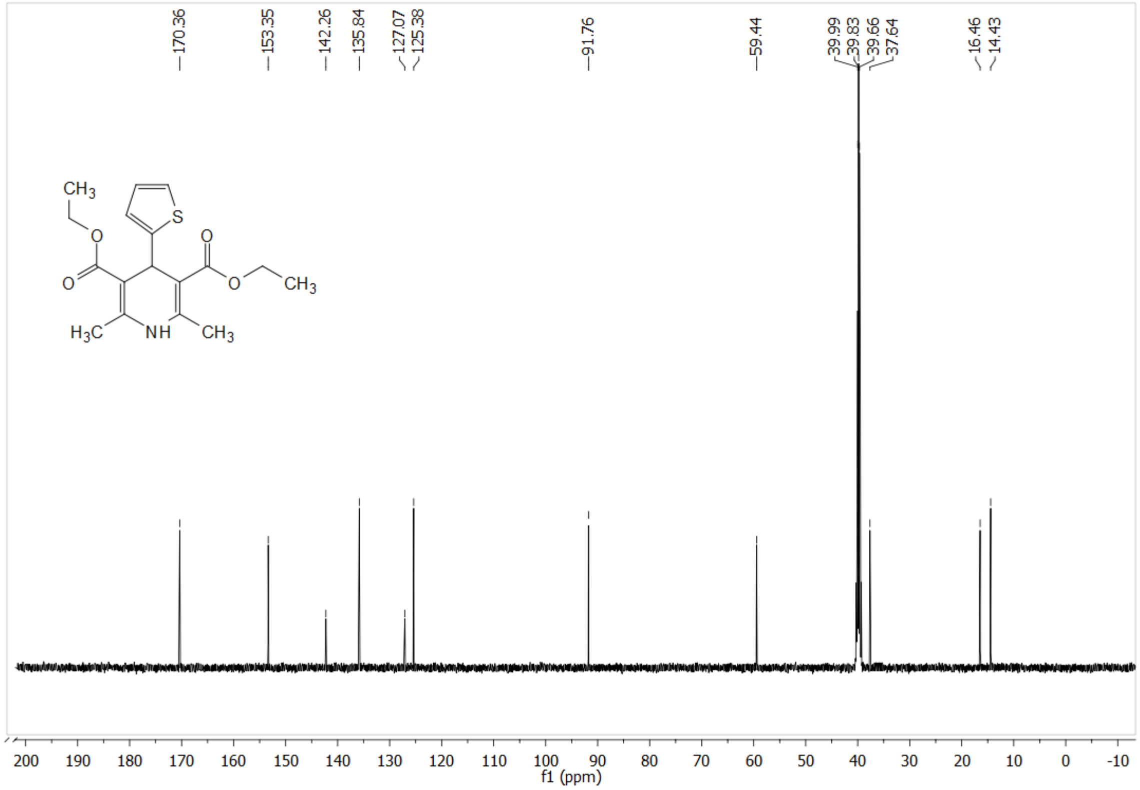

^1H and ^{13}C NMR Spectra of Dimethyl 2,6-dimethyl-4-phenyl-1,4-dihydropyridine-3,5-dicarboxylate (3l)

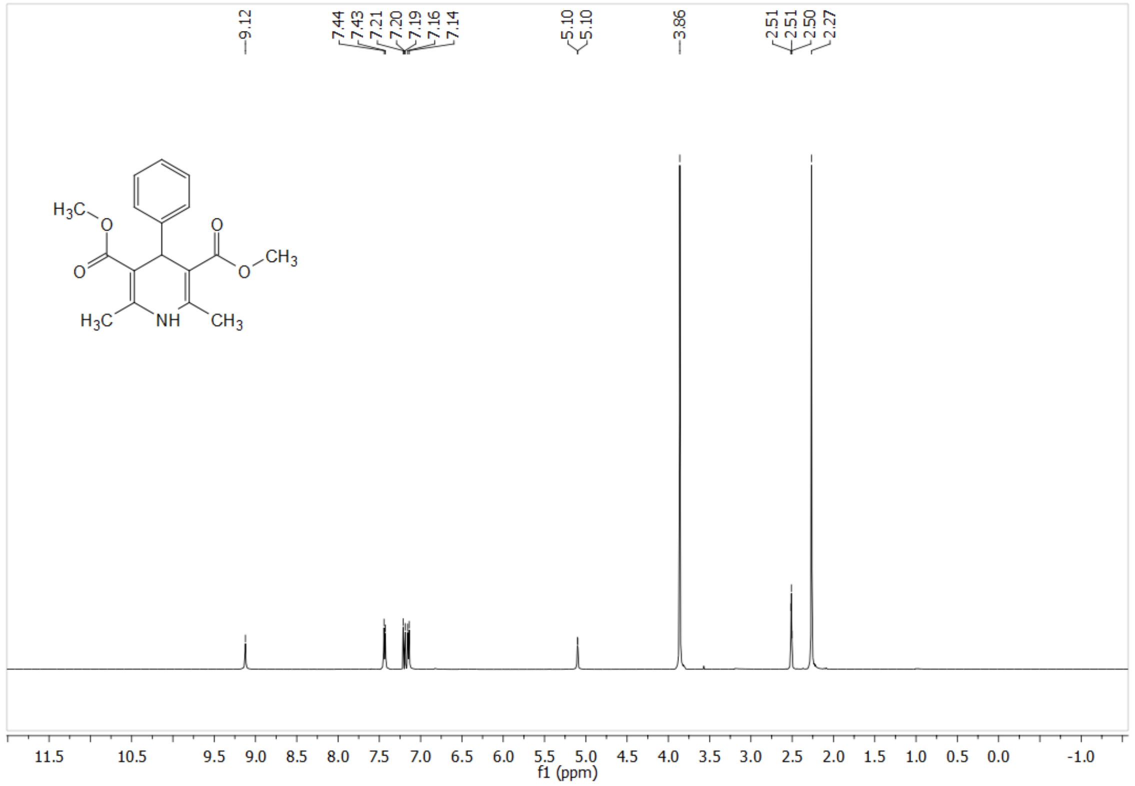

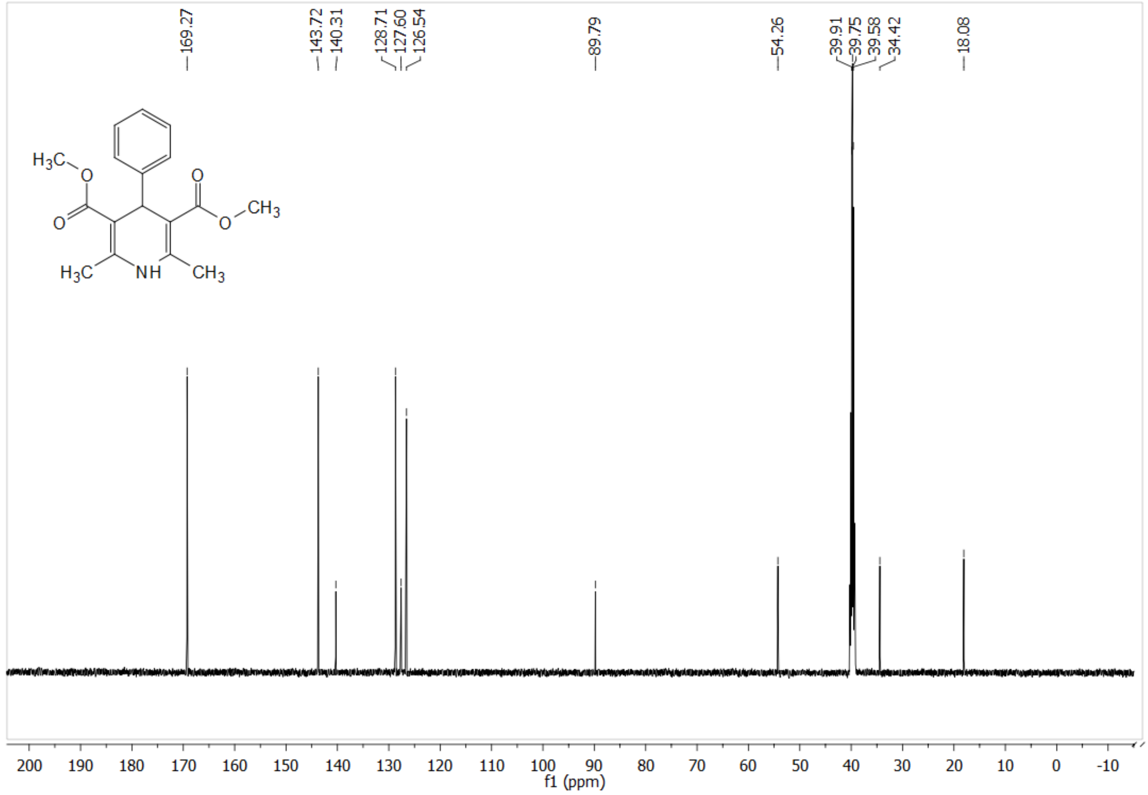

^{1}H and ^{13}C NMR Spectra of Dimethyl 4-(2-hydroxyphenyl)-2,6-dimethyl-1,4-dihydropyridine-3,5 dicarboxylate (3m)

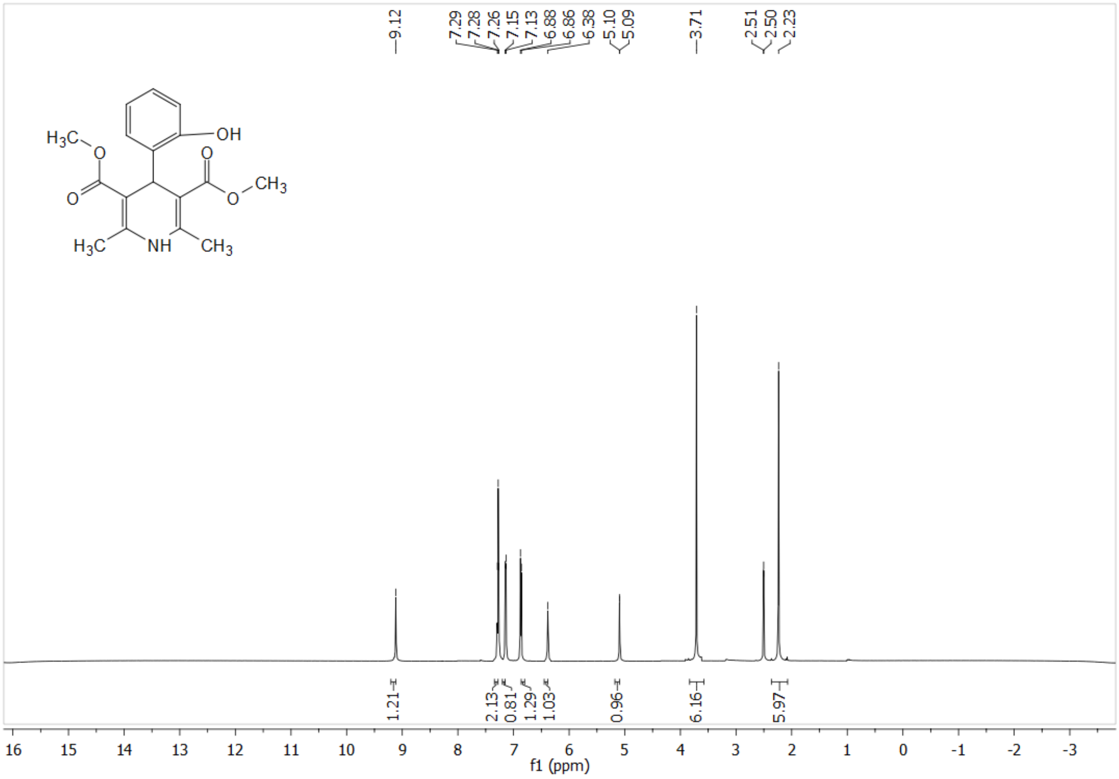

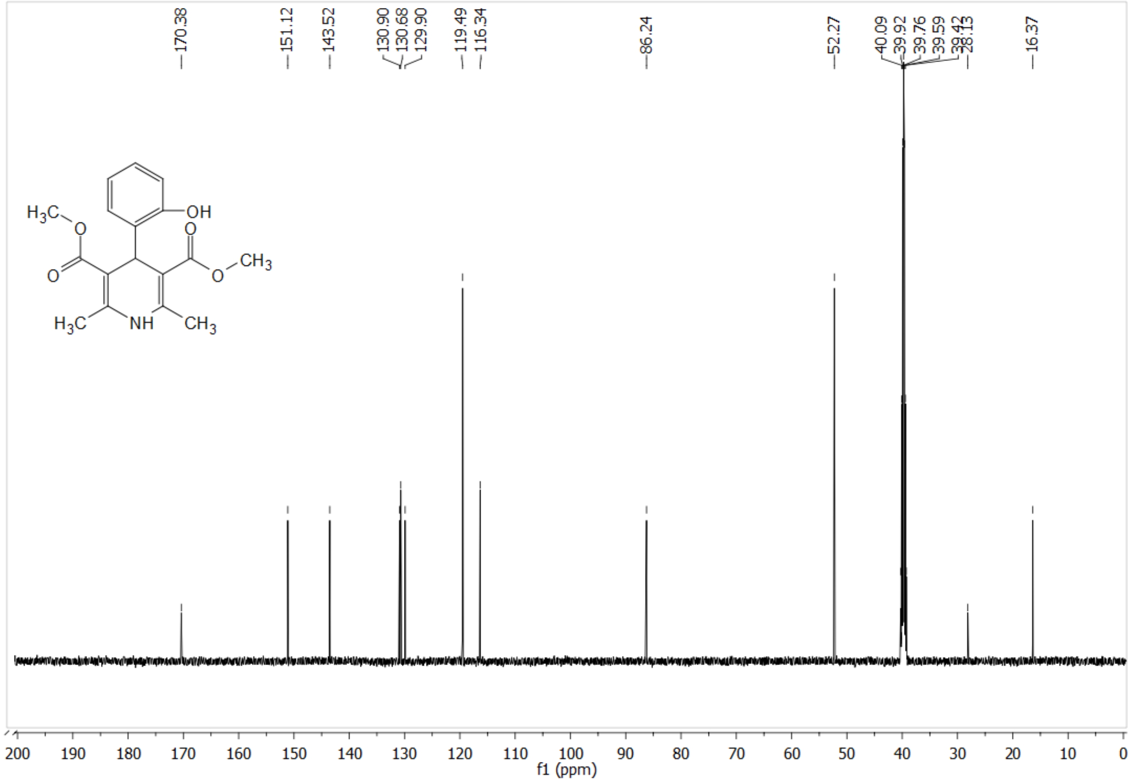

^{1}H and ^{13}C NMR Spectra of Dimethyl 4-(4-methoxyphenyl)-2,6-dimethyl-1,4-dihydropyridine-3,5-dicarboxylate (3n)

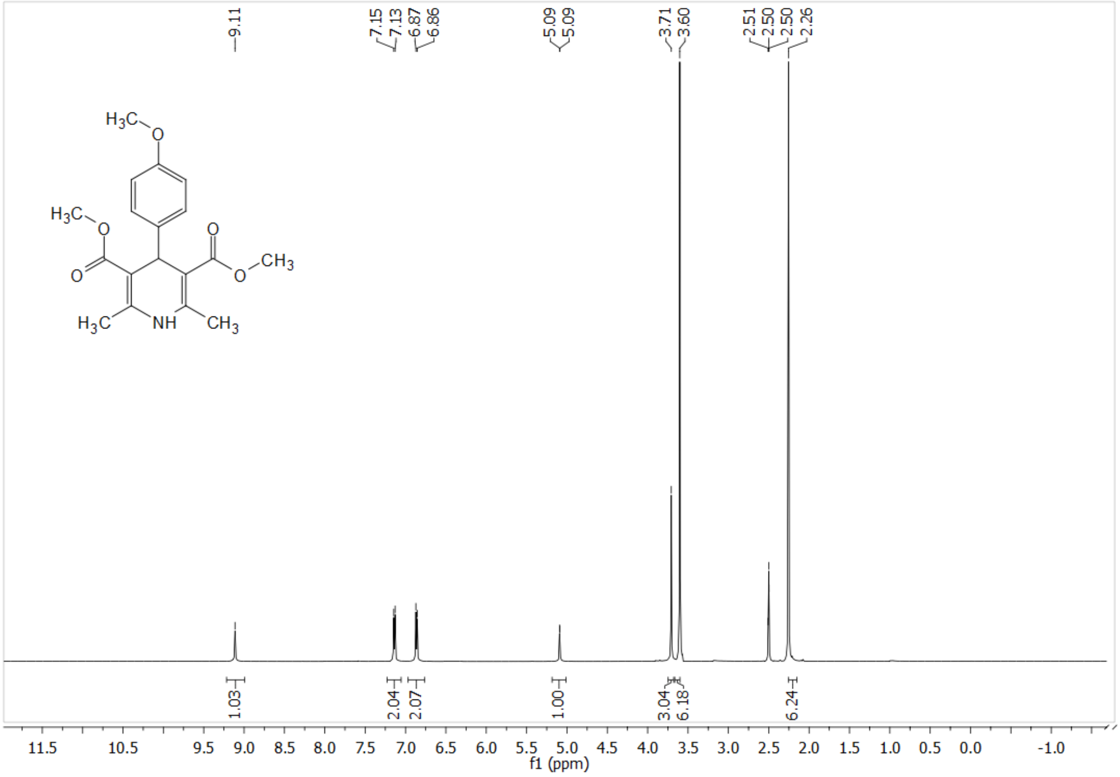

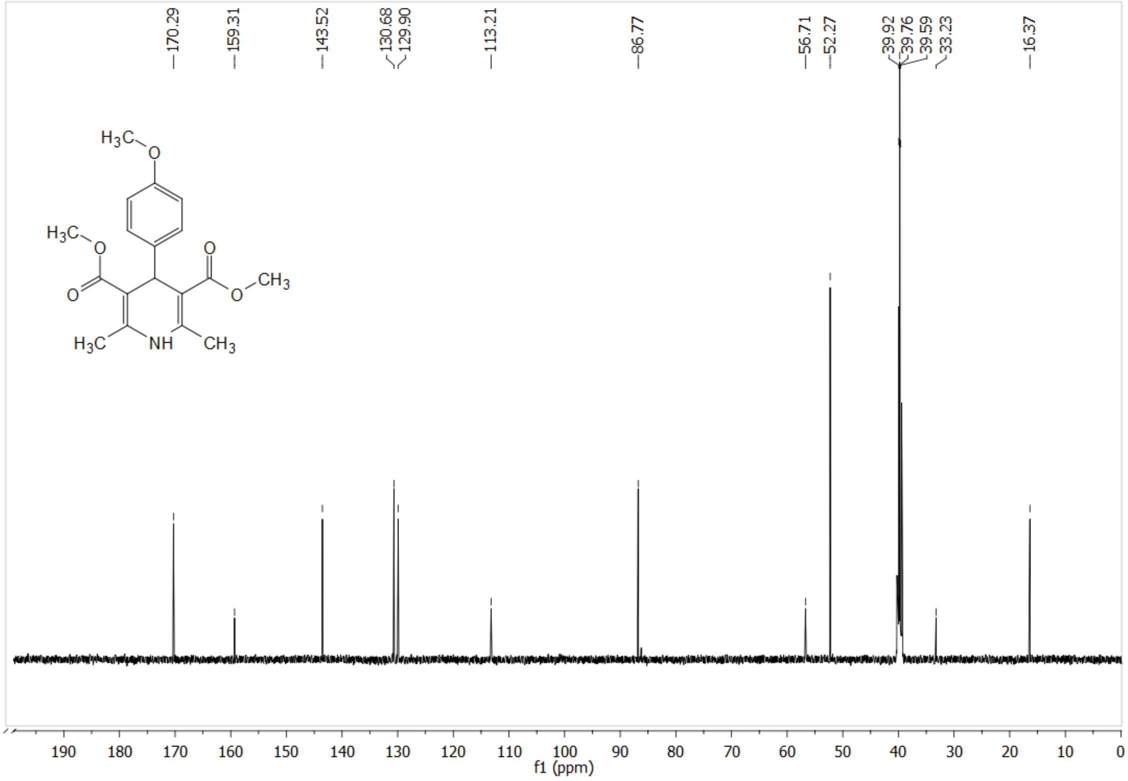

^{1}H and ^{13}C NMR Spectra of Dimethyl 4-(2-bromophenyl)-2,6-dimethyl-1,4-dihydropyridine-3,5-dicarboxylate (3o)

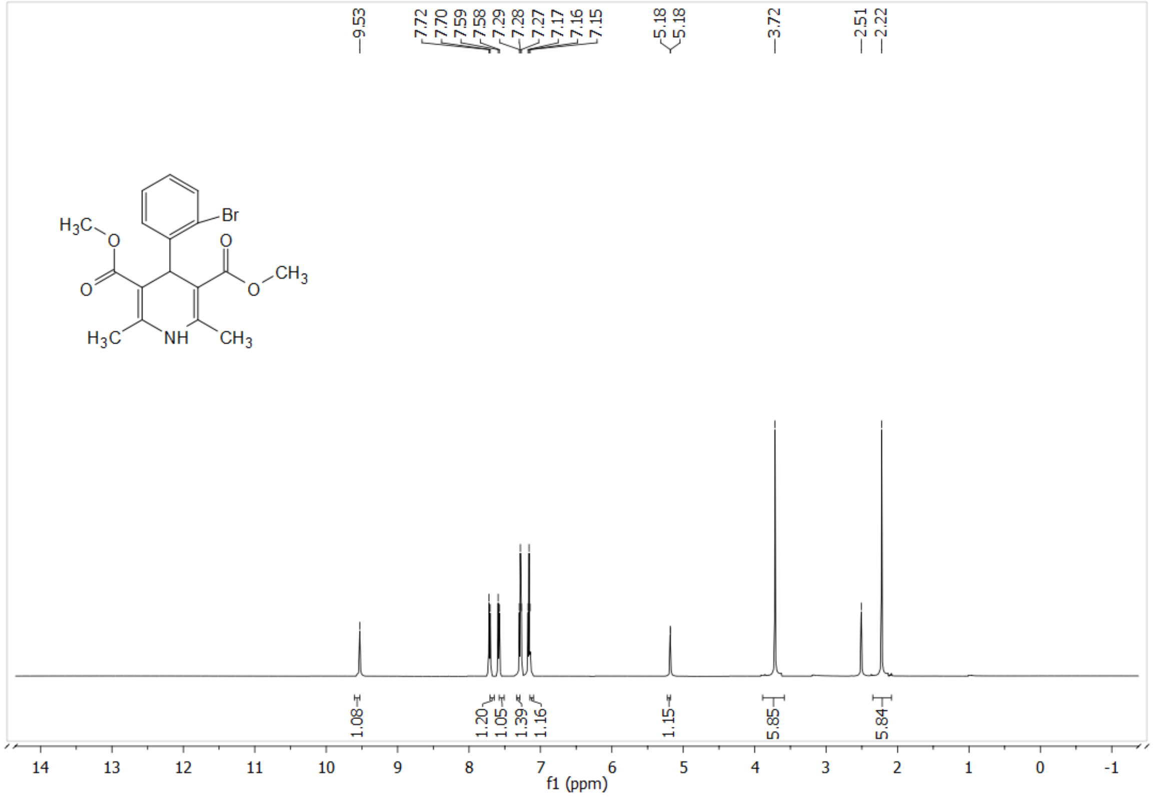

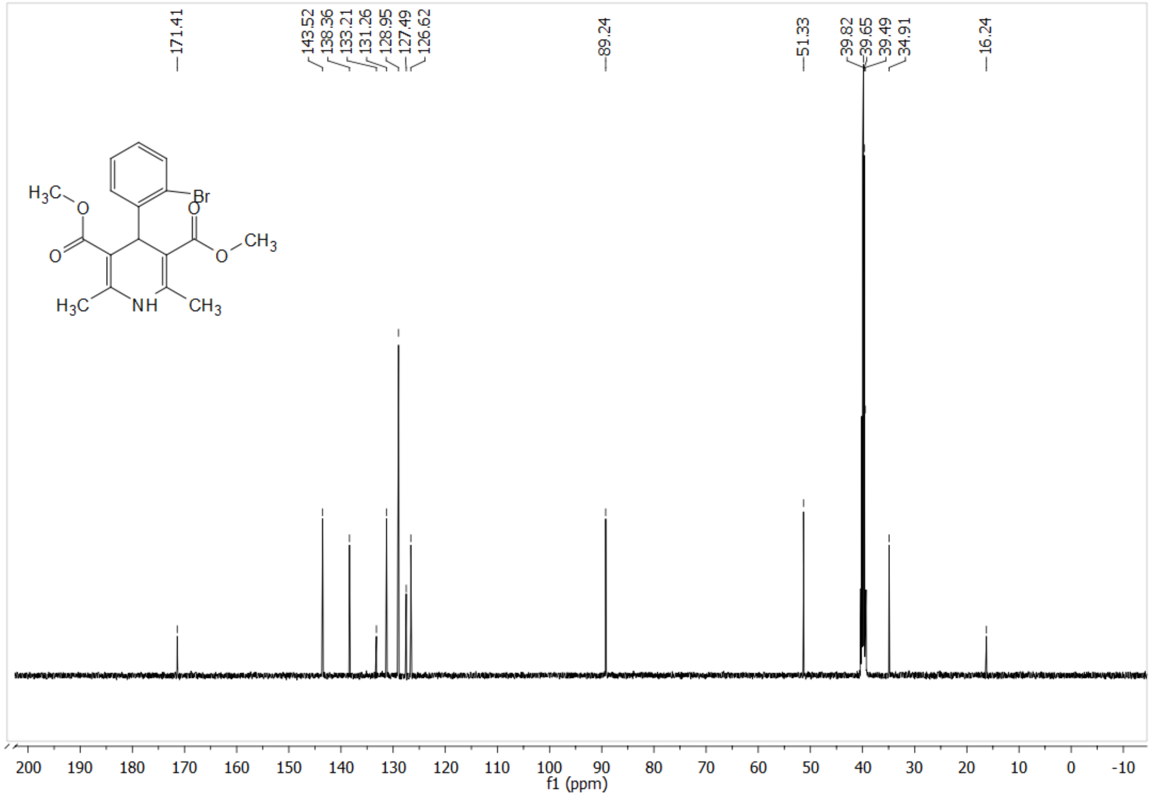

^{1}H and ^{13}C NMR Spectra of Dimethyl 4-(4-chlorophenyl)-2,6-dimethyl-1,4-dihydropyridine-3,5-dicarboxylate (3p)

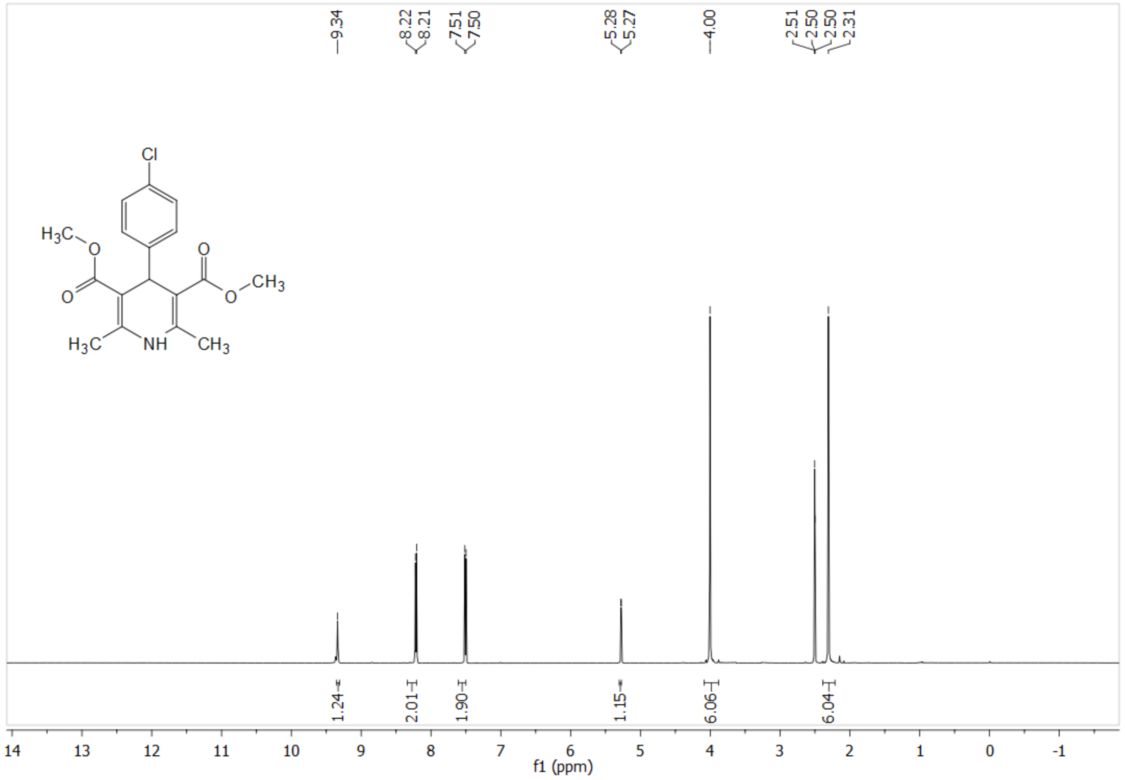

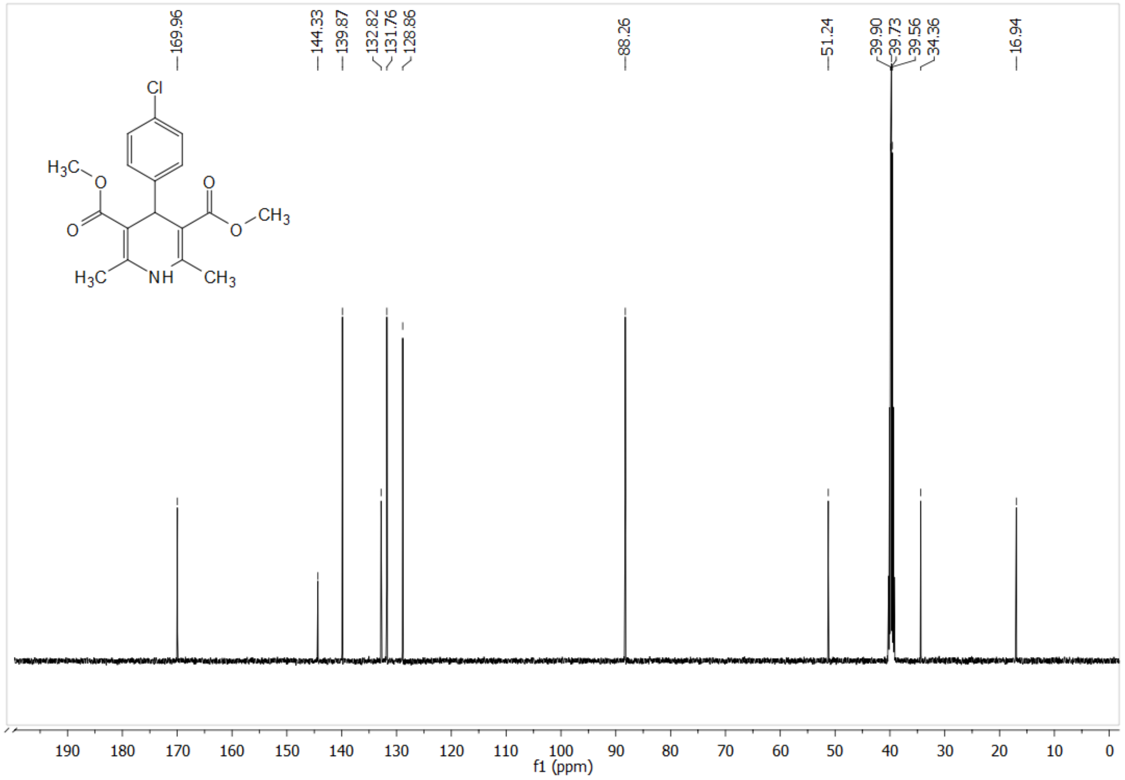

^1H, ^{13}C and ^{19}F NMR Spectra of Dimethyl 4-(4-fluorophenyl)-2,6-dimethyl-1,4-dihydropyridine-3,5-dicarboxylate (3q)

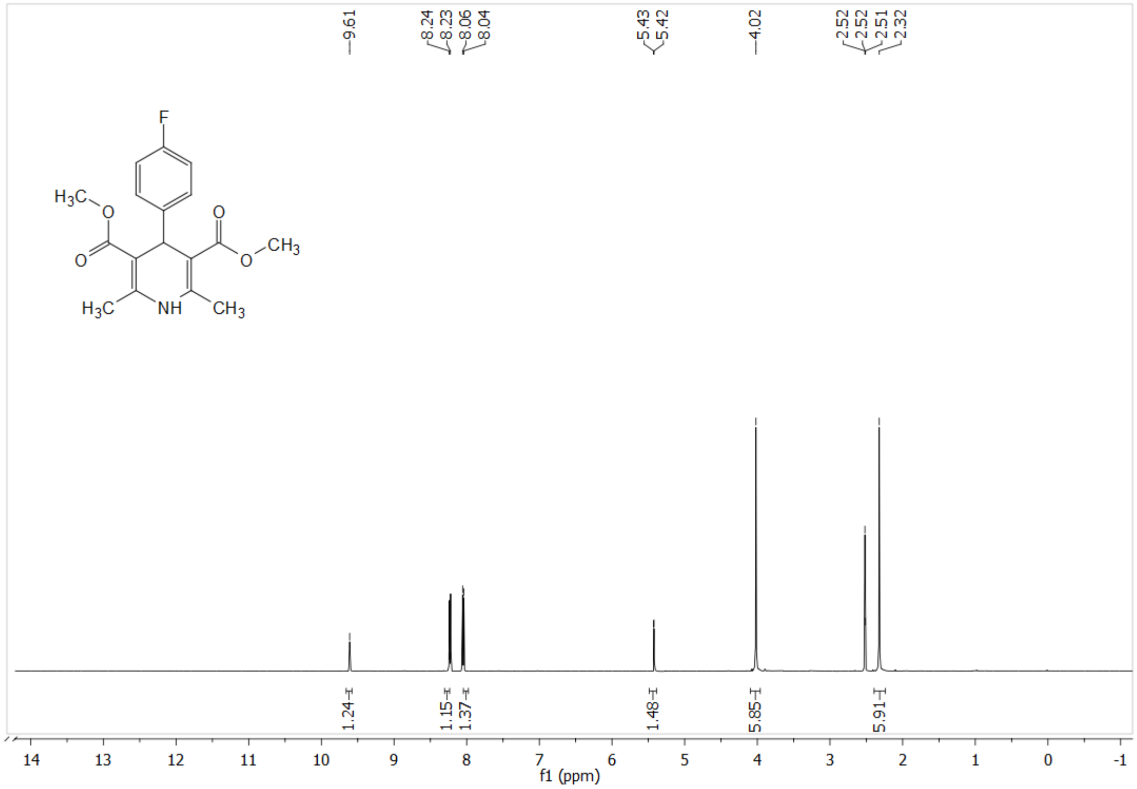

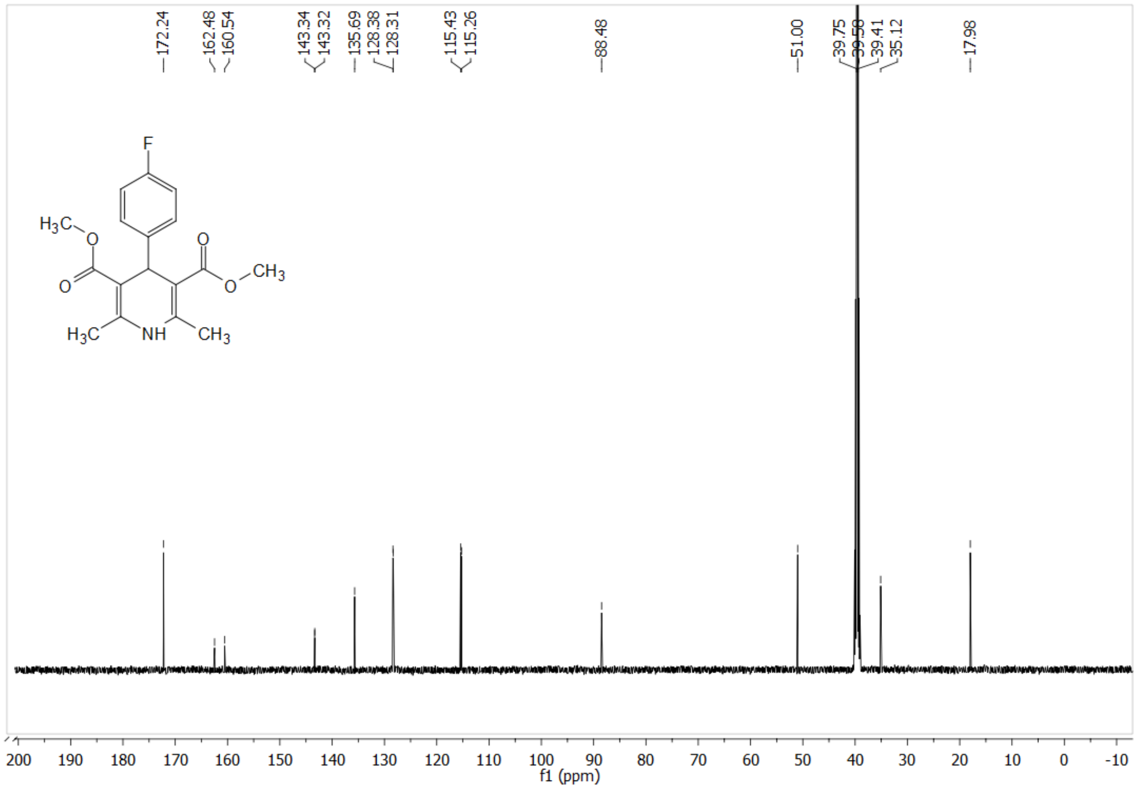

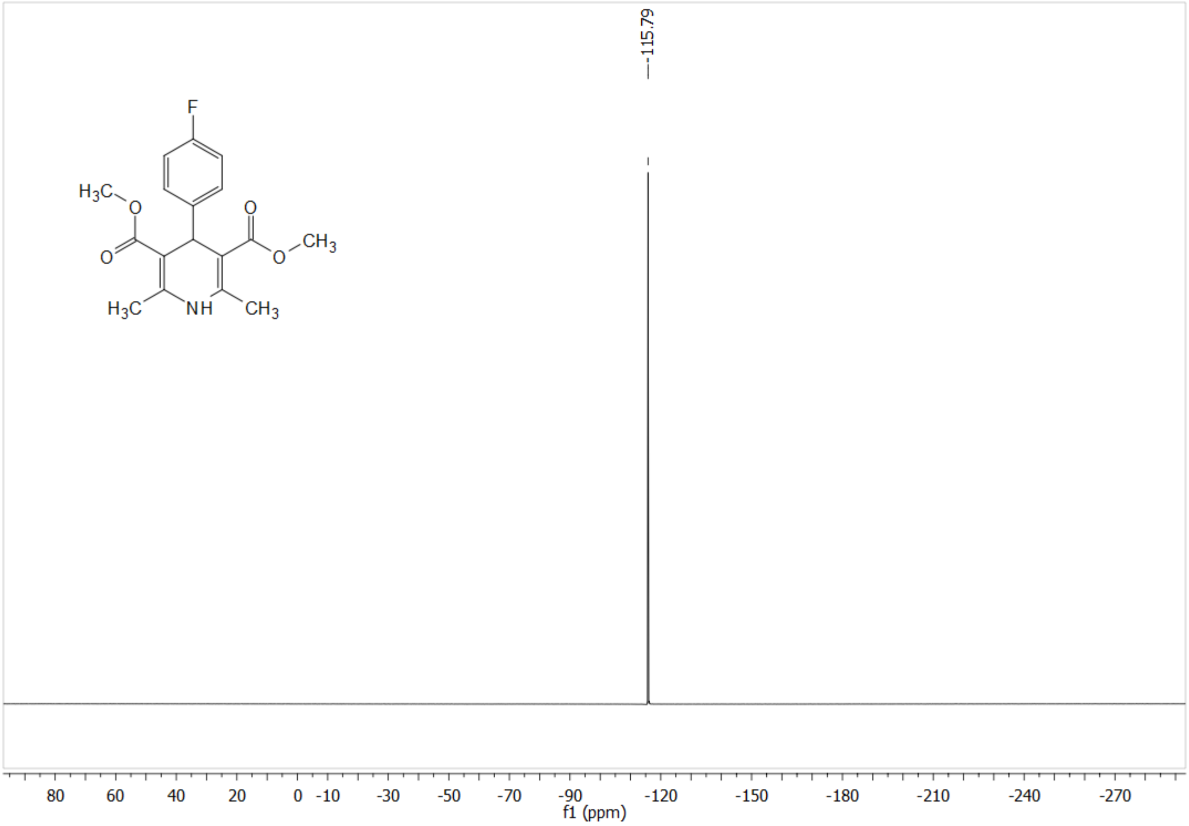

--115.79
F
H3C
O
O
O
O
CH3
H3C
NH
CH3
80
60
40
20
0
-10
-30
-50
-70
-90
-120
-150
-180
-210
-240
-270
f1 (ppm)

^{1}H and ^{13}C NMR Spectra of Dimethyl 4-(2-hydroxy-3-methoxyphenyl)-2,6-dimethyl-1,4-dihydropyridine-3,5-dicarboxylate (3r)

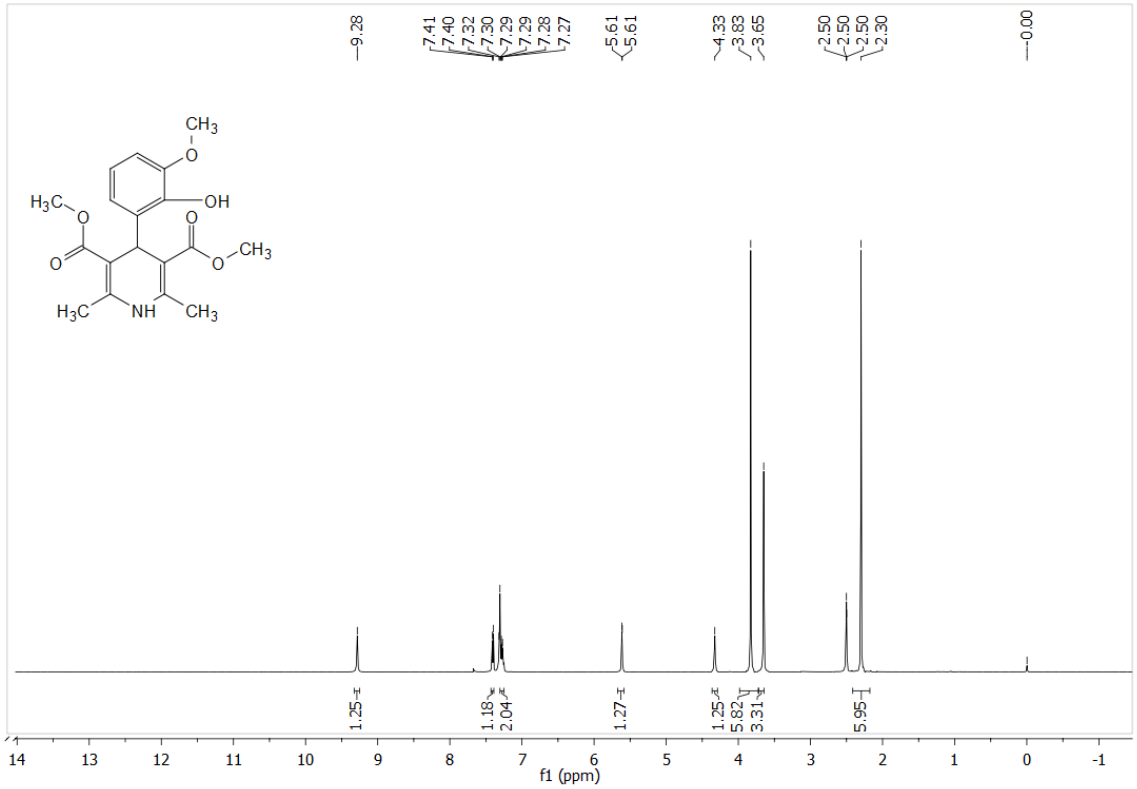

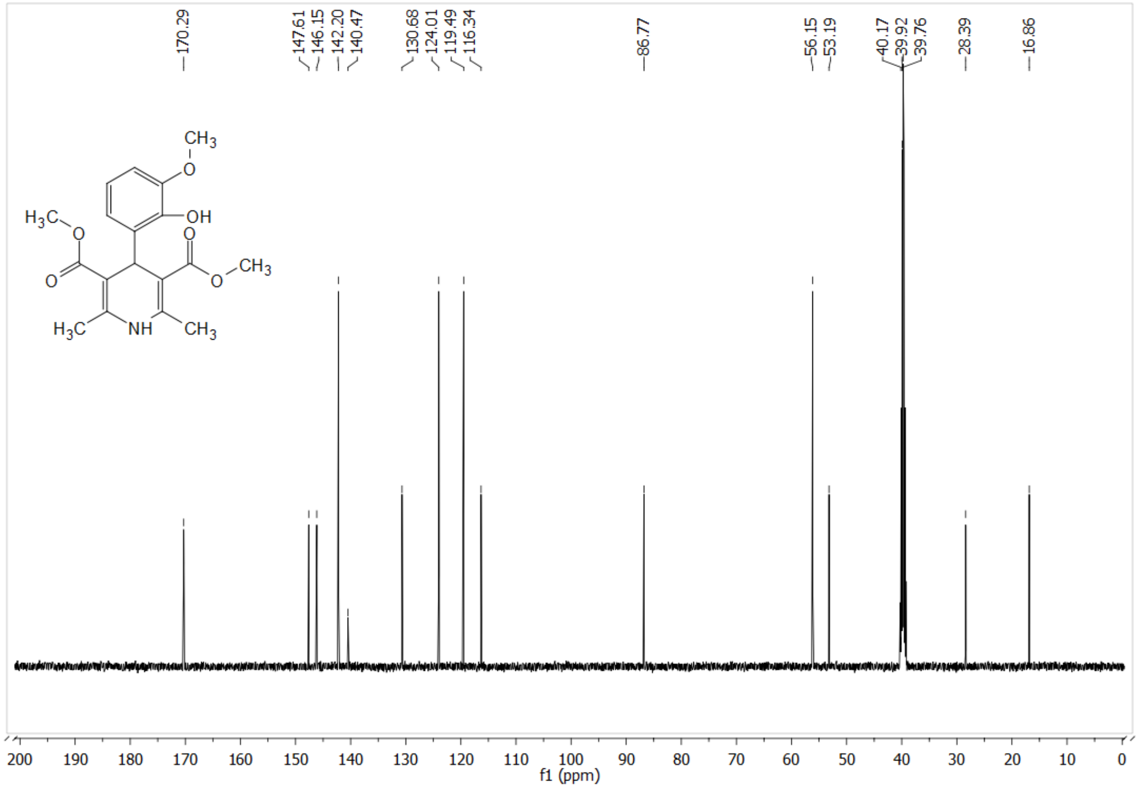

^{1}H and ^{13}C NMR Spectra of Dimethyl 2,6-dimethyl-4-(naphthalen-1-yl)-1,4-dihydropyridine-3,5-dicarboxylate (3s)

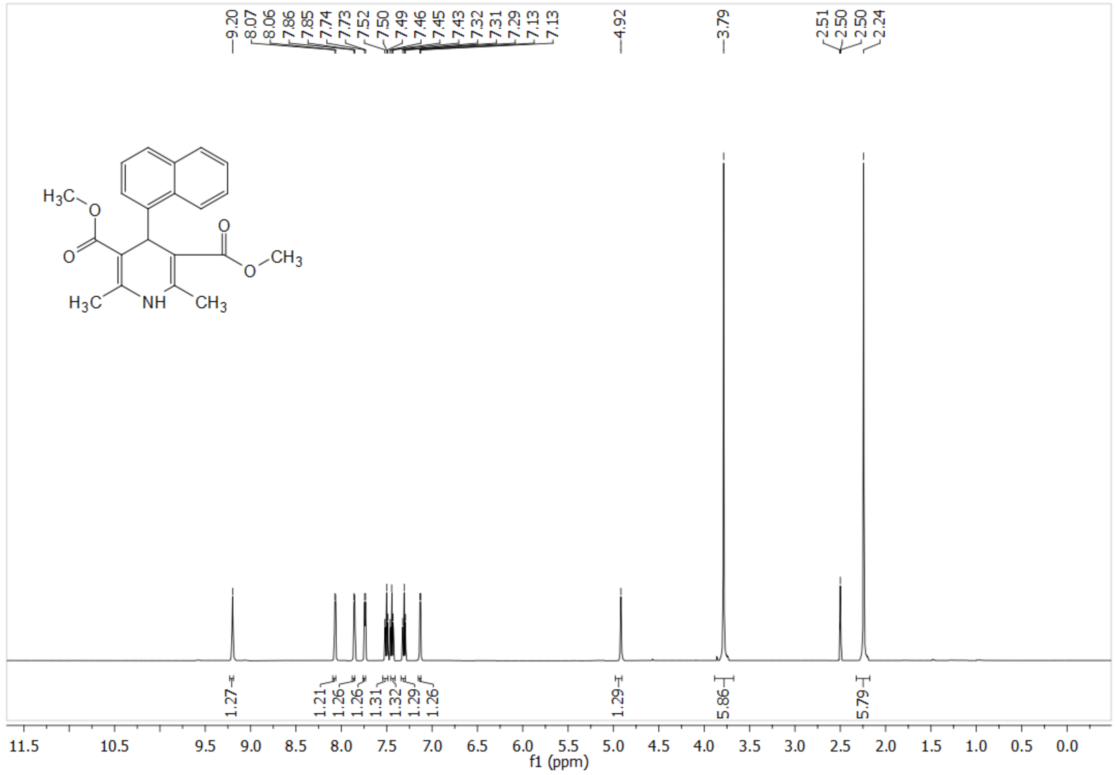

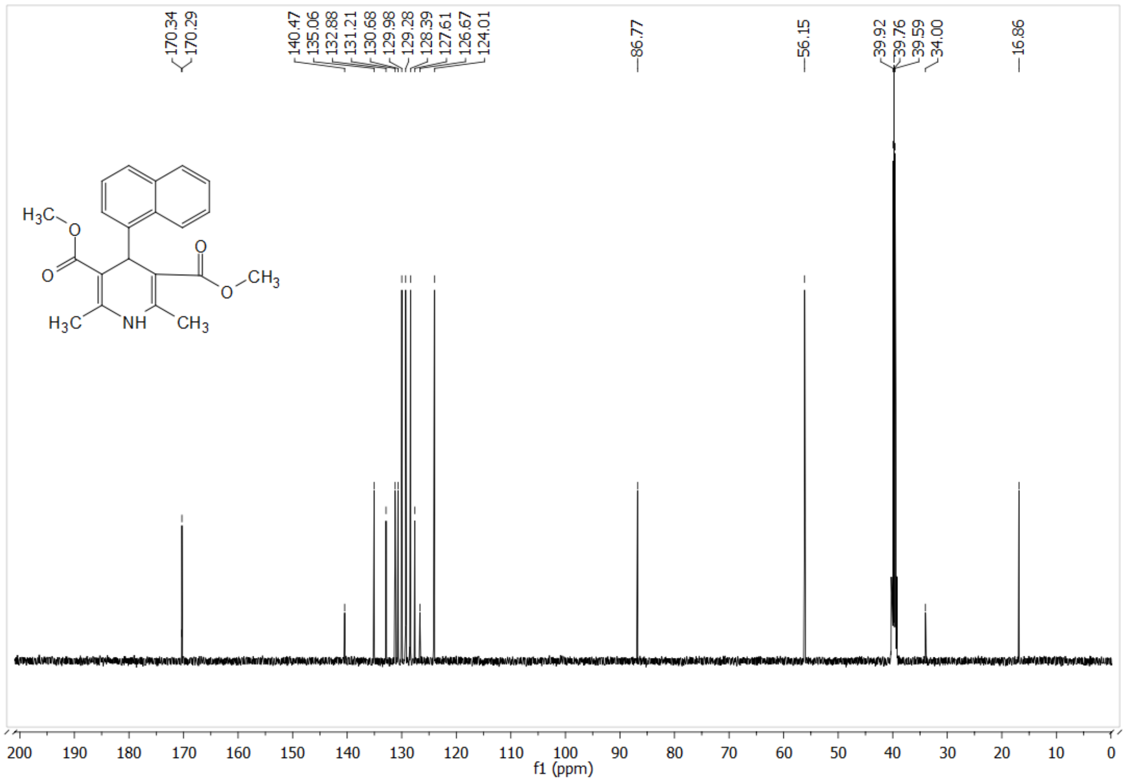

^{1}H and ^{13}C NMR Spectra of Dimethyl 2,6-dimethyl-4-(thiophen-2-yl)-1,4-dihydropyridine-3,5-dicarboxylate (3t)

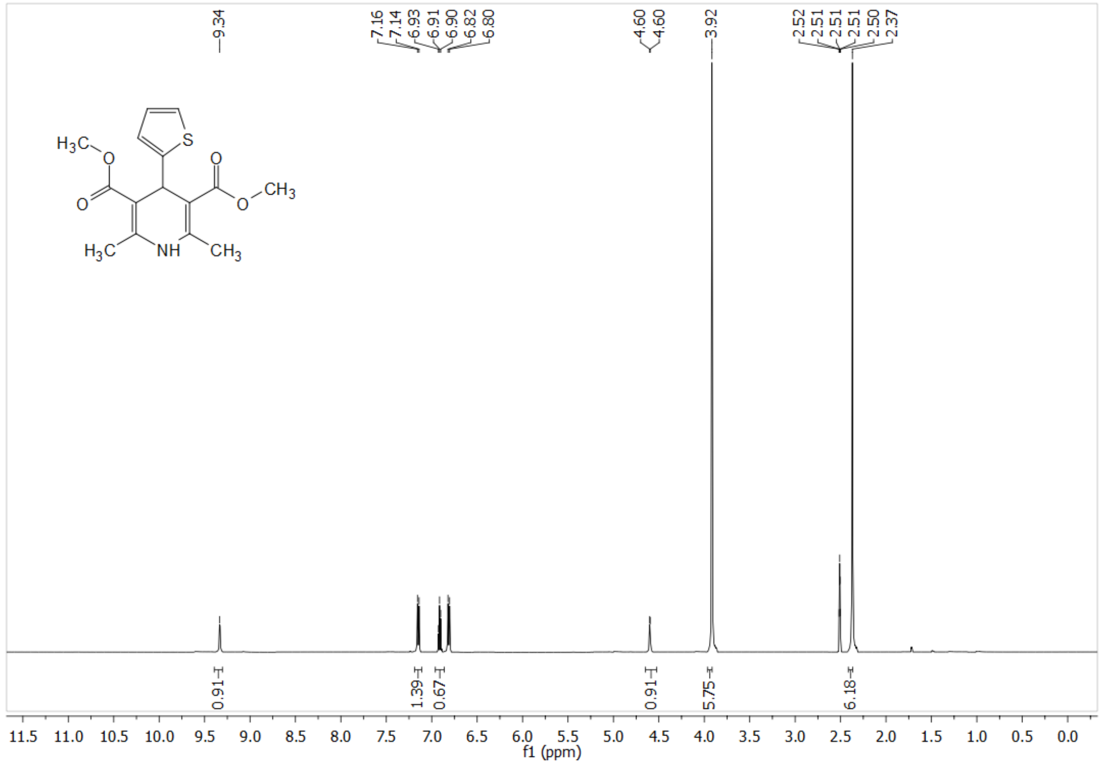

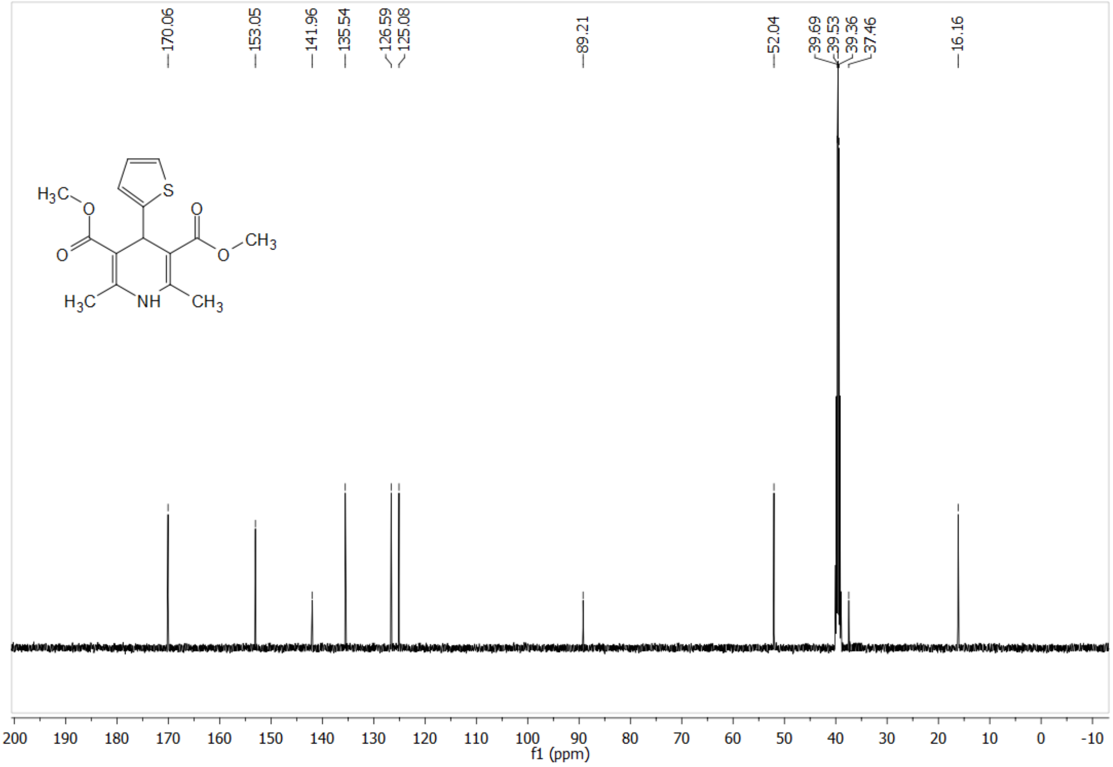

^{1}H and ^{13}C NMR Spectra of Dimethyl 2',6'-dimethyl-1',4'-dihydro-[2,4'-bipyridine]-3',5'-dicarboxylate (3u)

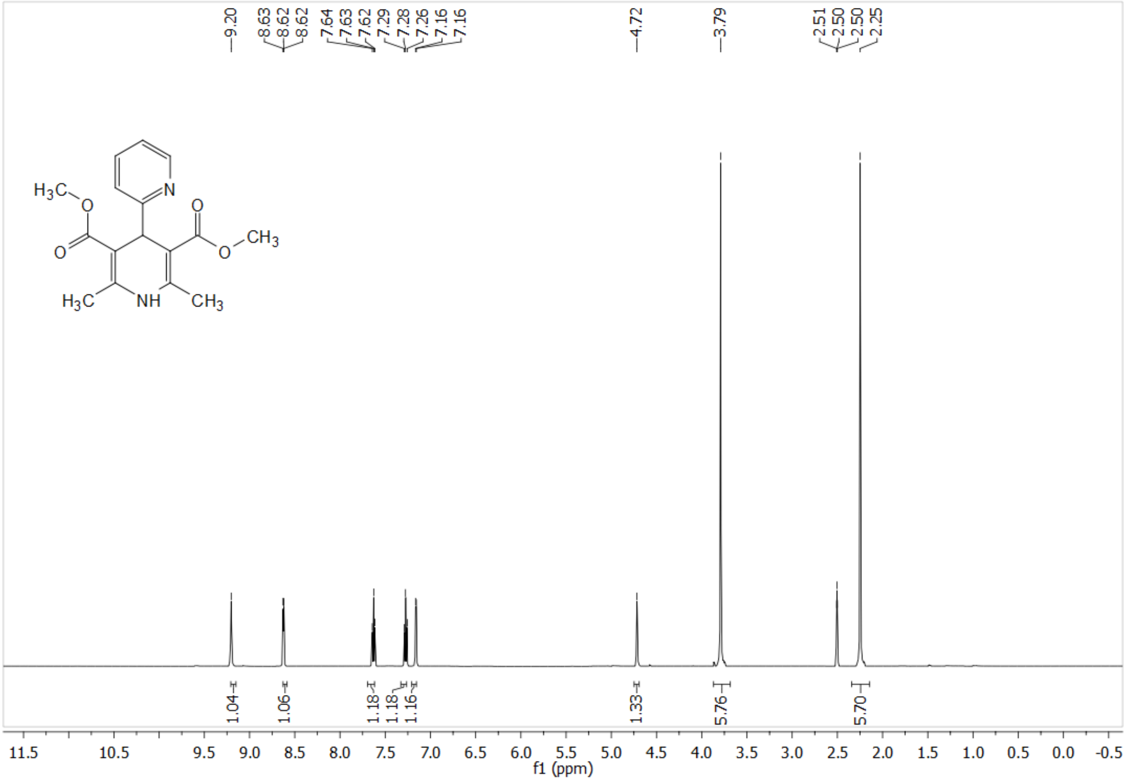

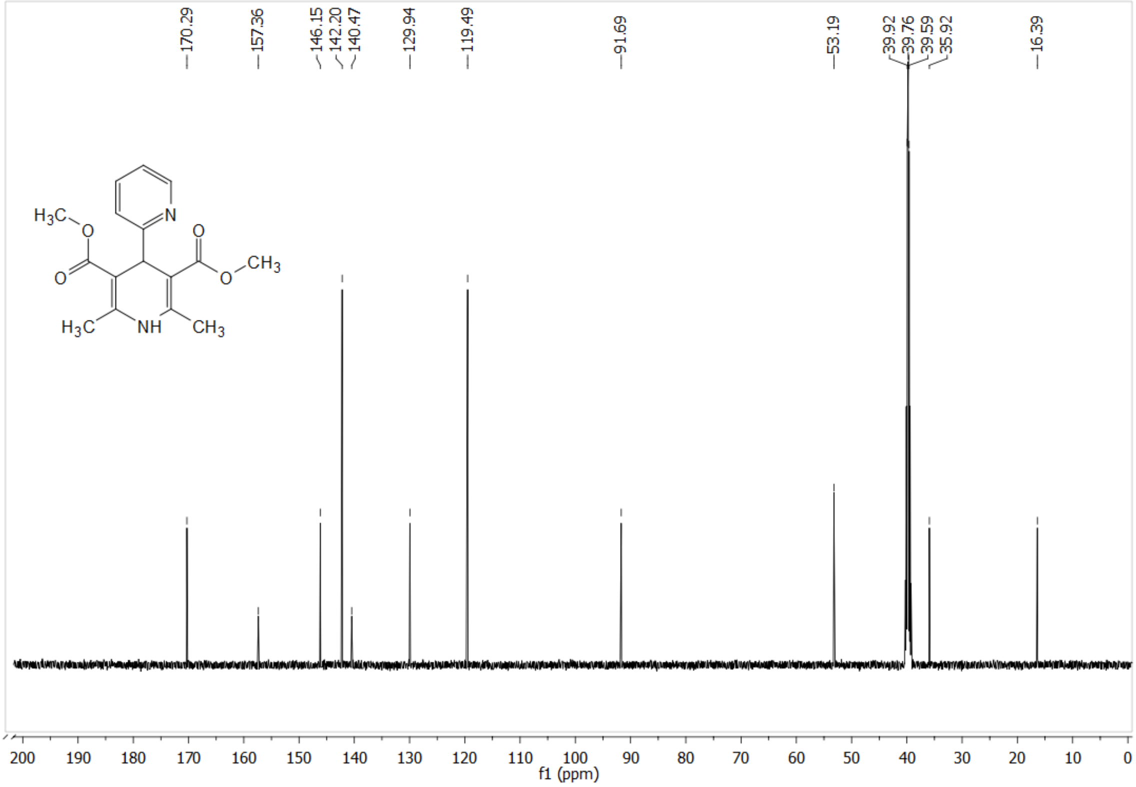

^{1}H and ^{13}C NMR Spectra of 9-phenyl-3,4,6,7,9,10-hexahydroacridine-1,8(2H,5H)-dione (3v)

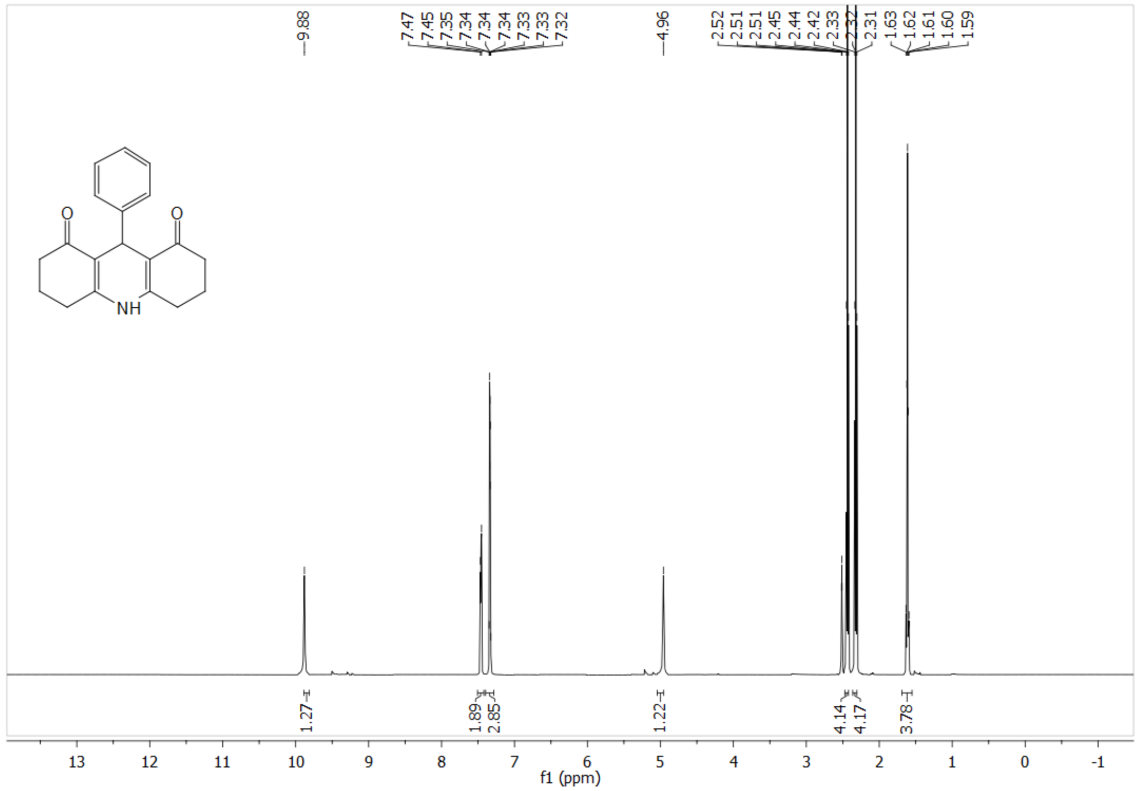

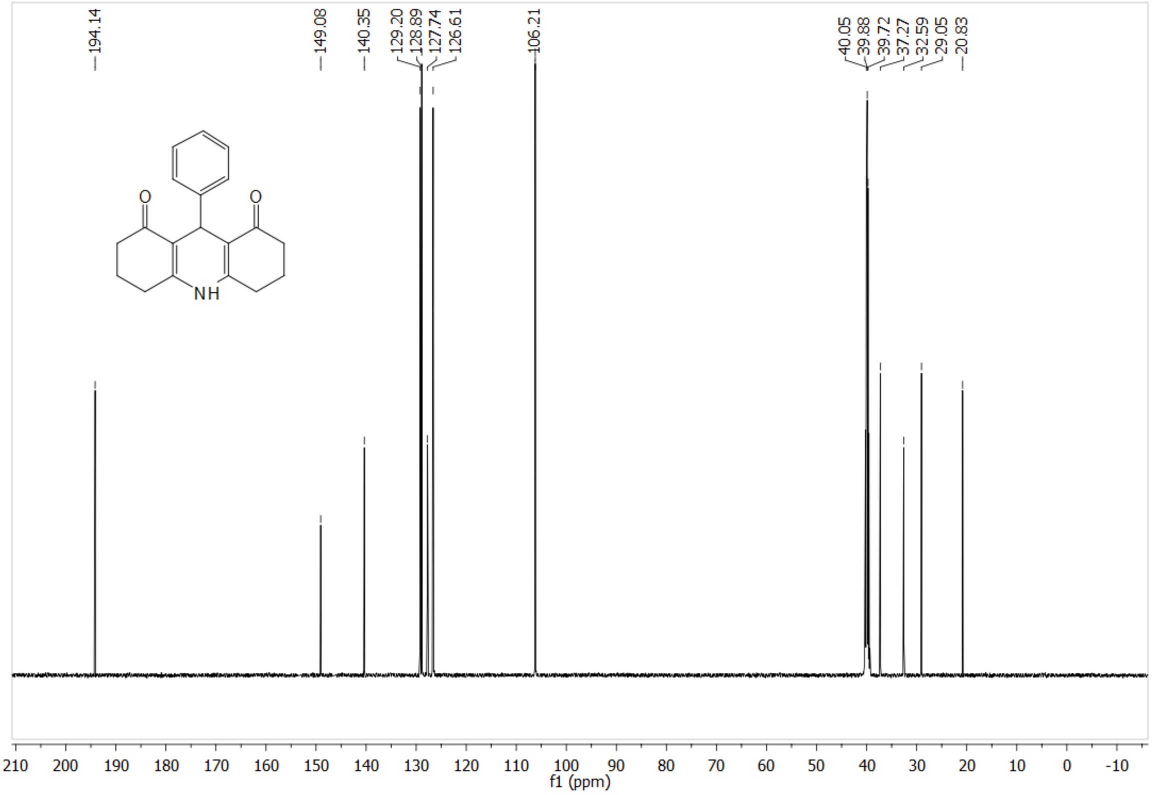

^{1}H and ^{13}C NMR Spectra of 9-(4-methoxyphenyl)-3,4,6,7,9,10-hexahydroacridine-1,8(2H,5H)-dione (3w)

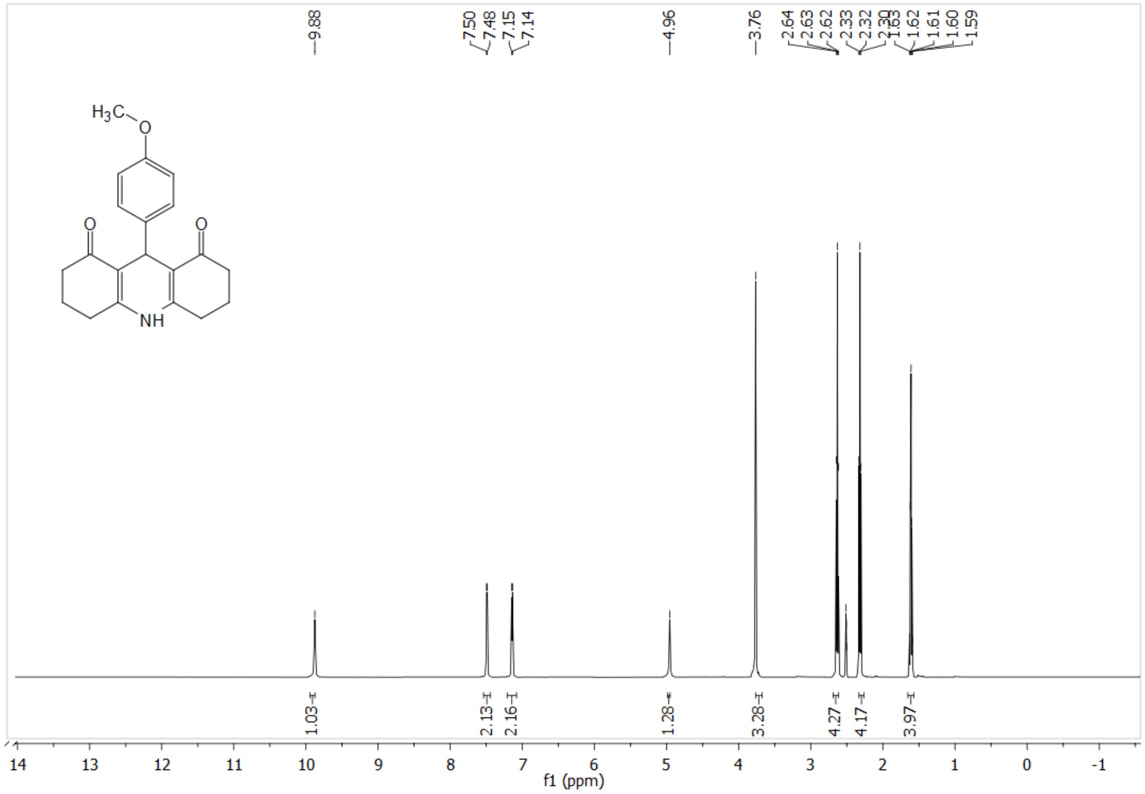

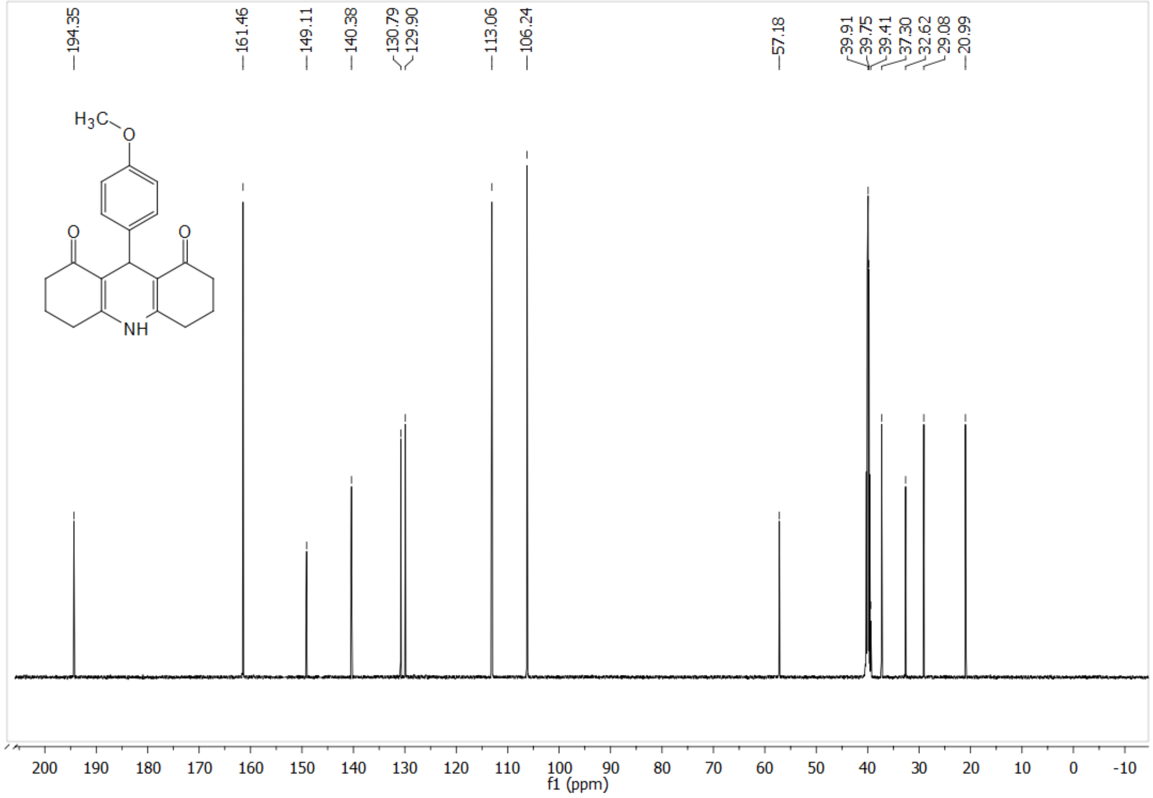

^{1}H and ^{13}C NMR Spectra of 9-(3-chlorophenyl)-3,4,6,7,9,10-hexahydroacridine-1,8(2H,5H)-dione (3x)

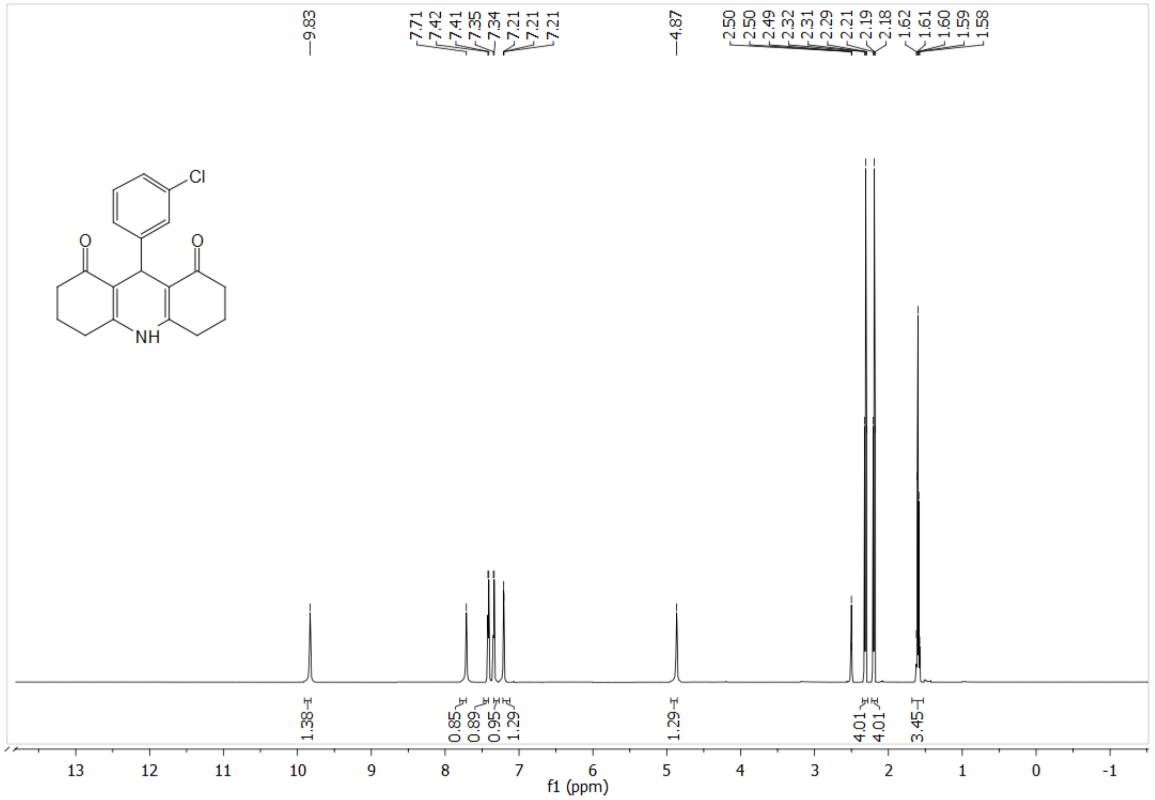

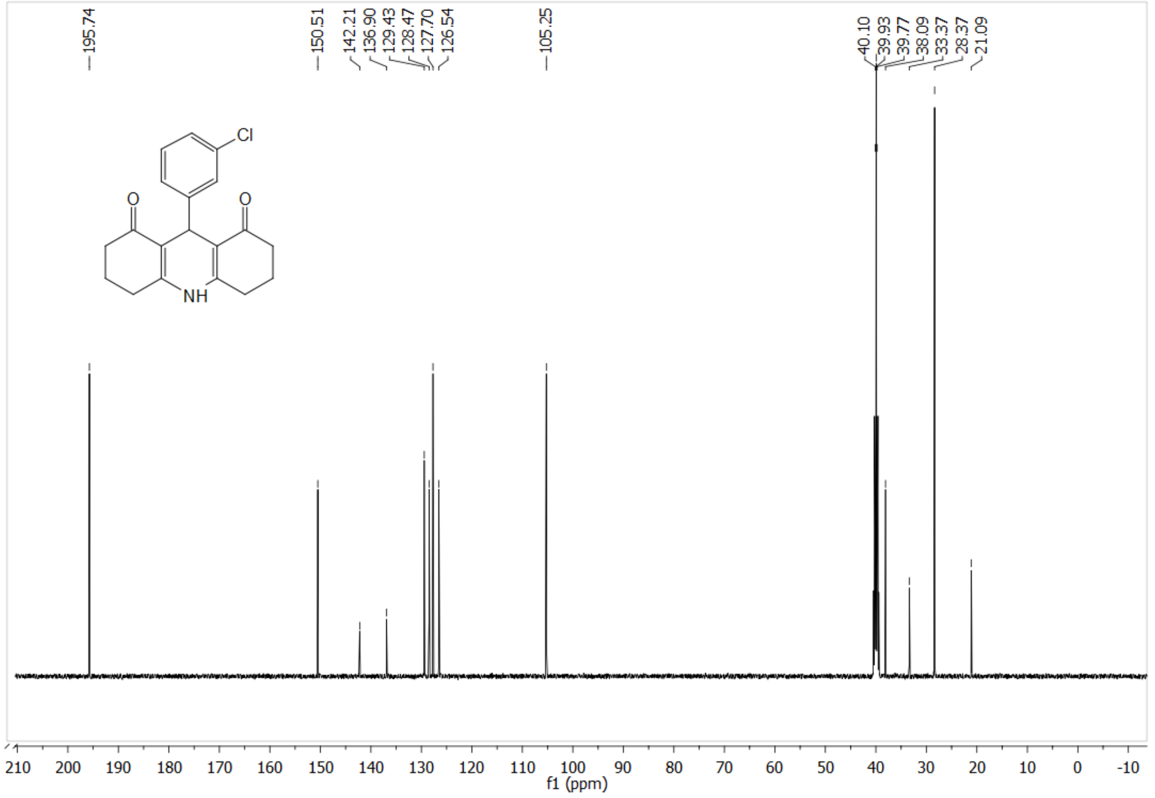

^{1}H and ^{13}C NMR Spectra of 9-(4-bromophenyl)-3,4,6,7,9,10-hexahydroacridine-1,8(2H,5H)-dione (3y)

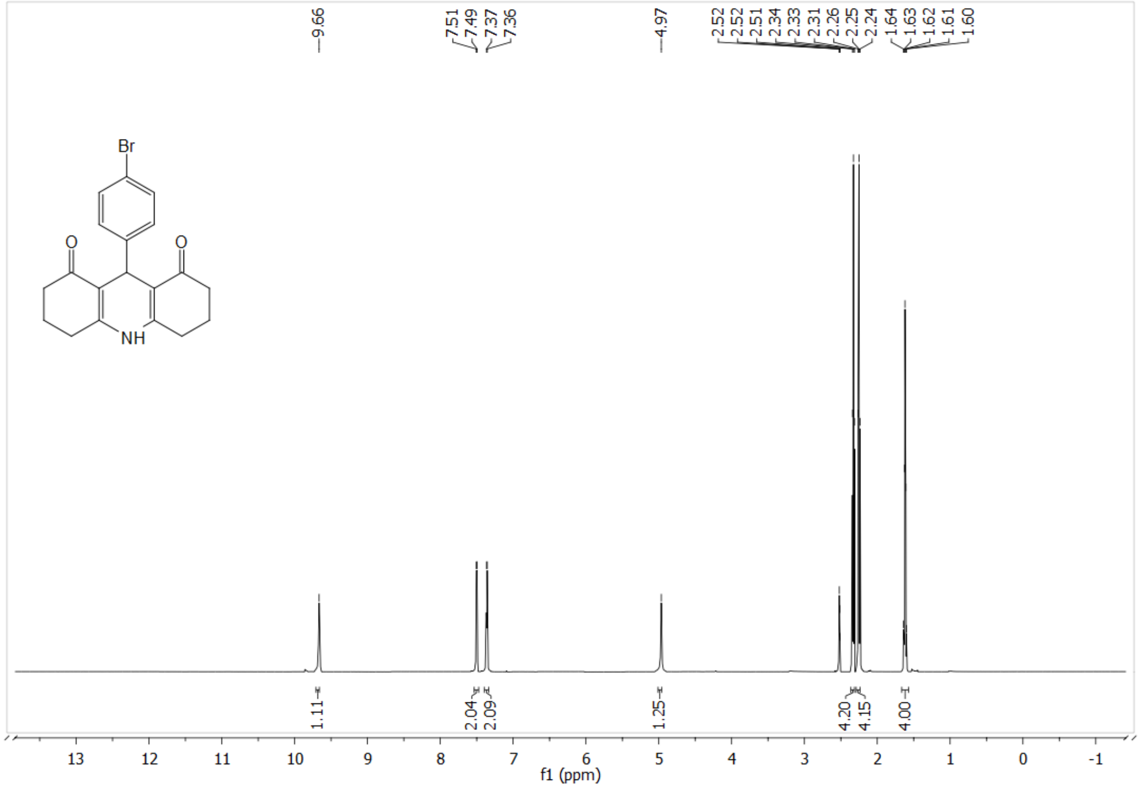

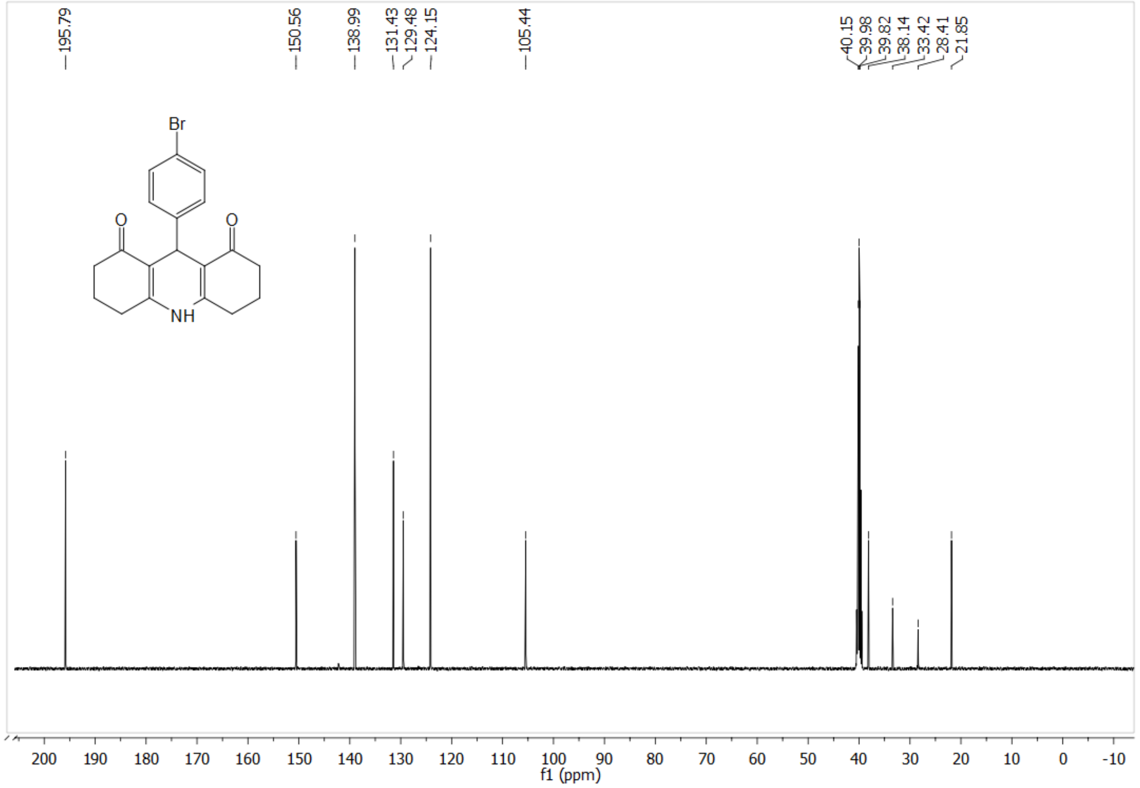

^1H and ^{13}C NMR Spectra of 3,3,6,6-tetramethyl-9-phenyl-3,4,6,7,9,10-hexahydroacridine-1,8(2H,5H)-dione (3z)

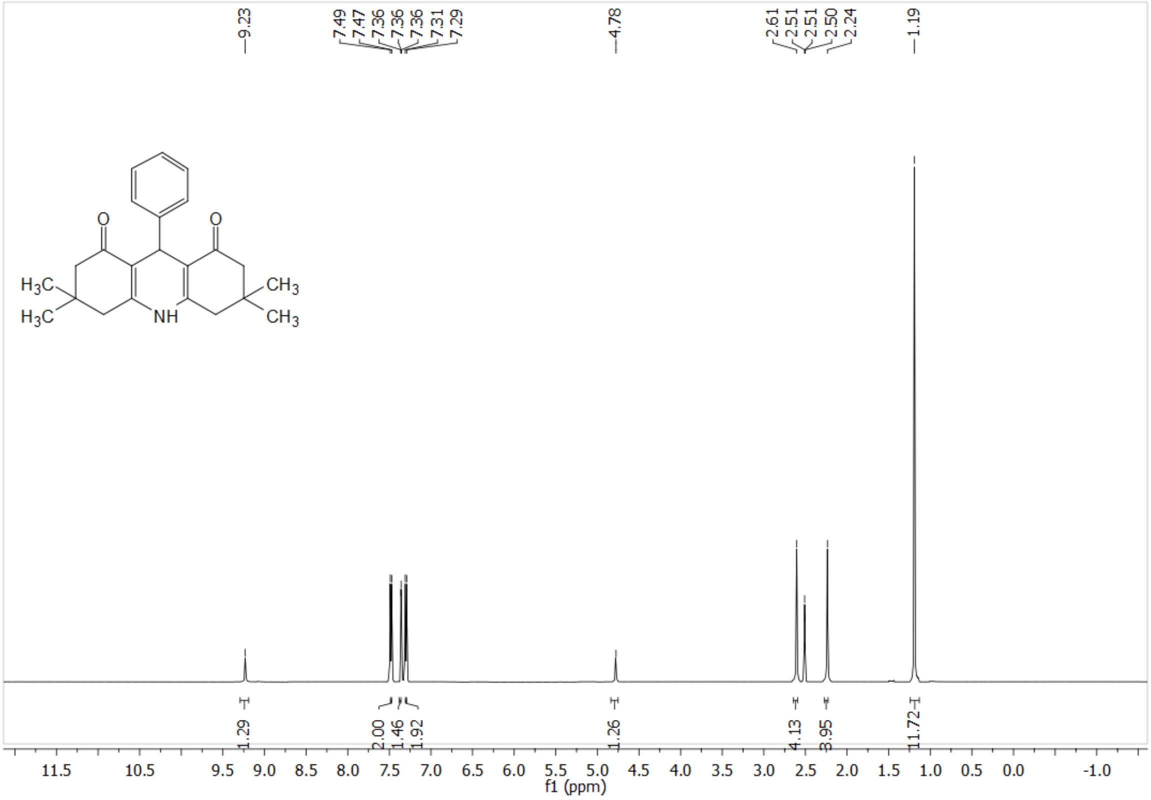

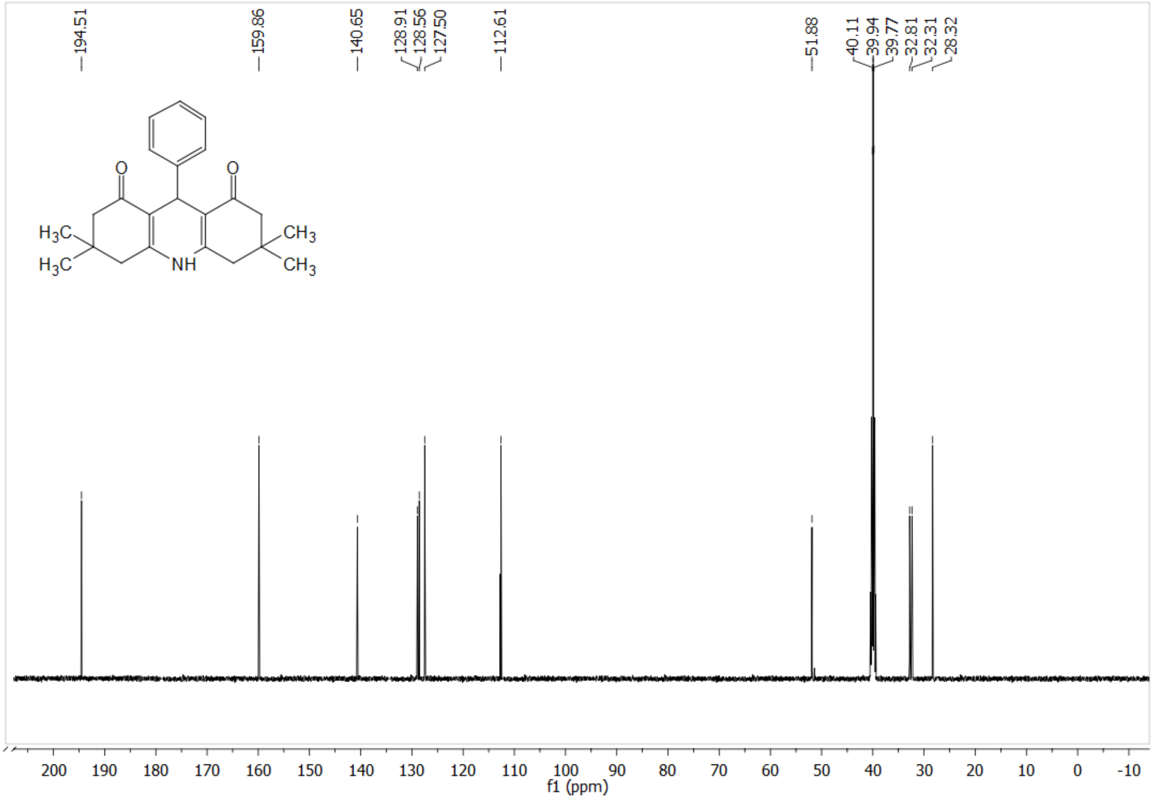

^{1}H and ^{13}C NMR Spectra of 9-(4-methoxyphenyl)-3,3,6,6-tetramethyl-3,4,6,7,9,10-hexahydroacridine-1,8(2H,5H)-dione (3aa)

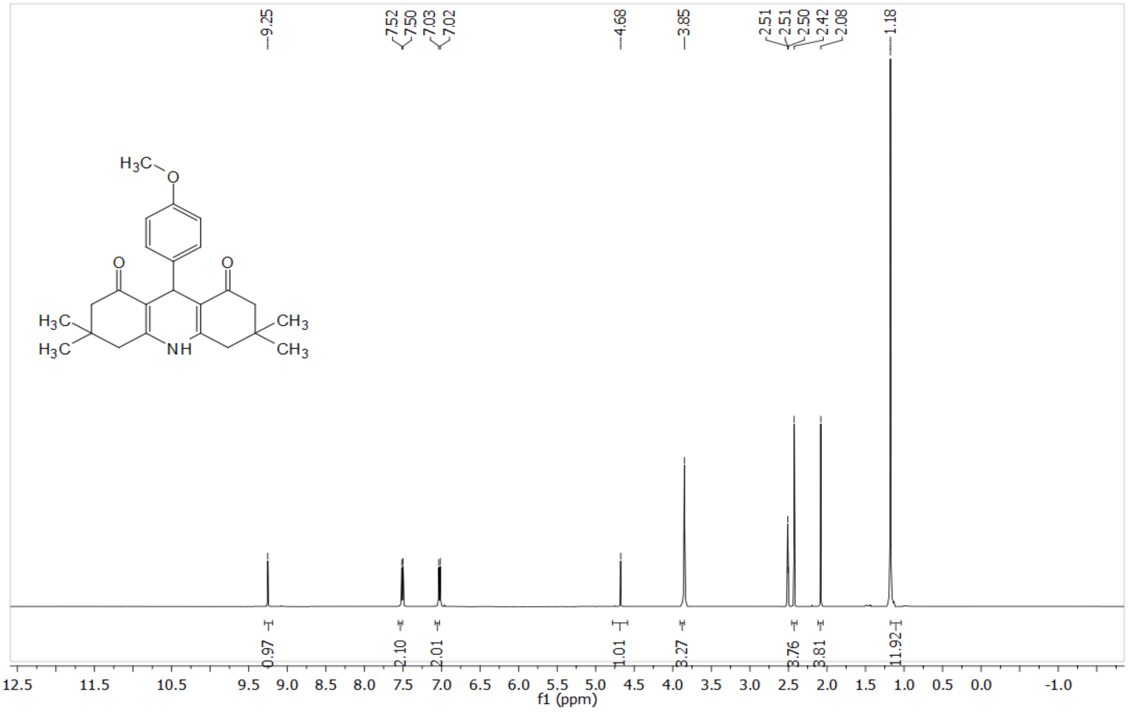

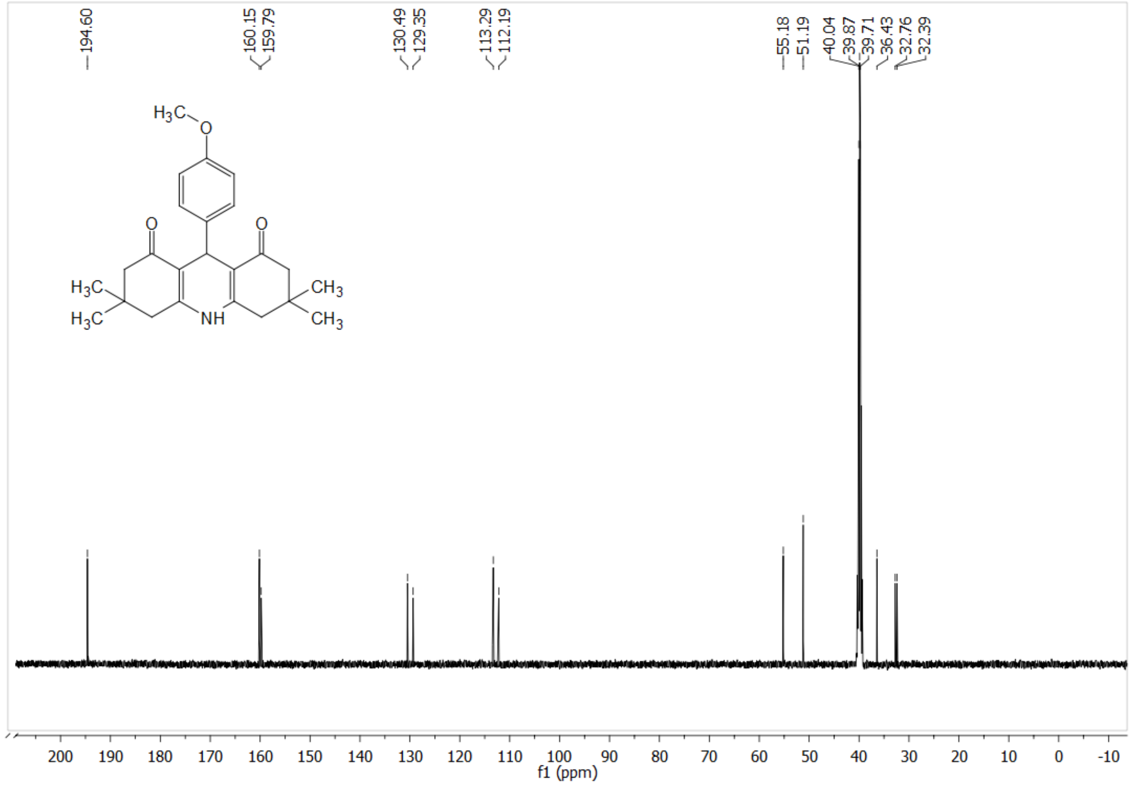

^{1}H and ^{13}C NMR Spectra of 9-(3-chlorophenyl)-3,3,6,6-tetramethyl-3,4,6,7,9,10-hexahydroacridine-1,8(2H,5H)-dione (3ab)

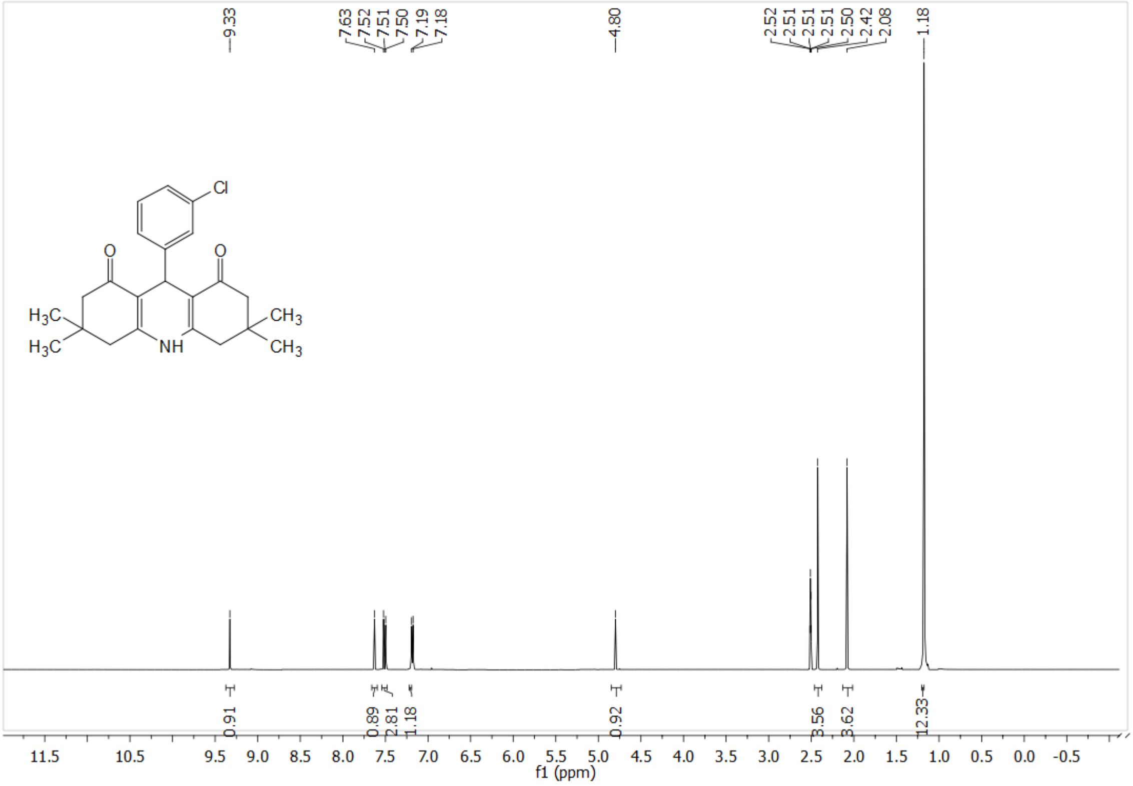

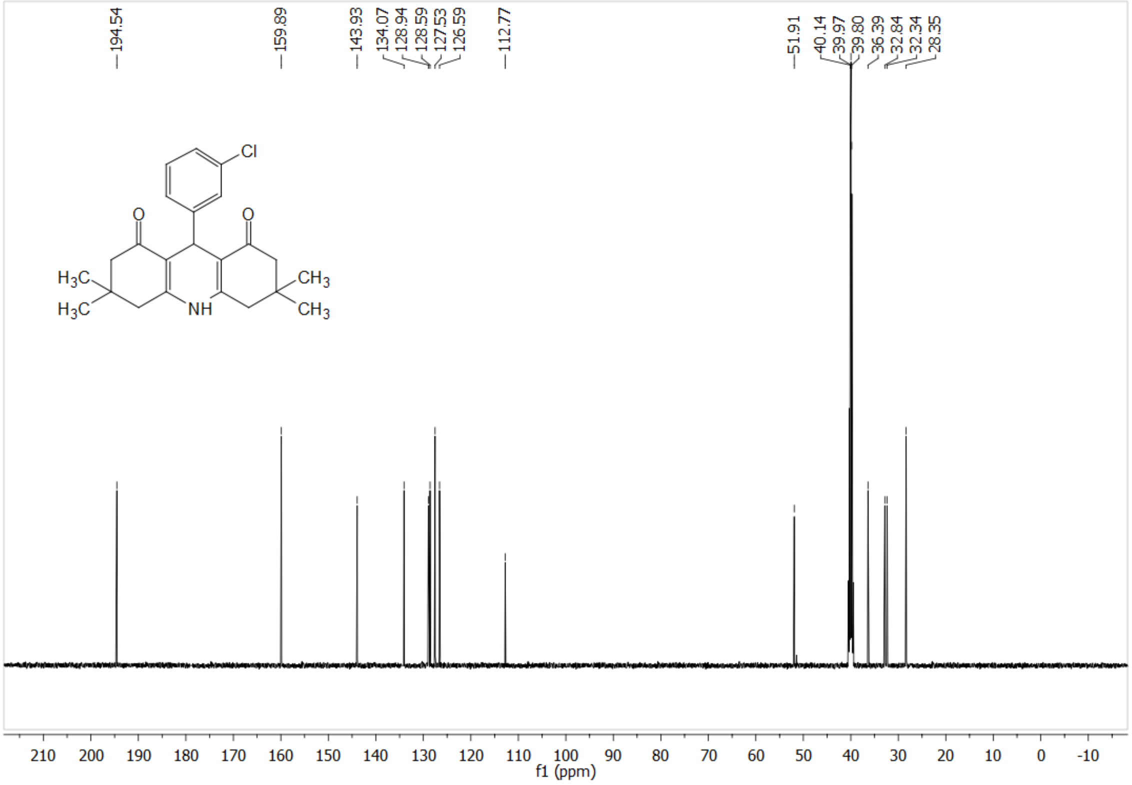

^{1}H and ^{13}C NMR Spectra of 3,3,6,6-tetramethyl-9-(pyridin-2-yl)-3,4,6,7,9,10-hexahydroacridine-1,8(2H,5H)-dione (3ac)

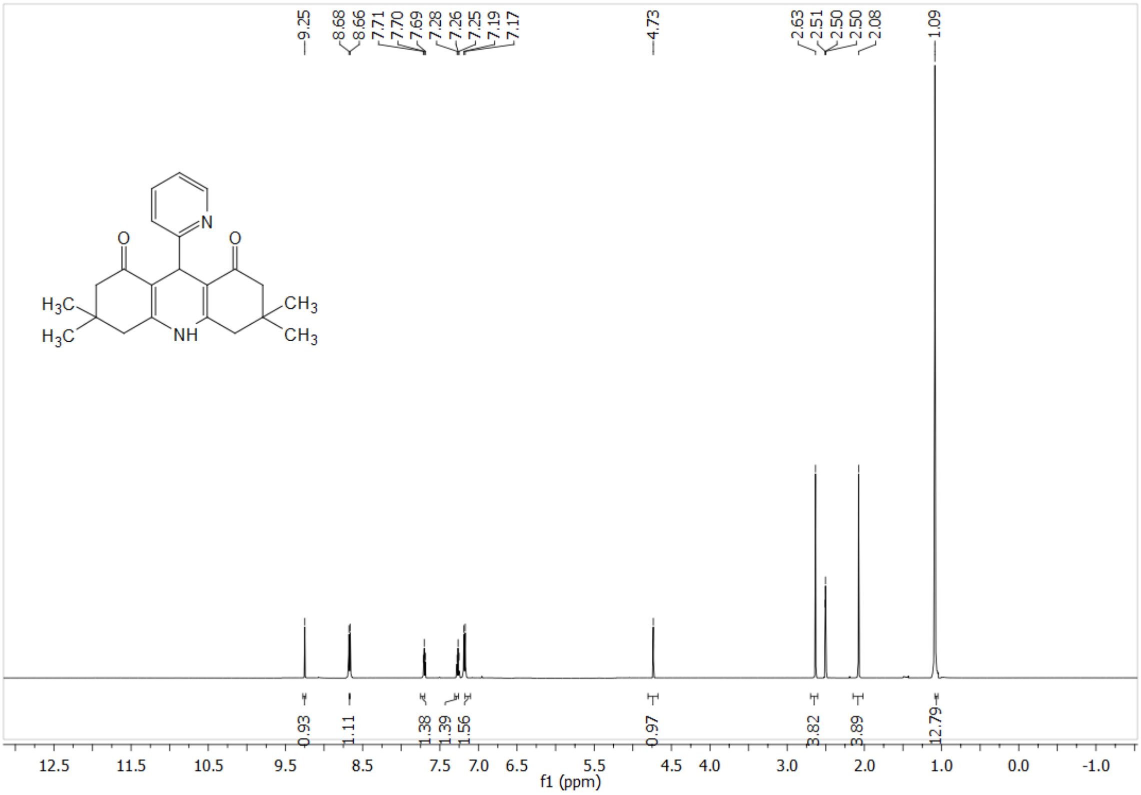

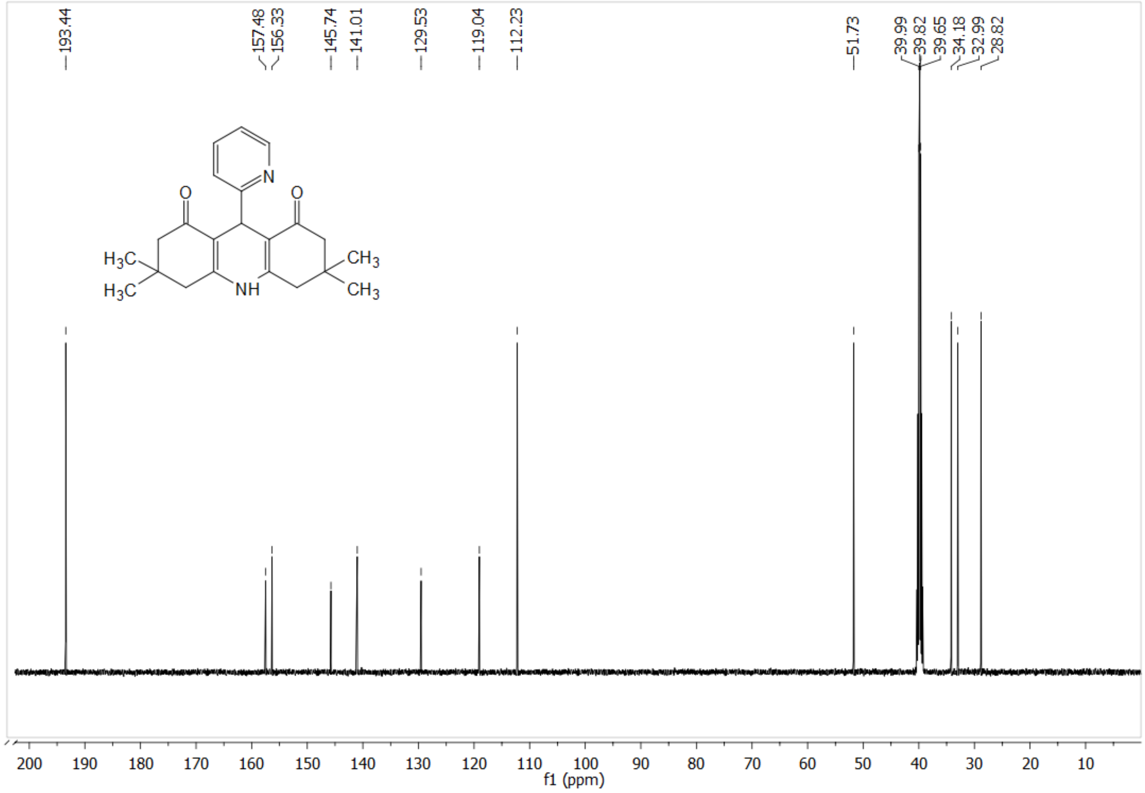

^{1}H and ^{13}C NMR Spectra of 2,2,8,8-tetramethyl-5-phenyl-5,10-dihydro-4H,6H-bis([1,3]dioxino)[4,5-b:5',4' e]pyridine-4,6-dione (3ad)

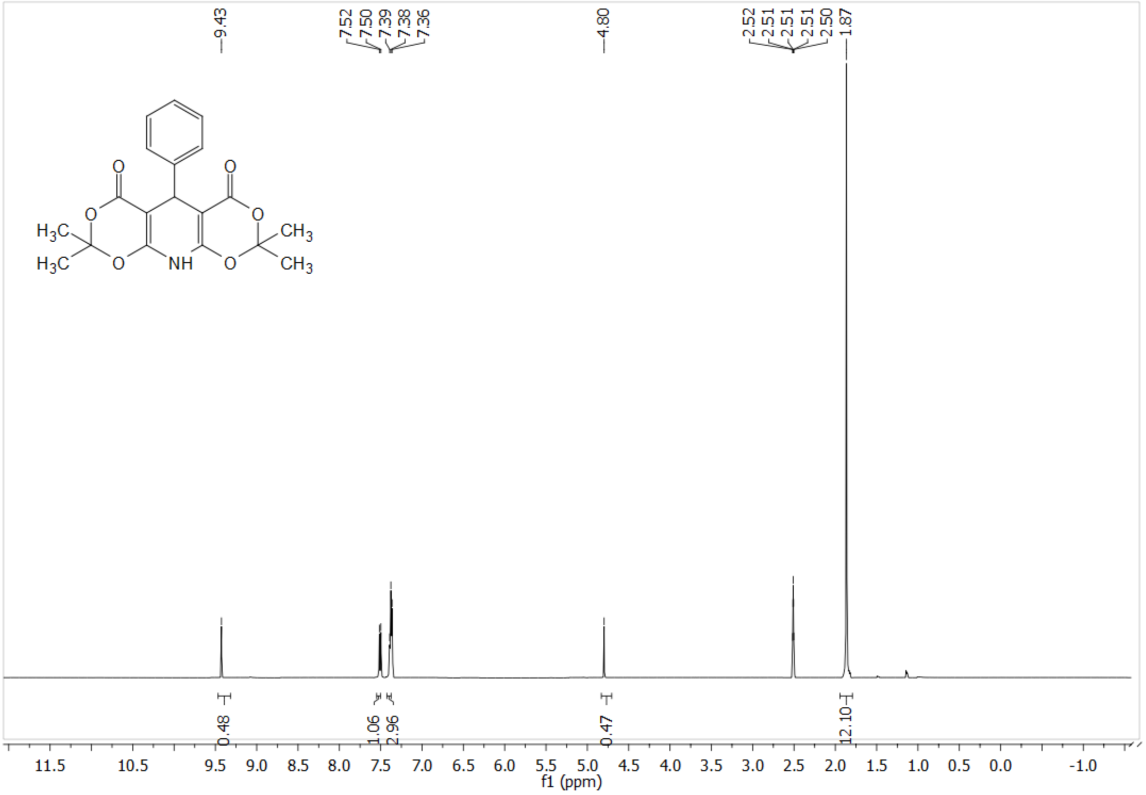

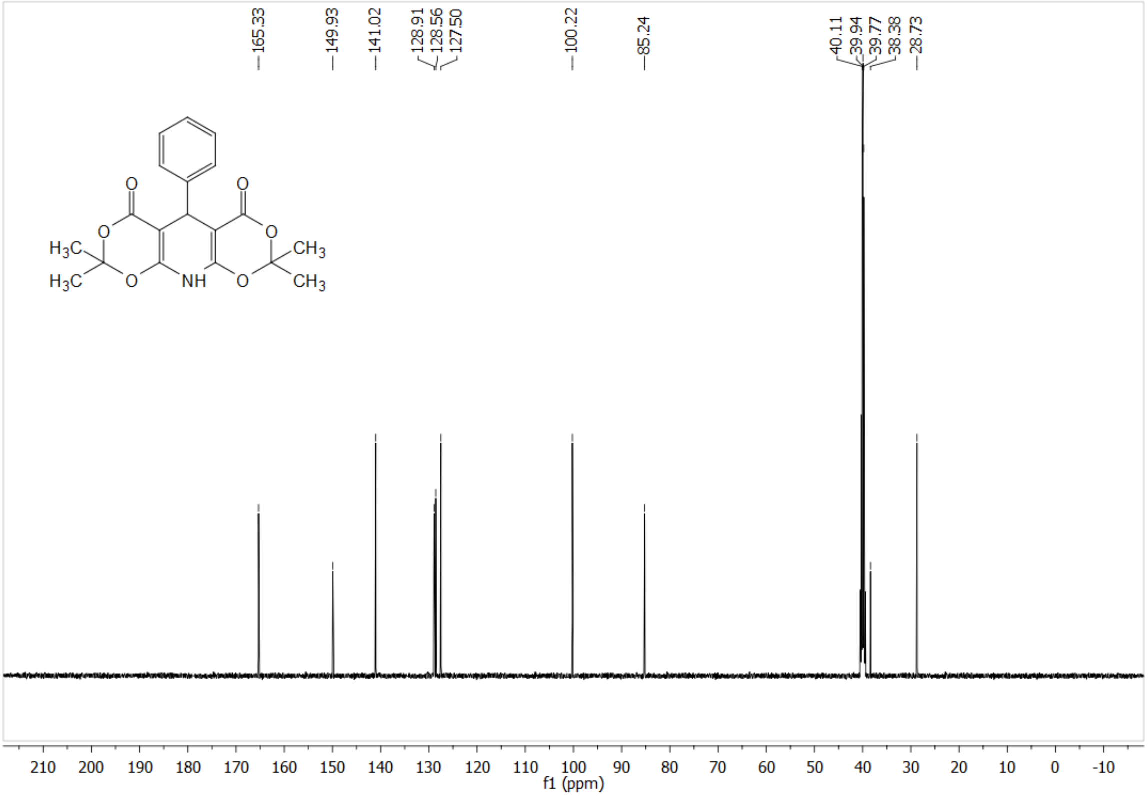

^{1}H and ^{13}C NMR Spectra of 5-(4-methoxyphenyl)-2,2,8,8-tetramethyl-5,10-dihydro-4H,6H-bis([1,3]dioxino) [4,5-b:5',4' e] pyridine-4,6-dione (3ae)

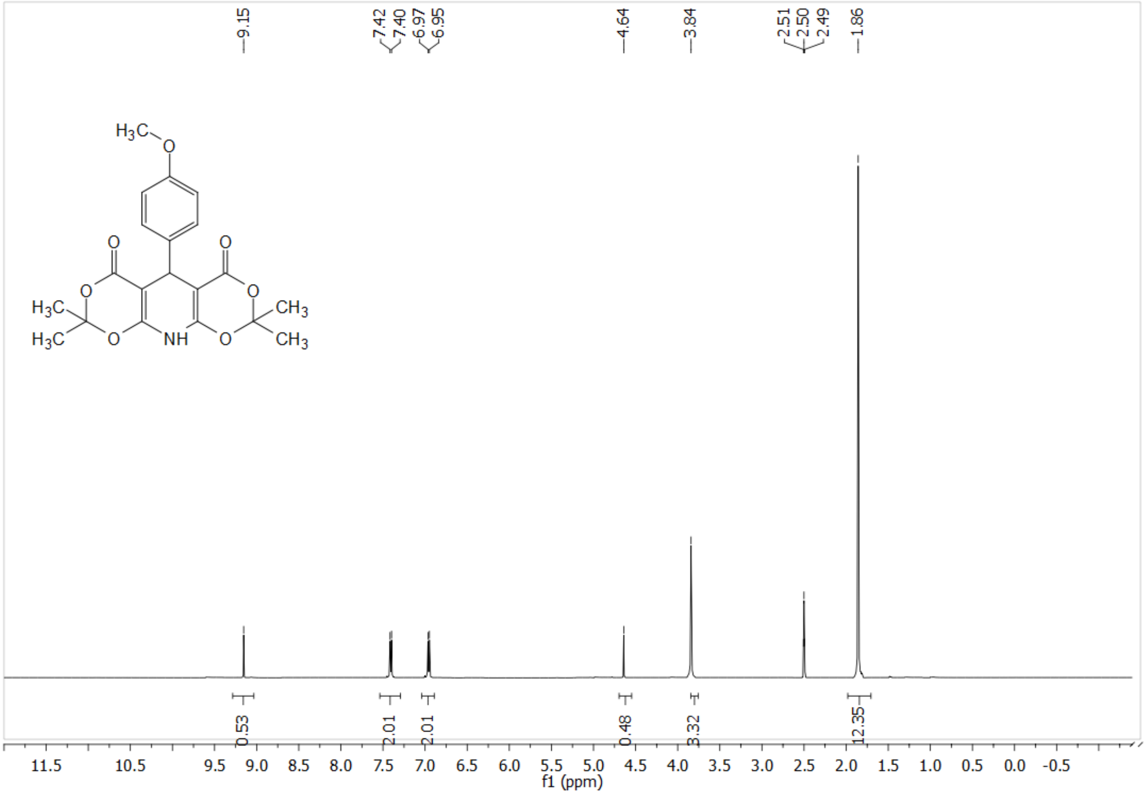

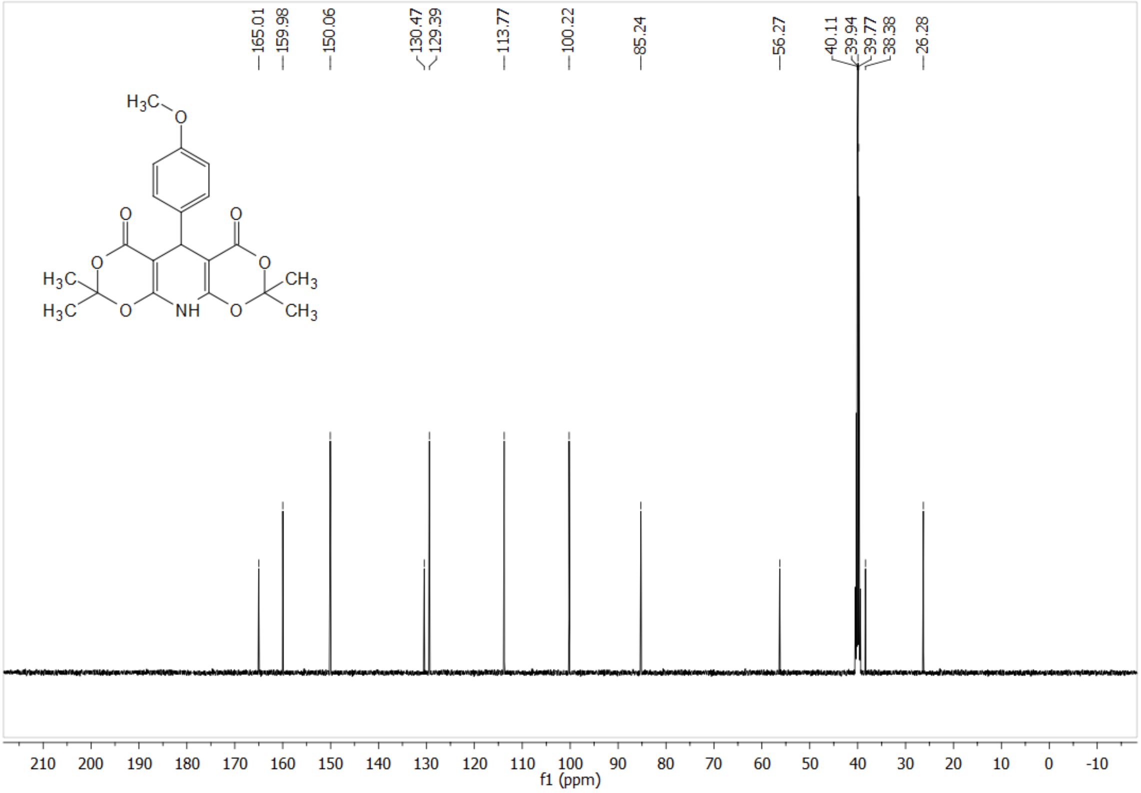

^{1}H and ^{13}C NMR Spectra of 5-(4-bromophenyl)-2,2,8,8-tetramethyl-5,10-dihydro-4H,6H-bis([1,3]dioxino)[4,5-b:5',4'-e]pyridine-4,6-dione (3af)

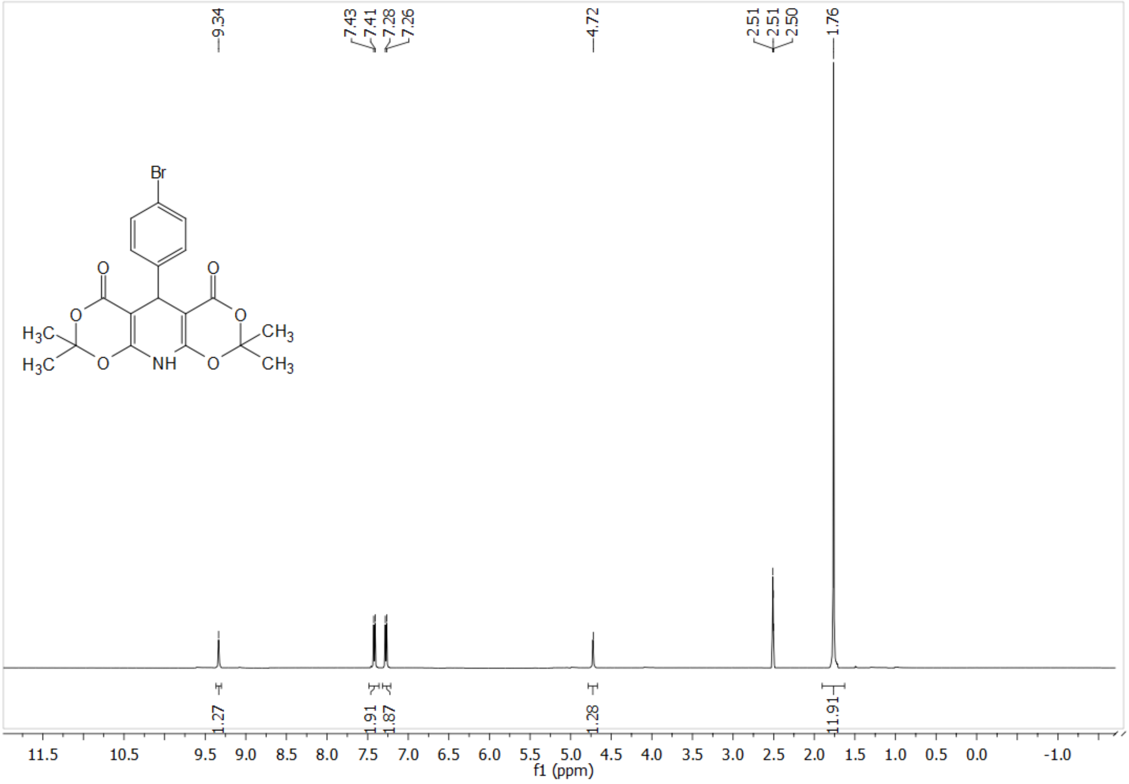

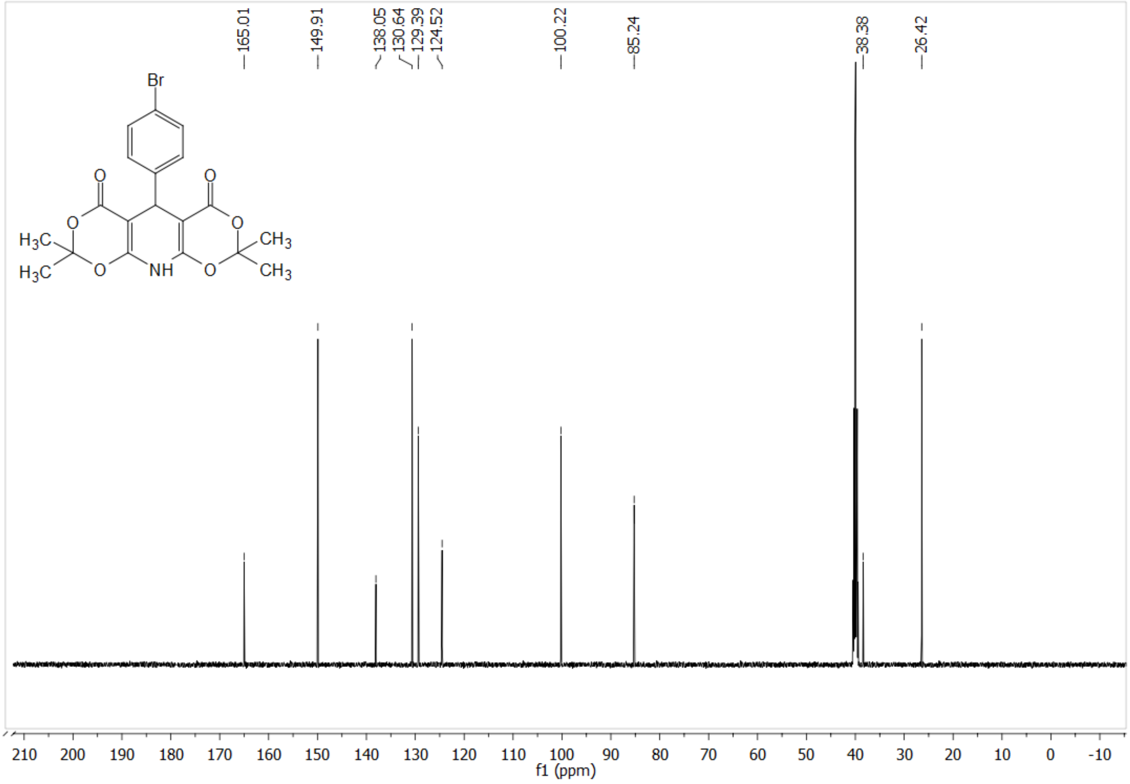

^{1}H, ^{13}C NMR and mass Spectra of dimethyl 2,6-dimethyl-4-(2-nitrophenyl)-1,4-dihydropyridine-3,5-dicarboxylate (3ag)

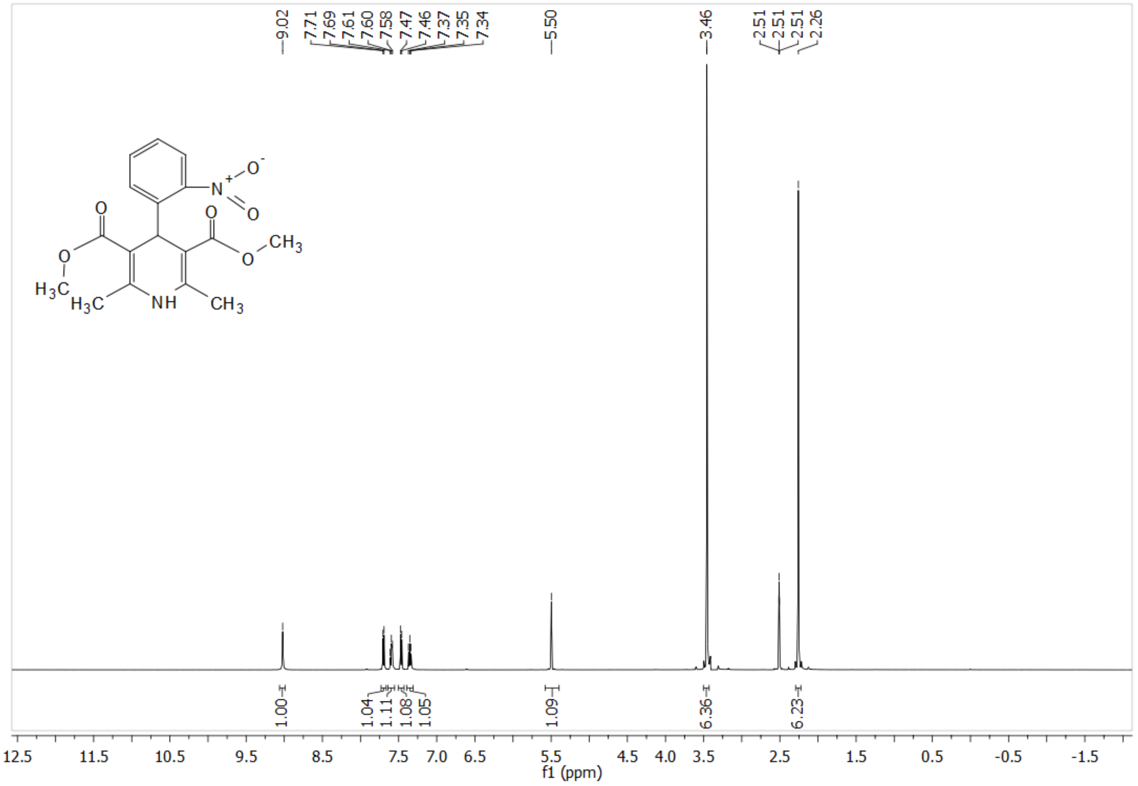

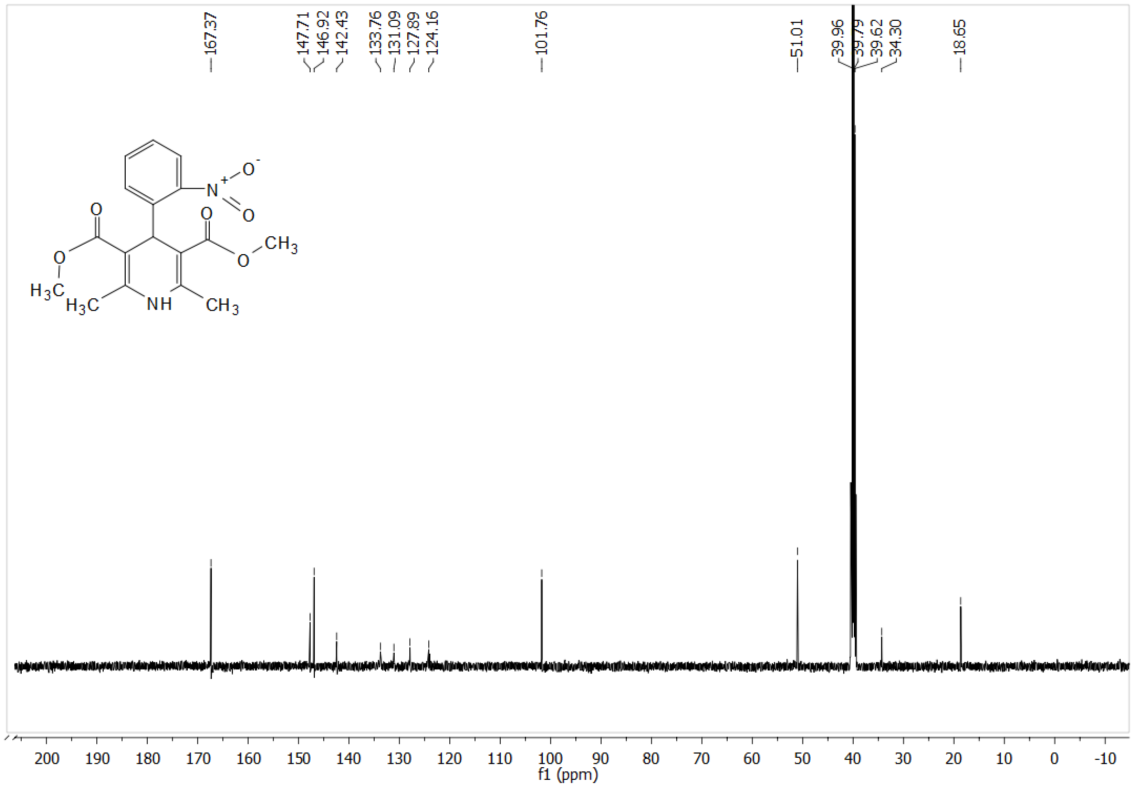

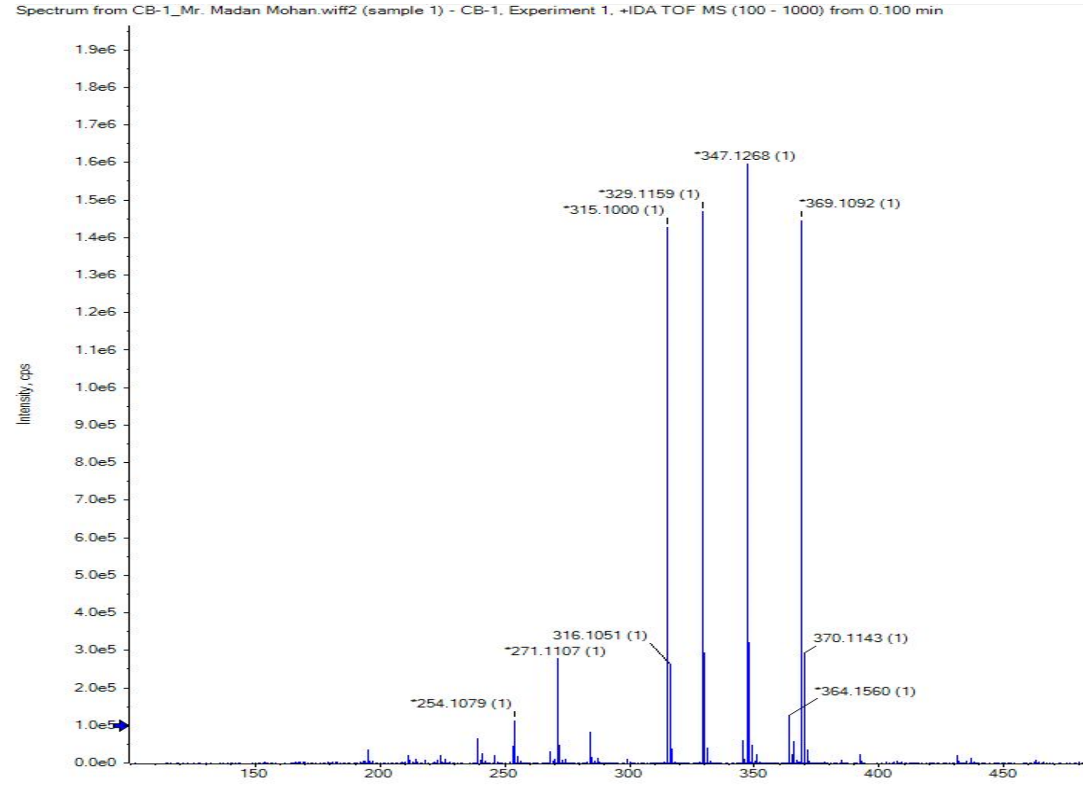

7. References

1 A. Karmakar, S. Banerjee, B. Singh and N. C. Mandal, *Journal of Molecular Structure*, 2019, **1177**, 418–429.

2 M. Patil, S. Karhale, A. Kudale, A. Kumbhar, S. More and V. Helavi, *Current Science*, 2019, **116**, 936–942.

3 H. T. Nguyen, V. A. Truong and P. Hoang Tran, *RSC Advances*, 2020, **10**, 25358–25363.

4 J. Zhang, W. Xue, P. Wang, T. Wang, Y. Liang and Z. Zhang, *Tetrahedron*, 2018, **74**, 4712–4720.

5 S. Karhale, C. Bhenki, G. Rashinkar and V. Helavi, *New Journal of Chemistry*, 2017, **41**, 5133–5141.

6 V. K. Sharma and S. K. Singh, *RSC advances*, 2017, **7**, 2682-2732.

7 M. G. Sharma, J. Pandya, D. M. Patel, R. M. Vala, V. Ramkumar, R. Subramanian, V. K. Gupta, R. L. Gardas, A. Dhanasekaran and H. M. Patel, *Polycyclic Aromatic Compound*s, 2021, **41**, 1495-1505.

8 M. Kidwai, D. Bhatnagar, *Chemical Papers,* 2010, **64**, 825–828.

9 M. R. Khumalo, S. N. Maddila, S. Maddila, and S. B. Jonnalagadda, *ChemistrySelect,* 2019, ***4***, 12503– 12506.

10 M. Mokhtar, T. S. Saleh, K. N. Rao, E. A. Mutairi, *Catalysis Today*, 2022, **397**, 484–496.
